# Single nuclei transcriptomics of muscle reveals intra-muscular cell dynamics linked to dystrophin loss and rescue

**DOI:** 10.1038/s42003-022-03938-0

**Published:** 2022-09-19

**Authors:** Deirdre D. Scripture-Adams, Kevin N. Chesmore, Florian Barthélémy, Richard T. Wang, Shirley Nieves-Rodriguez, Derek W. Wang, Ekaterina I. Mokhonova, Emilie D. Douine, Jijun Wan, Isaiah Little, Laura N. Rabichow, Stanley F. Nelson, M. Carrie Miceli

**Affiliations:** 1 Center for Duchenne Muscular Dystrophy at UCLA, Los Angeles, CA USA; 2 Department of Microbiology, Immunology, and Molecular Genetics, David Geffen School of Medicine and College of Letters and Sciences, University of California, Los Angeles, Los Angeles, CA USA; 3 Department of Human Genetics, David Geffen School of Medicine, University of California, Los Angeles, Los Angeles, CA USA; 4 Department of Neurology, David Geffen School of Medicine, University of California, Los Angeles, Los Angeles, CA USA; 5 Department of Pathology and Laboratory Medicine, David Geffen School of Medicine, University of California, Los Angeles, Los Angeles, CA USA; 6 Present Address: Amgen, Thousand Oaks, CA USA

**Keywords:** Skeletal muscle, RNA sequencing, Disease genetics, Preclinical research

## Abstract

In Duchenne muscular dystrophy, dystrophin loss leads to chronic muscle damage, dysregulation of repair, fibro-fatty replacement, and weakness. We develop methodology to efficiently isolate individual nuclei from minute quantities of frozen skeletal muscle, allowing single nuclei sequencing of irreplaceable archival samples and from very small samples. We apply this method to identify cell and gene expression dynamics within human DMD and *mdx* mouse muscle, characterizing effects of dystrophin rescue by exon skipping therapy at single nuclei resolution. *DMD* exon 23 skipping events are directly observed and increased in myonuclei from treated mice. We describe partial rescue of type IIa and IIx myofibers, expansion of an MDSC-like myeloid population, recovery of repair/remodeling M2-macrophage, and repression of inflammatory POSTN1 + fibroblasts in response to exon skipping and partial dystrophin restoration. Use of this method enables exploration of cellular and transcriptomic mechanisms of dystrophin loss and repair within an intact muscle environment. Our initial findings will scaffold our future work to more directly examine muscular dystrophies and putative recovery pathways.

## Abstract (web summary)

A method to isolate and sequence individual nuclei from human and mouse muscle biopsies provides further insight into the mechanisms of dystrophin loss and repair, in the context of Duchenne muscular dystrophy.

## Introduction

Duchenne muscular dystrophy (DMD) is caused by loss of function mutations in *DMD*, encoding dystrophin. Lack of dystrophin leads to contraction-induced myofiber injury, immune infiltration^1^, and, ultimately, replacement of myofibers by fat and fibrosis. Loss of myofibers promotes progressive skeletal, diaphragmatic, and cardiac muscle weakness and premature death. In healthy individuals, acute muscle injury triggers tightly coordinated immune cell infiltration and fibroblast expansion, essential for clearing damaged tissue and guiding satellite cell activation, differentiation, and muscle regeneration. Tissue remodeling occurs locally, at the site of injury, and resolves once the muscle is repaired. In DMD, chronic and heterogeneous myofiber damage results in dysregulation of immune- and fibroblast-coordinated muscle repair. Dystrophin replacement/repair strategies, including exon skipping, nonsense mutation readthrough, and micro-dystrophin gene therapy^2^, demonstrate some evidence of dystrophin replacement, muscle repair, and clinical benefit^3^. It remains unclear which cellular and molecular aspects of dysregulated muscle tissue remodeling are reversible by dystrophin rescue in the context of ongoing disease.

To demonstrate our sample sparing single nuclei sequencing technique, we used eleven archived frozen tibialis anterior samples from our published murine cohort, including four *mdx* mice, four *mdx* mice treated for 6 months with weekly exon 23 directed phosphoramidite morpholino oligomer (e23AON), and three genetically and age-matched C57BL/10ScSnJ controls. We have previously validated partial dystrophin rescue by western blot (ranging from 2 to 12% of wild-type levels) and reversal of pathology in *mdx* mice treated with e23AON in this cohort^4^. Similar findings using RT-PCR have also been reported in a ∆Ex51 transgenic mouse model^5^. Recently, gene expression has been observed within individual muscle cells or nuclei within heterogenous tissues, including human^6–9^, and mouse muscle^10–22^. Here we extend this work using archived frozen material. Small numbers of transverse cryotome sections of frozen muscle (~3 mg) were used to purify nuclei for high-quality single nuclei RNA sequencing (snRNAseq). We present here the largest single-cell/nuclei dataset of dystrophic muscle published to date. Using this single nuclei dataset, we identify cell regulatory processes involved in muscle degeneration, pathology, and repair in a DMD mouse model. We demonstrate that morpholino-induced dystrophin rescue partially reverses pathologic cell population and gene expression changes of myolineage cells, fibroblasts, and immune cell populations. Further, we apply the sample sparing nuclei isolation method to the study of archived frozen human muscle biopsies from healthy and DMD patients, producing, to our knowledge, the first snRNAseq report of human dystrophic muscle, and revealing substantial cellular heterogeneity and aberrant gene expression in human DMD.

## Results

**Fig. 1:**
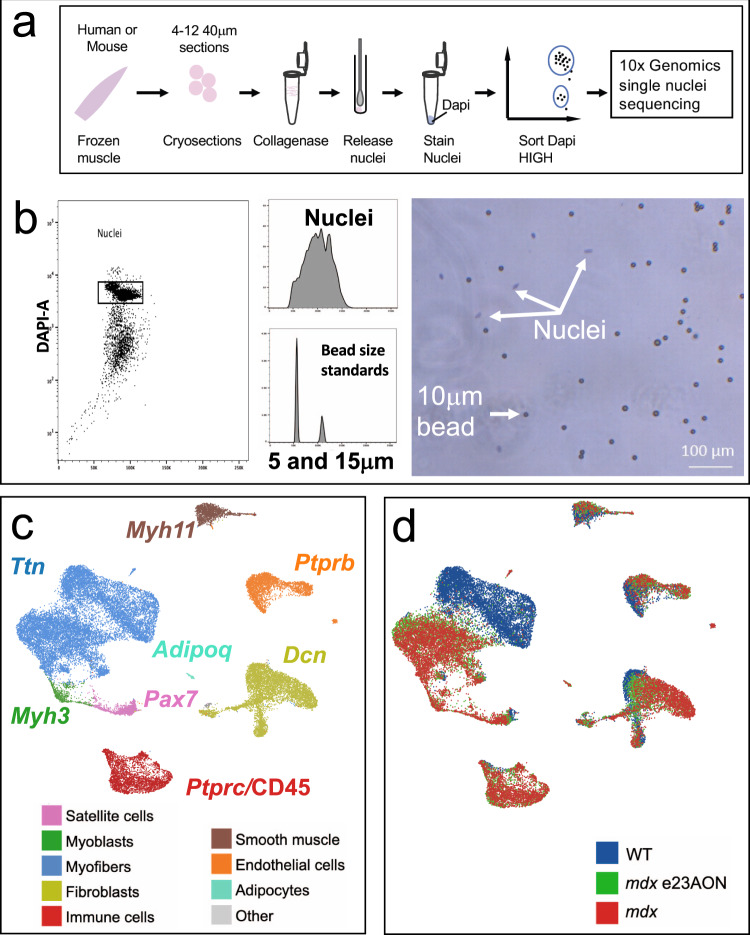
Single nuclei sequencing identifies cell types and disease-related transcriptomic changes in murine muscle. **a** Visual depiction of nuclei purification for snRNA sequencing. **b** Representative flow cytometric sorting of nuclei gated on DAPI staining (left panel), demonstration of forward scatter comparison to beads (middle panels), and size and morphology confirmation by visual microscopy (right panel). **c** Major clusters of cell types found in muscle were identified with a U-map analysis and subsequent identification of cell types using characteristic well known marker genes. One marker gene is shown for each cluster and text is colored to match the legend identifying major cell types of murine muscle. Subgroups with fewer members are listed in Other cell types panel, and include neural and schwann populations (gray). **d** Individual nuclei organized as the U-map identified clusters of **c** are labeled from mouse type: WT (blue), mdx (red), and mdx treated with e23AON (green).

To simultaneously assess gene expression of mono- and multinucleated cells within skeletal muscle, we developed a simple method of nuclei extraction from small quantities of frozen muscle tissue sections (Fig. 1a). Frozen tissues were disaggregated, and intact nuclei were sorted away from debris using flow cytometry. Even with the diversity of cell types within skeletal muscle, a single population of intact nuclei was identified with a size range from 7 to 10 μm (Fig. 1b). Quality and quantity of purified nuclei were verified (Fig. 1b) with yields from 3 mg of muscle ranging from 6000 to 100,000 nuclei.

Up to 20,000 nuclei/sample were loaded onto the 10X Genomics Chromium™ Controller Single Cell Sequencing System; ~35% of loaded nuclei resulted in viable libraries. The resulting libraries showed a single peak at ~450 bp and were sequenced to about 200 million paired-end reads per library, generating ~40,000 reads per nucleus. Eleven mouse tibialis anterior (TA) muscle samples yielded 34,833 single nuclei libraries, averaging 3167 nuclei libraries per sample. After normalizing sample read depth, samples averaged 39,334 reads per nucleus with 90% sequence saturation, resulting in 836 genes and 1371 UMI per nucleus, consistent with previously published reports of snRNASeq from muscle^18^. Nuclei with <200 genes per nucleus and the 4960 predicted doublets identified by DoubletFinder^23^ were removed from the dataset. Harmony was used to confirm identified populations were not the result of batch effects. The remaining mouse dataset contained 29,873 nuclei (9967 nuclei from *mdx*, 8411 from *mdx* e23AON, and 11,495 from WT muscle) with 846 genes per cell and 1396 UMI per nucleus. Five human vastus lateralis (VL) muscle biopsies generated a total of 7549 nuclei (4906 from DMD and 2643 from healthy individuals) with a median of 655 genes and 993 UMI per nucleus.

**Fig. 2:**
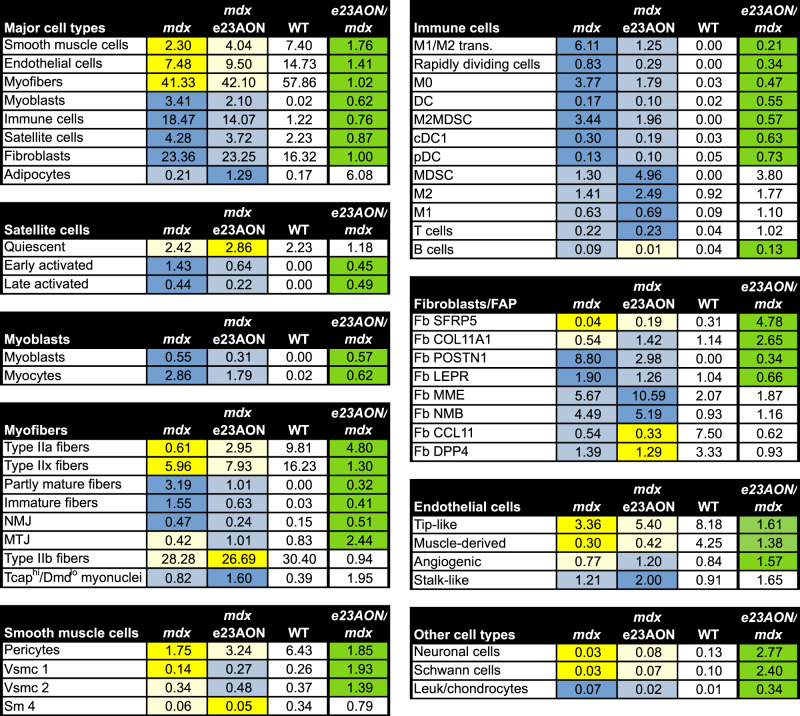
Frequencies of intramuscular cell populations in WT and *mdx* mice and partial normalization of most populations in response to e23AON treatment of *mdx*. Percentages of each cell population for each pooled treatment condition, *mdx* (*n* **=** 4), *mdx* e23AON (*n* **=** 4), WT (*n* **=** 3). Following the major cell types, cell types are further broken down to their respective subclusters. Blue = increase in percentage relative to WT, Yellow = decreased in percentages relative to WT. Rightmost column shows the effect of e23AON treatment relative to *mdx*, green denotes that cell numbers of *mdx* treated with e23AON recovered a more WT percentage.

We performed U-map analysis combining all 29,873 mouse single nuclei data, which enabled robust cell-type identification. Nuclei from 11 highly distinct cell types were identified based on similarity of gene expression including: satellite cells, myoblasts, myofibers, immune cells, fibroblast/fibro-adipogenic progenitors (FAP), smooth muscle, endothelial cells, adipocytes, neurons, and Schwann cells (Fig. 1c). These main populations were further refined by subclustering of nuclei, ultimately identifying 45 discrete clusters highlighting cellular heterogeneity of muscle tissue (Fig. 2). Some major cell populations are not the focus of further analyses including smooth muscle, endothelial cells, adipocytes, neurons, and Schwann cells. However, we note that the smooth muscle cells included nuclei from pericytes^24^, vascular smooth muscle cell type 1 and type 2^24,25^ (Supplementary Fig. 1, Fig. 2), and an uncategorized smooth muscle cell type (SM). Within the endothelial cells, we identified tip-like, stalk-like^26^, angiogenic, and muscle-derived endothelial cells (Supplementary Fig. 2, Fig. 2). Pseudotime lineage tracing analyses of endothelial and smooth muscle populations are shown in Supplementary Fig. 3.

### Myolineage psuedotime analysis reveals detailed developmental trajectory and confirmation of nuclei type identities

**Fig. 3:**
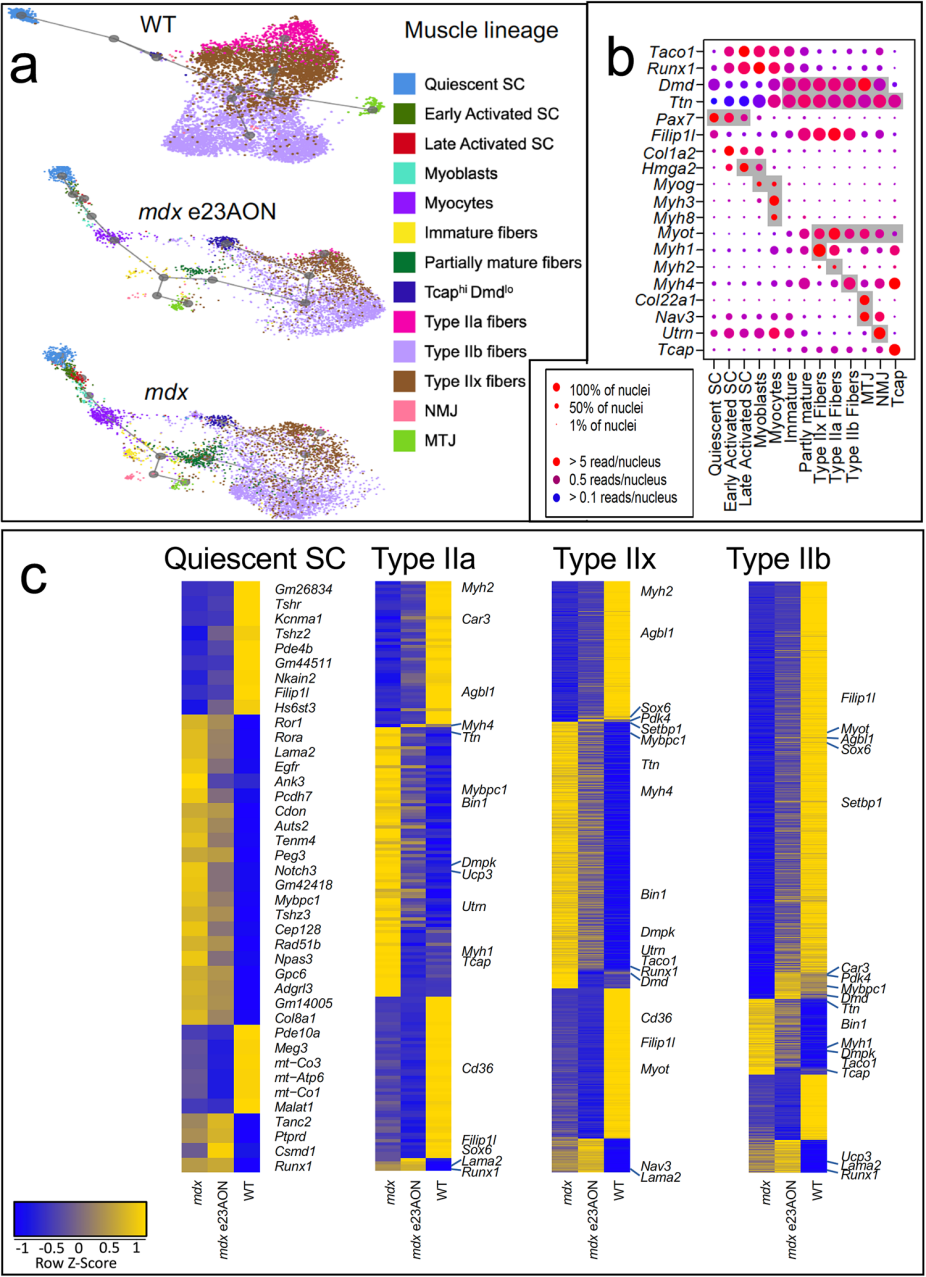
Murine muscle lineage disease-related cell type and transcriptomic changes. **a** U-map pseudotime analysis and lineage tracing of muscle cell types was performed for WT (*n* = 3), *mdx* e23AON (*n* = 4), and *mdx* (*n* = 4) nuclei. Muscle cell types are arranged according to their transcriptional relatedness and subcluster color is matched to legend for cell types shown in **a** and **b**. **b** Gene expression for selected genes distinguishing muscle cell subset populations plotted in **a**. Gray background indicates known markers of the respective cell type on *x*-axis. (Complete list of significantly different genes identifying each population is found in Supplementary Data 2). **c** Gene expression across treatment conditions is shown for select muscle cell/nuclei types. Yellow indicates higher gene expression. Heatmap of all genes significantly different between *mdx* and WT, demonstrates a strong trend toward a more WT pattern of expression in Satellite Cells, and Type IIa, IIx, and IIb myonuclei in e23AON-treated *mdx*.

We identified 13 clusters among skeletal muscle lineage nuclei (Figs. 3a, b, 2, Supplementary Figs. 4 and 5) reflecting satellite cells (quiescent, early, and late activation stage), myoblasts, myocytes, as well as multinucleated immature and partially mature fibers, type IIa, IIx, and IIb myofibers. We also identify myonuclei with specialized functions within myofibers, including neuromuscular junction (NMJ) nuclei, myotendinous junction (MTJ) nuclei, as well as Tcap^hi^/Dmd^lo^ nuclei, a population not previously described to our knowledge. No type I myofiber nuclei was observed, as expected for mouse TA.

To observe the relatedness of the 13 myolineage nuclei types, we performed pseudotime lineage tracing analysis on satellite cells, myoblasts, and mature muscle fiber^27^. Due to significant gene expression-based separation of myonuclei between *mdx* and WT mice, pseudotime lineage tracings were performed for WT and *mdx* mice separately (Fig. 3a, Supplementary Data 1). Based on the expression of genes known to be active at defined stages of myolineage development (Fig. 3b, Supplementary Data 2), the relatedness of these populations was consistent with established literature illustrating the transition from quiescent SC through activation, differentiation into myoblast, myocyte, and eventually into mature myofiber subtypes, including type IIa, IIx, and IIb myonuclei, NMJ, MTJ, and Tcap^hi^/Dmd^lo^ (Fig. 3a, b)^28^. Furthermore, quiescent *mdx* satellite cells cluster more closely to activated satellite cells, suggesting disruption of complete quiescence within *mdx* muscle relative to WT (Fig. 1c, d).

### Muscle remodeling occurs with dystrophin deficiency and partial rescue and is reflected in altered transcriptome and cellular composition among myolineage cell types

While WT and *mdx* lineage tracing show similar trajectories transitioning from quiescent SC (2.2% of total nuclei) through to mature fiber subtypes (10% type IIa, 16% IIx, and 30% IIb), WT lineage shows a noticeable lack of intermediary phases (*p*-value: early activated = 0.082, late activated = 0.018, myoblasts = 0.119, myocytes = 0.079, immature = 0.032, partly mature = 0.018) (Figs. 2, 3a), reflecting increased regeneration in *mdx muscle*. These intermediary phases, while still present in e23AON-treated *mdx* are noticeably reduced compared to untreated *mdx* (Figs. 3a, 2), suggestive of a reduction in muscle regeneration with e23AON treatment. Conversely, *mdx* had a substantial loss of mature myofiber nuclei (*p*-value: type IIa = 0.016, IIx = 0.012, IIb = 0.012), predominantly due to selective loss of type IIa (1%) and IIx (6%) myofibers relative to WT and were partly recovered with e23AON treatment (type IIa (3%) and IIx (8%)).

### Dystrophin loss and rescue impacts gene expression signatures in myolineage cell types

We assessed differential gene expression between *mdx* and WT mice nuclei, and between *mdx* and e23AON-treated *mdx* in each of the 13 identified myolineage stages. There was an overall trend that differentially expressed (DE) genes between corresponding populations in *mdx* and *mdx* e23AON, acquired a more ‘WT-like’ expression level with e23AON treatment.

In quiescent satellite cells (Fig. 3c), 75% (9/12) of genes significantly higher in WT relative to *mdx* were also upregulated in *mdx* treated with e23AON. Among these genes, *Pde4b* is reported to affect the severity of dystrophinopathy, and *Kcnma1*, and *Hs6st3* play roles in myogenesis (Supplementary Data 3b). Further, 84% (21/25) of genes significantly lower in WT satellite cells relative to *mdx* were also downregulated with e23AON treatment of *mdx*, suggesting a broad shift of expression in *mdx* e23AON toward the WT expression in this cell type (Fig. 3c). Many of these genes are associated with satellite cell activation (*Gpc6*), asymmetric division (*Egfr*), myoblast differentiation (*Runx1*), muscle growth/regeneration (*Cdon*, *Notch3*, and *Ror1*), muscle diseases (*Mybpc1* and *Lama2*), or aging muscle (*Gm42418*) (Supplementary Data 3b). Overall, the genes significantly upregulated in quiescent *mdx* satellite cells relative to WT (Supplementary Data 1) suggest that they are primed for activation, as many of these genes (*Gpc6, Runx1, Cdon, Notch3, Ror1, Mybpc1, Lama2,* and *Gm42418*) are also marker genes of early activated SC (Supplementary Data 2) (Supplementary data 3). The decreased expression of these genes in e23AON-treated *mdx* indicated a partial recovery towards WT physiology (Fig. 3c).

Gene expression of mature myofiber nuclei was the most perturbed in *mdx* relative to WT, consistent with myofibers being the primary site of dystrophin expression and is evident as a clear separation in UMAP plot of *mdx* myofiber nuclei type relative to WT for all fiber types (Figs. 1d, 3a). There was substantial similarity of gene expression difference across fiber types. For instance, 79% of genes differentially expressed in type IIx fibers were also differentially expressed in type IIb, similarly, 95% of genes differentially expressed in IIa are also differentially expressed in type IIb and type IIx, and the majority of these are also differentially expressed in MTJ, Tcap^hi^/Dmd^lo^, immature fibers, and partly mature myofiber nuclei (Supplementary Data 1). Many of these differentially expressed genes were also differentially expressed between *mdx* and e23AON-treated myofiber nuclei (68% in type IIx, 41% in type IIa, 78% in type IIb), with a strong trend toward e23AON-treated nuclei recovering a more WT gene expression (Fig. 3c). Many of the differentially expressed genes have been associated with muscular dystrophy (i.e., *Mybpc1* and *Dmpk*) (Supplementary Data 1, Supplementary Data 3b). Genes associated with improved stress recovery (*Car3, Agbl1,* and *Pdk4*) were only upregulated in myofibers from WT and e23AON-treated *mdx* (Supplementary Data 1, Supplementary Data 3b).

In *mdx*, myofibers misregulate genes required for proper terminal differentiation^29^. We identified *Sox6*, a gene essential for terminal differentiation of fiber types^29^, as suppressed in *mdx* IIx fibers (*p*-value = 8E-9, FC = 3.5, Fig. 3c, Supplementary Data 1) and IIb fiber nuclei (*p*-value = 1E-263, FC = 2.0, Fig. 3c, Supplementary Data 1) whereas *Myh2, Myh1,* and *Myh4* markers of Type IIa, Type IIx and Type IIb fiber nuclei respectively, were upregulated in *mdx*, but in myofiber types with different overall fiber typing (Supplementary Data 1, Supplementary Data 3b). e23AON treatment partially restored WT expression patterns, reducing expression of *Myh1* in IIb fibers (*p*-value = 1E-24, FC = 0.55, Fig. 3c, Supplementary Data 1) and *Myh4* in IIx fibers (*p*-value = 4E-13, FC = 0.77, Fig. 3c, Supplementary Data 1), and increasing the expression of *Sox6* in IIx fibers (*p*-value = 2E–52, FC = 1.5, Fig. 3c, Supplementary Data 1), and while not statistically significant, type IIa and IIb nuclei also trend in the same direction. These findings suggest that loss of dystrophin results in fiber dysmaturation which can be partially reversed with AON dystrophin rescue.

### Exon 23 skipped *Dmd* mRNA is observed at single nuclei resolution

In order to gain insight into mechanisms of degeneration and repair in dystrophic muscle, we compared cell and gene expression profiles of nuclei isolated from frozen muscles from WT control mice (*n* = 3), *mdx* mice (*n* = 4), and *mdx* mice treated with e23AON (*n* = 4)^4^. Systemic delivery of e23AON in *mdx* mice causes a portion of mature *Dmd* mRNAs to delete exon 23 (e23); removing the stop codon in e23 creates an in-frame mRNA that is translated into an internally deleted protein^4^. We retrieved all reads from snRNAseq fastq files that map to *Dmd* e22–e24 junctions (indicative of e23 skipping) and map their expression to nuclei within the myolineage developmental trajectory (Fig. 3a). While e22–e23 junctions were detected as early as the myocyte phase, e22–e24 junctions were only observed in mature myonuclei (Supplementary Fig. 6). As expected, both junctions were rare in our dataset (1.22% of myonuclei contain reads spanning e22–e23, and 0.08% of myonuclei contain reads spanning e22–e24) and are thus an under-observation of actual exon skipping^30^. The frequency of e22–e23 junctions was similar in both *mdx* and e23AON-treated *mdx*; however, the frequency of e22–e24 junctions was about 10× higher in e23AON-treated *mdx*. This is roughly consistent with dystrophin rescue of between 2 and 12% of WT dystrophin levels previously reported (Supplementary Figs. 7–15)^4^.

### Myeloid lineage trajectory analysis shows changes in response to loss and regain of dystrophin

**Fig. 4:**
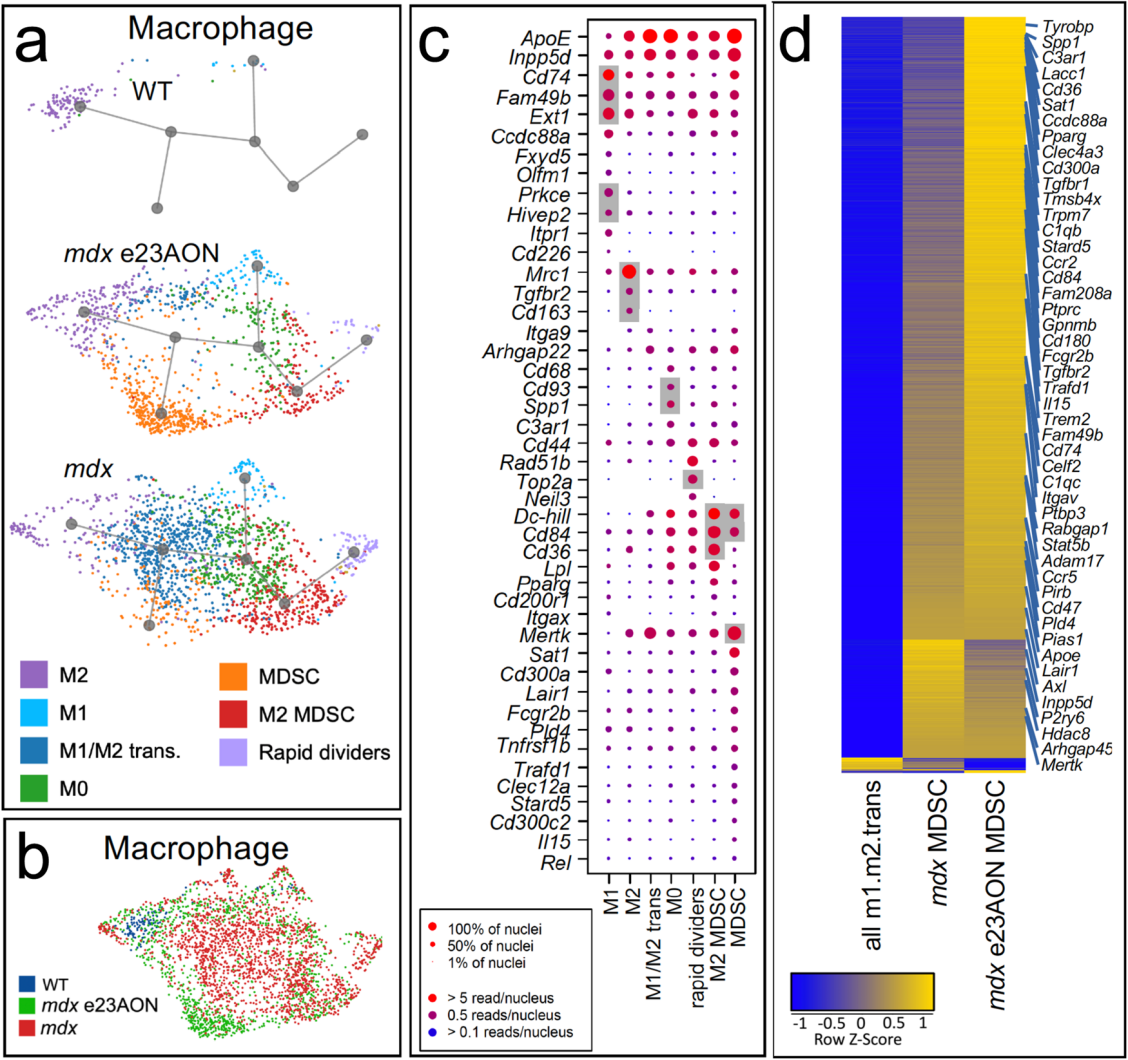
Expansion of anti-inflammatory macrophage populations with dystrophin loss and rescue. **a** U-map pseudotime analysis and lineage tracing of macrophages reveals subset types for WT (*n* = 3), *mdx* e23AON (*n* = 4), and *mdx* (*n* = 4). Macrophage subtypes are arranged according to their transcriptional relatedness and each subcluster color is matched to the legend for cell types shown in **a** and **c**. **b** Macrophage nuclei are organized as in **a** but color coded by mouse source as indicated. Gray background indicates known markers of the respective cell type on *x*-axis. **c** Gene expression for selected genes characterizing macrophage cell subset populations identified in **a**. (Complete list of significantly different genes is found in Supplementary Data 2). Dot size indicates percent of nuclei expressing the gene, dot color indicates level of expression. **d** Heatmap of gene expression changes across macrophage subsets and treatment conditions, including total M1/M2 transitional, MDSC from *mdx*, and MDSC from *mdx* e23AON, which are significantly expanded compared to untreated *mdx*. Yellow indicates higher gene expression. Heatmap shows the upregulation of MDSC characteristic genes as cells progress form M1/M2 transitional to *mdx* MDSC, and a further increase within MDSC with e23AON treatment (*mdx* e23AON).

Twelve different CD45 + immune cell types were identified; B cells, T cells, DC, cDC, pDC, M1-macrophage, M2-macrophage, M1/M2 transitional macrophage, two less well-differentiated macrophage cell types, termed M0 and rapidly dividing, and two cell types expressing several markers of myeloid-derived suppressor cells and negative regulators of inflammation, here referred to as MDSC, and M2MDSC (Figs. 2, 4a, c). Definitive identification of some of these myeloid populations, such as MDSC, will require functional assessment of these populations. MDSC resembles m-MDSC based on enriched *Ccr2*, *P2ry12*, *Pld4*, *F13a1*, *Ly86*, *Apoe*, *Il10ra*, *Lyz2*, *Il6ra*, *Mpeg1*, *C1qa*, and *Pip4k2a*, and M2MDSC resemble g-MDSC based on enrichment of *Psd3*, *Fn1,* and *Bcar3* (Supplementary Data 4). Genes that were significantly higher in each of these populations were used as markers to validate identity and are shown in Fig. 4c (macrophage cell types) and in Supplementary Fig. 16b (T cells, B cells, DC, pDC, and cDC). Complete lists of significantly expressed genes for each murine immune population can be found in Supplementary Data 2.

Myeloid immune cells and fibroblasts are known for their plasticity and responsiveness to environmental cues via activation of gene programs and differentiation^31^. Lineage trajectory analysis of expanded intramuscular myeloid nuclei found in the *mdx* dystrophic mice, indicates a high degree of relatedness (Fig. 4a, middle and lower panels), despite gene expression profiles reflecting distinct functionality (Supplementary Data 2), raising the possibility of *trans*-differentiation of one subset into an another in the context of differing muscle micro-environments (Fig. 4a). Consistent with reports that MDSCs arise from aberrant early myelopoiesis, the MDSC-like population was more distantly related to differentiated M1 and M2 effector populations, while the M1/M2 transitional cells were equally related to MDSC, M1, and M2, suggesting that M1/M2 are developmental intermediates (Fig. 4a).

Myeloid cells represented the majority of intramuscular immune cells. M2 macrophages were the primary population in WT muscle, representing less than 1% of total nuclei (75% of total immune cells in WT muscle) (Figs. 2, 4a). In *mdx* dystrophic muscle, multiple myeloid lineage cells were expanded and diversified (Fig. 4a) cumulatively representing 17% (*p* = 0.012) of total nuclei (Fig. 2), including M1-like (M1) (0.63% of total nuclei), M0 (3.78%), M1/M2 transitional cells (6.11%, *p* = 0.018), M2 (1.41%, *p* = 0.048), MDSC (1.3%, 0.037), and M2MDSC (3.44%, *p* = 0.048)^30,32^. Percentages of M0 (1.79%), M1/M2 transitional cells (1.25%), M2MDSC (1.96%), and B cells (0.01%) were substantially decreased with e23AON treatment, reflecting partial normalization of dystrophic intramuscular immune subsets toward WT composition (Figs. 2, 4a). e23AON treatment increased the frequencies of some cells with potential immune suppressive/anti-inflammatory properties, particularly MDSC (4.96%) and M2-macrophage (2.49%), driving them even higher than WT frequencies (Figs. 2, 4a). DC-, B-, and T-cell populations were also expanded in *mdx* relative to WT mice (Fig. 2). Heatmaps showing statistically significant genes for mouse immune cell subpopulations can be found in Supplementary Fig. 17.

We observed a substantial shift in the composition of the myeloid compartment in *mdx* versus *mdx* e23AON and WT (Fig. 4b), with ratios of M1/M2 transitional to MDSC in *mdx* inverting in response to e23AON-mediated exon skipping (Fig. 2, Supplementary Fig. 18a). To probe potential consequences of MDSC expansion and concomitant M1/M2 transitional cell contraction in e23AON-treated *mdx* muscle, we identified DE genes between these two populations (Supplementary Data 5, Fig. 4d). This analysis revealed upregulation of genes in MDSC relative to M1/M2 transitional cells corresponding to GO terms consistent with known roles of MDSC function including phagocytosis, autophagy, exosome function and other processes^33,34^ (Supplementary Fig. 18b, Supplementary Data 5). Further, regulators of: fatty acid oxidation (FAO) metabolism (*Sat1*, *AC149090.1, Stard5, Pparγ, Lacc1, Axl, Ddh, Lair, C1qc*, *C1qb Cd36,* and *Mertk*); immune suppression (*Trafd1, Cd180, Trpm7 Clec12a, Cd45, Pirb, Tyrobp, Trem2, Fcgr2b, Cd300c, Cd300a, Gpnmb Inpp5d*, *Il-10r, Tgfbr1, Ccr5, Itgav, Adam17,* and *Il15,Ccr2);* anti-inflammatory M2 skewing *(Tmsb4x, Hdac8, P2ry6, Ptbp3, Pias, Sirpα, Cd47,* and *Stat5b*) and muscle regeneration (*Igf1* and *Il15)* were all enriched in MDSC versus transitional M1/M2 cells. Of note, M2MDSC and MDSC populations both expressed genes characteristic of FAO, including *Lpl*, *Pparγ*, and *Cd36* (Supplementary Data 2, Supplementary Data 3e). Likewise, the M2 population present in WT and expanded in e23AON-treated muscle express genes characteristic of anti-inflammation and wound healing, including *Mertk*, *Cd36*, *C1qc,* and others (complete list of genes significantly associated with the M2 population are found in Supplementary Data 2).

Analysis of differentially expressed genes between MDSC and M1/M2 transitional populations indicated that e23AON treatment resulted in immune cell reprogramming towards a more MDSC-like gene profile (Fig. 4d): The average expression of genes upregulated in MDSC relative to M1/M2 transitional cells is further higher in MDSCs from *mdx* with e23AON treatment (Fig. 4d), indicating that e23AON treatment promotes conditions under which anti-inflammatory, regenerative/reparative and FAO promoting macrophage are maintained.

### Inflammatory fibroblast populations predominate in *mdx* muscle, while exon-skipping treatment reduces these populations

**Fig. 5:**
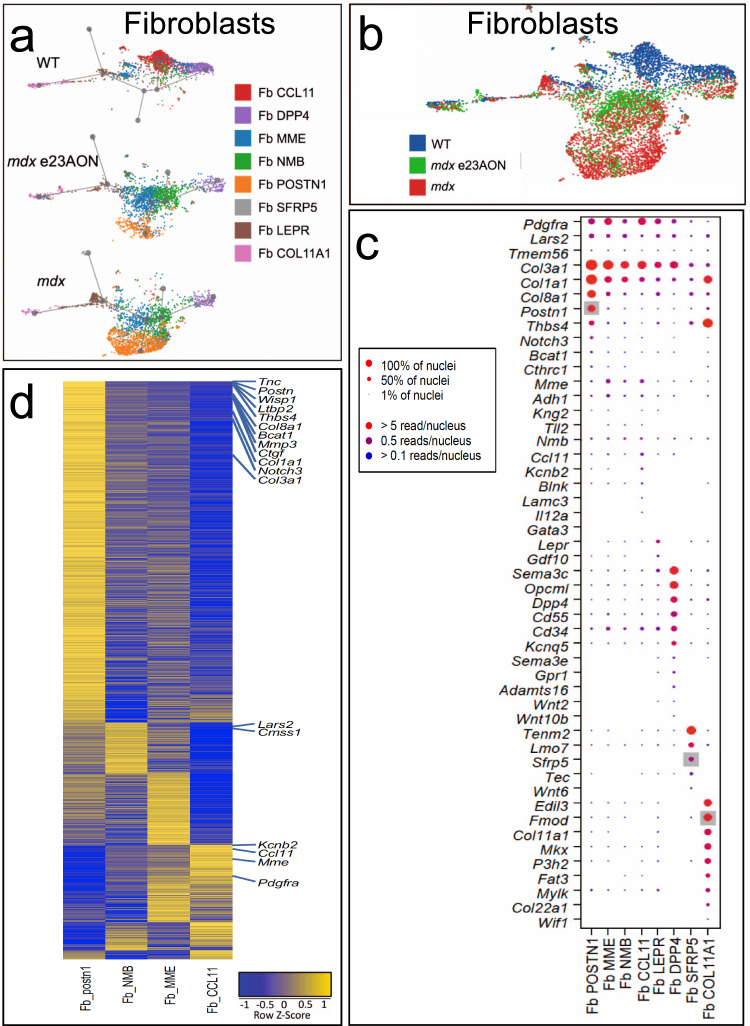
Inflammatory fibroblast populations dominate within dystrophic muscle while exon-skipping drug-treated muscle shows reduction of these populations and a transcriptomic return toward WT fibroblast patterns of gene expression. **a** U-map pseudotime analysis and lineage tracing of fibroblast subset types was performed for WT (*n* = 3), *mdx* e23AON (*n* = 4), and *mdx* (*n* = 4), with each fibroblast type labeled as a color and a prominent gene that is highly expressed as indicated. **b** All Fibroblast subtypes are arranged according to their transcriptional relatedness with each nucleus labeled by mouse sources as indicated. **c** Gene expression for descriptive genes characterizing fibroblast cell subset populations shown in **a**. Gray background indicates known markers of the respective cell type on *x*-axis. (Complete list of significantly different genes is found in Supplementary Data 2). Dot size indicates percent of nuclei expressing the gene of interest, dot color indicates level of expression. **d** Heatmap of gene expression differences between Fb CCL11 and Fb POSTN1 were generated for and these genes heatmaps shown for Fb NMB and Fb MME to demonstrate a transitional expression pattern in these cells between Fb POSTN1 and Fb CCL11.

Overall, fibroblasts make up a large portion of skeletal muscle in WT (16% of total nuclei) and are modestly increased in *mdx* (23% of total nuclei). However, gene expression-based lineage tracing of the fibroblast subpopulations revealed an increase in diverse fibroblasts in *mdx* relative to WT. The fibroblast cluster is characterized by fibroblast-specific gene expression of DCN and PDGFRA, which are known markers of fibroblasts, and other genes. Within the population of fibroblasts, eight fibroblasts/FAP subtypes were identified by subclustering, and each was named based on a relatively uniquely expressed gene (Supplementary Data 2, Fig. 5a, c, Supplementary Fig. 19) with a potentially relevant function: Fb CCL11, Fb DPP4, Fb POSTN1, Fb SFRP5, Fb COL11a1, Fb LEPR, Fb MME, and Fb NMB (Fig. 2). Several populations share features with fibroblast/FAP populations reported previously at the single-cell level, such as PDGFRA expression^35^. Fb COL11A1 is likely to represent a subset of the perimysial cells^35^ present at the myotendinous junction (*Col22a1* and *Mkx*). Fb SFRP5, which also express *Tenm2*, likely represent fibroblasts at sites of muscle attachment^36^ (Fig. 5c). As evident from the UMAP lineage tracing plots, Fb CCL11 and Fb DPP4 are least related to Fb POSTN1, and Fb NMB and Fb MME are located between Fb CCL11 and Fb POSTN1, suggesting that Fb NMB and Fb MME are intermediate *trans*-differentiation stages (Fig. 5a).

There was a substantial shift in fibroblast types between WT and *mdx*, and restoration toward a more WT distribution with e23AON treatment in *mdx* (Fig. 5b). Within fibroblast populations, Fb CCL11 predominated in WT muscle, and were 7.5% of all intramuscular nuclei (46.0% of all fibroblasts in WT muscle), whereas Fb CCL11 was only 0.5% of all nuclei in *mdx* muscle (*p* = 0.012, 2.3% of total fibroblasts in *mdx* muscle). Most notably, in *mdx* muscle, Fb POSTN1, a population not detected in our dataset of 11,485 total nuclei from WT muscle, was expanded to 8.8% of all nuclei in *mdx* muscle (37.7% of all *mdx* fibroblasts) (*p* = 0.017) as were Fb NMB (4.5% of all nuclei *mdx* vs 0.9% WT) and Fb MME (5.7% of all nuclei *mdx* vs 2.1% WT). Fibroblast populations shifted with induction of dystrophin by e23AON exon skipping: There was a contraction of Fb POSTN1 (8.8% of all nuclei *mdx* vs 3.0% *mdx* e23AON), and near doubling of Fb MME (5.7% vs. 10.6% of all nuclei, Fig. 2, Supplementary Fig. 18c). Based on pseudo-lineage tracing and gene expression (Supplementary Data 2), we infer that fibroblasts that are expanded in *mdx* represent *trans*-differentiation from WT muscle resident fibroblasts (Fb CCL11 and Fb DPP4) which are not proinflammatory to relatively more proinflammatory/profibrotic fibroblasts, particularly Fb POSTN1 (Figs. 2, 5a, Supplementary Data 3b)^37–39^.

We proposed that Fb MME and Fb NMB are intermediates between Fb POSTN1 and Fb CCL11 (Figs. 5a, d, 2) as there is a shift of differentially expressed genes between Fb CCL11 and Fb POSTN1, with Fb MME and Fb NME having intermediate expression for many of these genes (Fig. 5d), and these genes were shifted toward a more Fb CCL11 expression pattern with e23AON treatment: In Fb MME 87% (39/45) of genes upregulated in WT relative to *mdx* also had a higher average expression in *mdx* e23AON (Supplementary Fig. 20a), and 94% (16/17) of genes downregulated in WT relative to *mdx* were also downregulated in e23AON-treated *mdx* relative to *mdx* (Supplementary Fig. 20a). This pattern of treatment-induced partial recovery of WT gene expression is also observed in other independent cell types (Supplementary Figs. 20, 21). Genes upregulated with treatment in Fb MME include *Lama2* (loss is associated with muscular dystrophy^40^) and *Plxdc2* (inflammation suppressor^41^). Significantly downregulated genes in e23AON-treated *mdx* Fb MME include *Sparc*, *Meg3* and collagens, *Col3a1, Col1a1*, and *Col1a2*, which have been implicated in inflammation or fibrosis (Supplementary Data 1, Supplementary Data 3d, Supplementary Fig. 20a). Fb CCL11 fibroblasts show significant enrichment in the expression of genes with functions including wound healing and extracellular matrix (Supplementary Fig. 18d), while many genes enriched in Fb POSTN1, (Supplementary Data 6), were profibrotic/dystrophic in skeletal muscle, including, *Mmp3*, *Tnc*, *Col8a1*, *Bcat1*, *Ltbp2*, *Wisp1*, *Postn1,* and *Ctgf* (Supplementary Data 3d)^42,43^.

### Immunofluorescence staining of murine muscle confirms snRNASeq identification of cell types and e23AON treatment effects

To identify fiber type, immune, and fibroblast populations using protein markers, we stained tissue sections with antibodies directed against: (1) MYH2 to visualize fiber types IIa, (2) CD45 to visualize all immune cells, and (3) CD206 to specifically visualize M2 macrophages, and (4) POSTN1 to visualize areas of muscle tissue with a predominance of profibrotic/inflammatory fibroblasts.

**Fig. 6:**
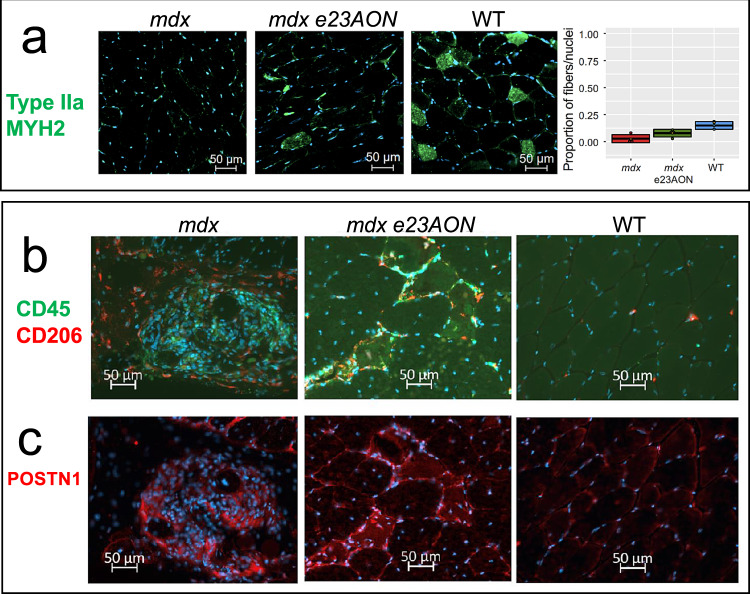
Immunofluorescence staining of muscle cross sections of some identified differentially expressed gene products. **a** MYH2 staining reveals preferential reduction in Type IIa Fibers in mdx and recovery in e23AON-treated *mdx*. Example immunofluorescence images of staining for each mouse are shown. MYH2 were stained (Green) in muscle sections from WT (*n* = 3), *mdx* e23AON (*n* = 4), and *mdx* (*n* = 4) mice, in combination with DAPI (blue) to mark nuclei. Quantification of the proportion of type IIa fibers was performed by counting the number of MYH2 + fibers out of >200 fibers per section from well preserved sections from each mouse (*mdx* *n* = 4, mdx e23AON *n* = 4, WT *n* = 3). Thick lines on box plot shows mean proportion across all samples in each treatment condition, box shows 1 standard deviation. Individual datapoints are shown as black dots on plot. Raw data values are listed in Supplementary Data 7. Red dot indicates the proportion myonuclei for each respective fiber type for each mouse type from snRNASeq. **b** e23AON treatment promotes M2 expansion. CD45 (green) and CD206 (red) were stained in muscle sections from WT (*n* = 3), *mdx* e23AON (*n* = 4), and *mdx* (*n* = 4) mice, in combination with DAPI (blue) to mark nuclei. Regions were chosen to best reflect number and type of immune cells present and differences in levels of immune infiltration occurring with disease and treatment. While untreated *mdx* mice show massive muscle infiltration of CD45 + non-M2 immune cells (green, **b**), e23AON-treated *mdx* mice show increased M2 cells as a percent of total CD45 + immune cells (red, **b**). **c** POSTN1 protein was probed in fibrotic lesions in sections from WT (*n* = 3), *mdx* e23AON (*n* = 4), and *mdx* (*n* = 4) mice. POSTN1 expression within fibrotic lesions was highest in *mdx*, and was reduced in *mdx* e23AON, and absent in WT animals.

Type IIa myofibers, measured from the MYH2 protein staining, make up 15% of myofibers in WT TA, and were substantially reduced (3% of myofibers) in *mdx* TA. With e23AON treatment, the MYH2+/type IIa myofibers were partly restored to 8% of myofibers, consistent with snRNAseq-based inferences (Fig. 6a). Visualization of CD45, CD206, and POSTN1 enabled observation of spatial relationships between M2 macrophages and POSTN1 marked fibrotic lesions. *mdx* TA show densely packed CD45 + immune infiltrates both within and around fibrotic lesions (Fig. 6b), whereas CD206 high expressing M2 cells are preferentially found surrounding the fibrotic area, (Fig. 6b). In *mdx* e23AON-treated, TA shows CD45 + and CD206 + M2 macrophages distributed around individual myofibers or in smaller POSTN1 marked fibrotic lesions, (Fig. 6b, c). WT muscle shows minimal fibrosis, with the predominant immune cells expressing the CD206 M2 marker scattered sparsely across the muscle (Fig. 6b, c). These results are concordant with our snRNAseq findings and highlight the value of assessing the spatial distribution of intramuscular cell subsets in guiding our understanding of dynamic relationships between immunity and fibrosis affecting skeletal muscle damage and repair in the context of loss of dystrophin.

### snRNAseq on frozen biopsy sections identifies diverse cell types and gene expression profiles in healthy and DMD human individuals

**Fig. 7:**
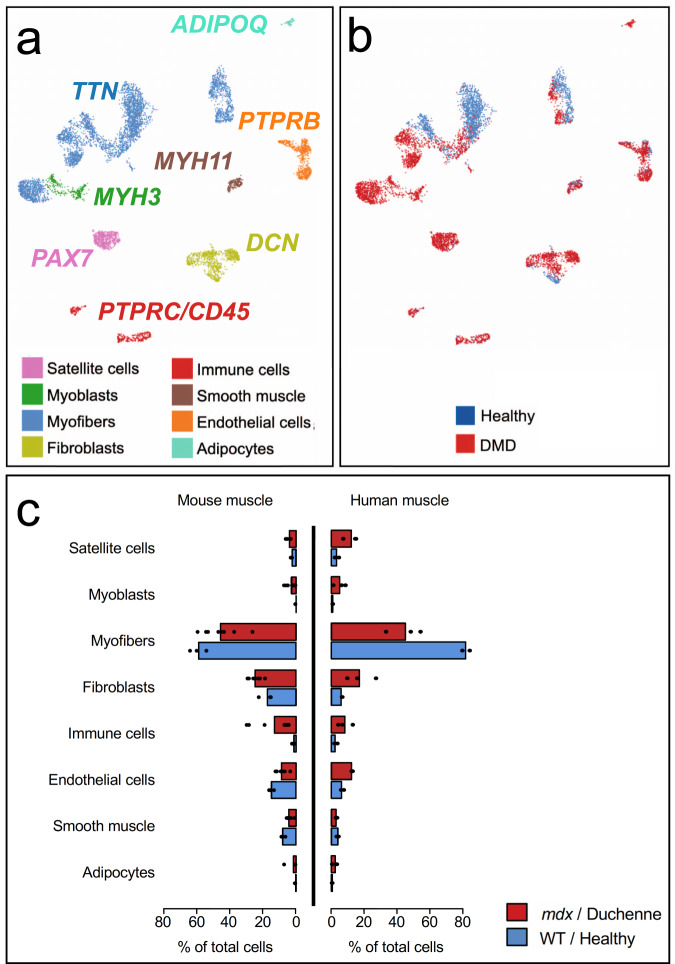
Identification of cell populations in human vastus lateralis from healthy and DMD individuals using snRNAseq and comparison with *mdx* and WT mice. **a** Umap clustering of human VL single nuclei transcriptomes. Major cell types were labeled using canonical gene markers common to mouse and human, and labeled as indicated for each major cell type (see Supplementary Fig. 22 for all human cell subtypes and frequencies for healthy and DMD). Genes significantly associated with each cluster and subcluster were identified and reported in Supplementary Figs. 23–27, and Supplementary Data 9. **b** Individual nuclei are labeled as from a DMD (red) or healthy (blue) and plotted as per the U-map clusters identified in **a** to show disease-related expression shifts. **c** DMD (*n* **=** 3) versus healthy (*n* **=** 2) and mdx (*n* **=** 8) versus WT (*n* **=** 3) comparison of snRNAseq identified major cell-type proportions are highly concordant. Bar plots indicate mean percentage of total nuclei belonging to each major cell type, individual datapoint are shown as black dots. Raw data values are listed in Supplementary Data 8.

We analyzed frozen human muscle biopsies from 3 DMD patients and 2 healthy controls, identifying 28 distinct cell types in VL muscle (Fig. 7a, Supplementary Data 9, Supplementary Figs. 22–27), broadly comparable to cell types identified in murine samples (Fig. 2). Overall shifts in cell populations were observed related to disease state (Fig. 7b). Like the comparison of *mdx* and WT, type IIa and IIx fibers and smooth muscle are reduced in DMD muscle compared to healthy controls, whereas satellite cells, immune cells, fibroblasts, and endothelial cells are increased in DMD muscle (Fig. 7c, and Supplementary Fig. 22). Both human and mouse showed an increase in adipocytes accompanying loss of dystrophin, though human dystrophic tissue had a greater increase in the proportion of adipocytes in the disease state, consistent with prior reports^44^. Upon subclustering of all samples, we identified six discrete myofiber nuclei types, four smooth muscle cell types, four types of macrophages, three subtypes of endothelial, and five types of fibroblast, two of which resemble previously reported subtypes, marked by *PRG4* and *LUM*^9^ (Supplementary Fig. 22). We identified a human Tcap^hi^/Dmd^lo^ population, in which 33 of 40 significant marker genes were shared with the mouse Tcap^hi^/Dmd^lo^ population, including *DES, CKM, TCAP, TNNC2*, and *ENO3* (Supplementary Data 9). In DMD subjects, fibroblast clusters Fb 1, 2, and 4 were expanded (Supplementary Fig. 22), and Fb 1 and Fb 2 expressed high levels of *CXCL12*, which encodes a chemokine known to recruit macrophages and T cells to muscle and is required for muscle regeneration^45,46^ (Supplementary Data 10). In addition, we confirm previously reported increased expression of *CXCL12* in DMD endothelial cells relative to healthy control^47^. In the *mdx* mouse, a profibrotic Fb POSTN1 population of fibroblasts was increased; in human dystrophic muscle, we confirmed that 11 of 31 genes significantly expressed in dystrophic human fibroblasts relative to healthy were also significantly expressed in the murine Fb POSTN1 fibroblast population, including *COL1A1, COL3A1, RAD51B, COL14A1, CCDC80, CDK14, CAMK2D, DCLK1, PRRX1,* and *LAMA4*. These shared DE genes suggest similarity in fibroblast transcriptional behavior between mice and humans within dystrophic skeletal muscle (Supplementary Data 2 and 10) and evidence of cell cycle entry combined with the production of collagens associated with fibrosis in this subtype of fibroblast.

Within the human macrophage/myeloid immune compartment, we identified putative M1, M2, pDC, and MDSC-like subpopulations (Supplementary Fig. 22, Supplementary Data 9). These populations have the mixed, labile quality previously reported for in situ tissue macrophage, in that they express markers consistent with multiple canonical cell types^48,49^. Mac 1 was most consistent with an M2-macrophage, expressing *CD14*, modest levels of *FCγRIIIa/CD16*, as well as M2 markers *CD23/FCER2*, *CCL18*, *CD209,* and *F13A1*^48^ *(*statistically significantly upregulated relative to other clusters), though we also identified markers more typical of an inflammatory state, such as *IL-2RA*^50,51^. Mac 2 most resembled M1 macrophages, expressing *CD14*, and M1 markers; *CD44 (osteopontin receptor), CD86*, *TLR2*^52^*, IL-15*^48,52^, and highest levels of *FCγRIIIa/CD16*^48^. Mac 3 is potentially a proinflammatory pDC population, most clearly marked by significantly upregulated pDC marker *HFM1* and *FAAH*^53^ and Human protein atlas (https://www.proteinatlas.org/). Heatmaps for all human subsets, including immune cells, are available in Supplementary Figs. 23–27. Mac 4 most resembled MDSC or other immune suppressive/reparative macrophages among myeloid cell types in that it was the major population to express *GPNMB*/DC-HIL, which encodes a protein characteristic of MDSC and involved in T-cell repression^54^, and S100A4, critical for MDSC survival^54^. This same cluster, in the DMD samples only, expressed other immune-modulatory and/or profibrotic genes, such as *SPP1*, expressed exclusively in Mac 4^55,56^ and *TGFB*^57^ as well as proinflammatory genes, including *CD64*/*FCGR*A and *CD64b/FCGR1b*^48,58^ (Supplementary Data 9). Both MDSC and the M1 populations were increased in DMD muscle relative to healthy control muscle (10-fold and 3-fold, respectively) (Supplementary Fig. 22), highlighting commonalities between human and mouse (Fig. 2) dystrophy.

## Discussion

Because some cells are multinucleated (myofibers) while others (immune, fibroblast, satellite) are mononuclear, we implemented protocols for unbiased sampling from all nuclei types resident in muscle to reveal the complex architecture of skeletal muscle in health and disease. We developed and implemented a method to purify individual nuclei from approximately 3 mg of a cryosectioned intact frozen mouse or human skeletal muscle for single nuclei sequencing. This is a powerful means to: (1) reveal the heterogeneity of cells that compose skeletal muscle, (2) identify novel cell types and myofiber nuclei of specialized function, and (3) observe and quantify substantial shifts in intramuscular cell types and gene expression.

We identified eight mouse and five human subpopulations of myofiber nuclei, some reflective of specialized nuclei functions (NMJ and MTJ), and provide comparative data on immature, partially mature, type I, IIa, IIx, and IIb myofibers and four smooth muscle subpopulations. We further describe a Tcap^hi^/Dmd^lo^ myonuclei population that appears distinct from other recently described myonuclei subsets from prior single nuclei studies^15,18^. While the full functionality of this myonuclear subtype is undescribed, the TCAP protein is necessary for sarcomere-membrane interactions and can be localized to z-disks in skeletal muscle^59,60^, and mutations in the *TCAP* gene are known to cause various forms of muscular dystrophy^60–62^.

In both mouse and human muscle, lack of dystrophin is associated with chronic myofiber damage, and we show a common depletion of type IIa and IIx myofibers in mouse TA and human VL, with similar overall patterns of activation, expansion, and diversification of satellite cells, myoblasts, immune cells, fibroblasts, and other populations^63^. While we were able to identify an increase in rare exon-skipping events induced by e23AON in individual nuclei, the frequency of observation was too low to compare skipped versus unskipped myonuclei with statistical significance, though this advance opens the door to future experiments of this type as we progressively increase our sensitivity using this technique. It is unclear whether the effects of dystrophin rescue on satellite cell dynamics reflect rescue of dystrophin within satellite cells, where it is proposed to control asymmetric division, or due to decreased activation secondary to myofiber rescue. Our lack of observed exon skipping in satellite cells may reflect a relatively low frequency, the relative scarcity of skipped messages, or limits to our detection. Thus, we cannot exclude satellite cells as a skipping target. Technical improvements to capture and sequence full-length *DMD* will improve the utility of snRNAseq in DMD muscle to address this question at single-cell resolution.

In contrast to most immune cells, which are reduced in treated vs untreated *mdx*, a select myeloid subset, termed MDSC, is substantially expanded in e23AON-treated *mdx* muscle. The most common immune cell type found in WT muscle, M2, also increases almost two-fold in treated mdx mice, demonstrating a treatment-induced trend of return toward WT functionality. In addition to its dominance of the wild-type muscle, the M2 immune cluster is the only immune cell cluster we identify here which expresses Timd4, recently reported to be associated with a locally self-renewing population of muscle resident macrophages which are uniquely responsible for clearance of apoptotic damaged muscle following acute injury^49,64,65^. While there was some overlap of significant genes (9 each out of a total of 63) in MDSC and M2MDSC with the reparative GDF-15 expressing macrophage populations recently reported by Patsalos et al., this does not seem to be an exact match despite common functions including immune suppression, and FAO^66^.

M1/M2 transitional cells, the primary *mdx* immune population observed, contracts with treatment and takes on a more MDSC-like gene expression profile. Given the relatedness of these populations, we propose that exon-skipping treatment might drive M1/M2 transitional cells to become MDSC and/or M2 cells.

While the initial proinflammatory response to muscle damage is essential for satellite cell activation and myogenesis, a switch to immune mechanisms of anti-inflammation and wound healing guides the completion of muscle repair^67^. By sensing fatty acids, PPARγ can trigger a metabolic shift within immune populations, resulting in a switch from glycolytic to OXPHOS metabolism for energy production and select differentiation and activation of M2 macrophages, MDSC, and other immune cells associated with immune suppressive, anti-inflammation and tissue repair^68,69^. Likewise, iC1q and LAIR-1 upregulation can promote the switch from pro- to anti-inflammation by recruiting MERTK-dependent production of specialized pro-resolving mediators and IL10^70^. Multiple members of these cascades are upregulated in intramuscular MDSC and M2 cells and are increased further with exon skipping and dystrophin rescue, supporting their potential role in facilitating a metabolic shift toward a more healing and anti-inflammatory muscle microenvironment and identifying potential molecular targets for disease modulation.

This initial description of intramuscular cell populations within human DMD and *mdx* mouse muscle provides a framework for a more complete analysis across many dystrophic samples. A deeper characterization of human skeletal muscles with variable disease course, or with longitudinal sampling in response to therapeutic strategies, will more fully describe the cell types and variations in specialized nuclei types within multinucleated myofibers. Our ability to identify cell composition and gene expression profiles in nuclei prepared from very small (3 mg) samples of frozen human muscle now make feasible snRNASeq analysis on human remnant muscle tissue obtained in the context of dystrophin replacement or other DMD therapies, natural history studies, or from small core needle biopsies of unusual cases, enabling exploration of therapeutic and naturally occurring mechanisms of genetic repair and pathogenesis in DMD and other muscle conditions.

## Methods

### Mouse study design

Eleven 32–36-week-old male mice were selected from a published *mdx* mouse experiment in which replicate mice were treated weekly with antisense morpholino e23AON (5′-GGCCAAACCTCGGCTTACCTGAAAT-3′^71^) over 6 months to induce exon skipping and dystrophin expression in skeletal muscle^4^. Archived frozen tibialis anterior muscle, stored in LN2 from four e23AON-treated *mdx*, four age-matched untreated *mdx,* and three age-matched untreated control C57BL/10ScSnJ mice (WT) were compared^4^. All animal handling was performed in accordance with applicable national and institution-specific guidelines: UCLA Animal Research Committee (ARC) and Animal Use Committee protocol ARC # 2011-021-21.

### Human study design

Human muscle was obtained by needle biopsy and stored as previously reported^72^. Informed consent was obtained for all human patients (UCLA IRB approvals 18-001366 or 19-00090) who provided muscle tissue via open biopsy or using vacuum-assisted needle biopsy. Ultra-sonagraphy was used to avoid neurovascular structures. Multiple cores, of ~100 ug in size were obtained from TA or VL muscles. Briefly, 3–10 ml of 1%lidocaine was administered intradermally, sub dermally for each muscle selected. Typically, biopsy material was collected from two different muscles per patient if possible. Sedation was used for children between 2 and 7 years of age (IV fentanyl and propofol at standard doses, with cardiorespiratory monitoring until alert) and was administered by a pediatric intensivist or anesthesiologist.

### Microscopy and immunochemistry

Dystrophin staining was performed as previously described^4^. IHC was performed on unfixed 10 μm tissue sections using the MouseOnMouse kit (Vector Labs). IHC assessment used the following primary antibodies: MANDYS8 (dystrophin rod domain) (Sigma–Aldrich, SAB4200764, ascites fluid, 1:400 dilution) and DNA with DAPI. Secondary labeling was performed with fluorescein isothiocyanate (FITC) anti-mouse or FITC anti-rabbit (Vector Labs). Sections were mounted in Vectashield Mounting Medium (Vector Labs). Fluorescent images were acquired and analyzed using Ariol SL-50 (Applied Imaging, San Jose, CA). The Ariol scanner is based on an Olympus BX61 microscope with motorized stage and autofocus capabilities, equipped with a black and white video camera (Jai CVM2CL). Scanning and analyses were performed through the Translational Pathology Core Laboratory, Department of Pathology and Laboratory Medicine, David Geffen School of Medicine at UCLA.^4^.

Trypan blue images of nuclei and size beads were obtained using a Zeiss Axiovert 40 CFL, scaled as indicated in Fig. 1a. For immunofluorescence detection of immune cells: Cross-sections (10 μm) on glass slides were fixed in 4% paraformaldehyde, solubilized with 0.5% triton, blocked with 3% BSA for 1 h, prior to application of primary antibodies. Antibodies used were as follows: anti-CD45; rat, 30-F11 (ebiosicences) (10 μg/ml) followed by donkey anti-rat Alexa 488 (13.4 μg/ml), CD206; goat polyclonal (R&D) (10μg/ml) followed by Rabbit anti-goat Alexa546 polyclonal (Invitrogen) (2 μg/ml), POSTN1; Rabbit polyclonal (Thermo Fisher) (20μg/ml) followed by Goat anti-rabbit dylight 550 (4 μg/ml), MYH2; 8F72C8 (Sigma–Aldrich) (40 μg/ml) followed by donkey anti-rat Alexa 488 (4μg/ml). Primary antibodies were incubated overnight, washed with 0.1% triton four times prior to 1 h incubation with secondary antibodies, and sections mounted with vectashield DAPI mount. Staining for MYH2 was blocked for 5 hours prior to applying primary antibodies.

### Nuclei extraction from fresh frozen tissue

Cryosectioning was used to efficiently release nuclei from difficult to disaggregate muscle fibers: 40 μm section thicknesses were chosen to maximize isolation of intact nuclei of the size range of 7–10 μm. Frozen muscle was cryosectioned into 4–12 40 μm thick cross sections (using the lower number of sections for larger diameter muscle pieces, and the higher number of sections for very small diameter pieces) into a 1.5 ml tube on dry ice to provide a total of ~3 mg of tissue. 0.5 ml of an ice-cold solution of 0.2 μm filtered 1% BSA in PBS with 100U/ml of type IV collagenase (CAT: #07426, 100 mg from Stem Cell Technologies) and 0.5U/μl RNAse inhibitor (RNAse protector, Sigma Ref # 03335402001, Mannheim, Germany) was pipetted onto the sections, and gently pipetted up and down, transferred to a small glass dounce, and kept on ice 10 min; pieces were allowed to settle to the bottom before douncing. Seven strokes were performed slowly, being sure to trap sections, with an A-gap dounce (disaggregate tissue), followed by seven strokes with a B-gap (subcellular gap size), avoiding bubbles. The mixture was subsequently filtered through a 70 μm filter, and the effluent was centrifuged at 600 × *g* for 6 min at 4 °C. Pellets were resuspended in 0.5 ml of 1% BSA in PBS with RNAse inhibitor (0.5U/μl) and DAPI (10 μg/ml), incubated on ice for 60 min, then sorted. A small amount of each sample being sorted was saved aside to be pooled for a negative and positive control staining tube to use to set up the sort.

### Nuclei sorting

Nuclei were sorted using a BD FACS ARIA II sorter with UV laser and 70μm nozzle. Nuclei were gated for aggregate removal by size using the forward and side scatter channels, and for doublets using both FSC-A vs FSC-H and SSC-A vs. SSC-H. Nuclei falling within the DAPI bright gate were collected into 0.5 ml of 1%BSA in PBS with RNAse inhibitor (0.5U/μl). Yields ranged from 6000 to 60,000 nuclei per sample. Following sorting, nuclei were centrifuged at 600 × *g* for 6 min and the pellet was resuspended and passed through a sterile 40 μm filter (Fisher # 08-771-23, Tewksbury, Massachusetts). Up to 20,000 nuclei, but less than 40 μl, were loaded for 10× Chromium library preparation.

### Library sequencing

Within our experiments, optimal sequence data generation resulted from loading 10,000–20,000 nuclei for 10× Chromium Single cell 3’ v3 library construction. All sequencing was performed on an Illumina Novaseq 6000 S2 2 × 50, according to manufacturer guidelines (10x Genomics, Doc #CG000204). The target read depth was 40,000 reads per nuclei library. Post sequencing, library quality was assessed by Cellranger 3.0.2 (10X Genomics, (https://github.com/10XGenomics/cellranger)) count output.

### Statistics and reproducibility

Raw sequence data for all 11 mice and 5 human samples were processed using Cellranger 3.0.2 Mkfastq (formation of fastq files) and Count to generate gene expression matrices aligned to custom per-mRNA mm10 (mouse samples) or hg19 (human samples) reference genomes. Intronic reads were included in the generating count matrix with Cellranger. QC metrics for this study were comparable to prior studies of skeletal muscle nuclei. roughly in the same QC level as other muscle snRNAseq studies^18^.

Doublets, identified using DoubletFinder_V3^23^, along with any nuclei <200umi were removed along with mitochondrial genes before aggregating datasets. Major cell-type populations were identified through T-SNE dimensional reduction along with k-means clustering (K = 10). Each of these populations was reanalyzed using Cellranger to identify specific cell types.

Statistical analysis of differential gene expression was performed using Seurat 3.2.3 (https://satijalab.org/seurat/) run on R version 3.3.2. Single nuclei data were logged normalized and scaled. Within each subcluster, marker genes were identified for each cell type using DESeq2 (Wald test on normalized gene expression) using FindAllMarkers om Seurat. Exact *p*-values are listed for all marker genes before and after Bonferroni correction. For each identified cell type, we also compared the *mdx* vs WT nuclei, as well as *mdx* vs e23AON-treated *mdx* by gene expression using DESeq2 test using FindAllMarkers. Gene ontology enrichment analyses were performed using Ease David, a complete list of *p*-values, and multiple testing corrections listed in Supplementary Data 4, 5, and 6. To determine the relatedness of highly similar cell types, we used the Pseudotime analysis R package Slingshot 1.7.2^27^. The remaining clusters underwent UMAP clustering, (Seurat Run UMAP based on the Seurat FindVariableFeatures) for use in Slingshot analysis. The R package Harmony was used to correct data to ensure distinct cell types were not the product of batch effects^73^.

Kmers of length 25 were extracted from mouse *Dmd* sequence NM_007868.6 matching reads that aligned to junctions e22–e23, e23–e24, and e22–e24. FASTQ reads where then pattern matched against the kmers using the Linux grep utility. Any read matching a kmer of interest was retained and counted. 10× barcodes were used to correct PCR duplicates, and unique reads were counted.

*P*-values for expansions and contractions of cell types between WT and *mdx* were calculated using a Mann–Whitney U test (nonparametric test for small samples sizes), and groups were WT (*n* = 3) frequency percentages vs. total *mdx* (*n* = 8) frequency percentages.

### Human subjects

Informed consent was obtained for all human subjects in compliance with institutional and federal laws, and UCLA IRB approvals 18-001366 or 19-00090.

## Supplementary information

Supplementary Information

Description of Additional Supplementary Files

Supplementary Data 1

Supplementary Data 2

Supplementary Data 3

Supplementary Data 4

Supplementary Data 5

Supplementary Data 6

Supplementary Data 7

Supplementary Data 8

Supplementary Data 9

Supplementary Data 10

nr-reporting-summary

## Data Availability

All data associated with this study are available in the main text or the supplementary materials. Source data underlying Fig. 6a and Fig. 7c are presented in Supplementary Data 7–8, respectively. All data have been deposited in the NCBI Sequence Read Archive, accession numbers: PRJNA771932 (mouse), PRJNA772047 (human).
