# Supplementary material for: Single nuclei transcriptomics of muscle reveals intra-muscular cell dynamics linked to dystrophin loss and rescue
Source: Commun Biol. 2022 Sep 19;5:989. doi: 10.1038/s42003-022-03938-0 (PMC9485160; doi:10.1038/s42003-022-03938-0)
Supplement: Supplementary file 1 — Supplementary Information [file 42003_2022_3938_MOESM1_ESM.pdf]

Supplementary Materials:
Supplementary References 1-204

Supplementary Fig. 1: Heat maps mouse: Smooth muscle subclusters
Supplementary Fig. 2: Heat maps mouse: Endothelial subclusters
Supplementary Fig. 3. Lineage tracing analysis of subpopulations identifies functional relatedness of endothelial and smooth muscle sub populations
Supplementary Fig. 4: Heat maps mouse: Myofiber subclusters
Supplementary Fig. 5: Heat maps mouse: Myoprogenitor subclusters
Supplementary Fig. 6. Exon skipping events are mapped to specific nuclei in e23AON-treated mdx
Supplementary Fig. 7. Dystrophin staining MOUSE 25 – mdx
Supplementary Fig. 8. Dystrophin staining MOUSE 73 – mdx
Supplementary Fig. 9. Dystrophin staining MOUSE 128 - mdx
Supplementary Fig. 10. Dystrophin staining MOUSE 155 – mdx
Supplementary Fig. 11. Dystrophin staining MOUSE 43 - mdx e23AON
Supplementary Fig. 12. Dystrophin staining MOUSE 45 - mdx e23AON
Supplementary Fig. 13. Dystrophin staining MOUSE 152 – mdx e23AON
Supplementary Fig. 14. Dystrophin staining MOUSE 169 – mdx e23AON
Supplementary Fig. 15. Dystrophin staining MOUSE c57 WT
Supplementary Fig. 16: Cell type identity based on expression of significantly expressed genes (additional celltypes)
Supplementary Fig. 17: Heat maps mouse: Immune cell subclusters
Supplementary Fig. 18: Biological processes and proportional shift in immune and fibroblast cell types
Supplementary Fig. 19: Heat maps mouse: Fibroblast subclusters
Supplementary Fig. 20. e23AON treatment in mdx shifts multiple distinct cell types towards more WT behavior (additional cell types) part 1
Supplementary Fig. 21. e23AON treatment in mdx shifts multiple distinct cell types towards more WT behavior (additional cell types) part 2
Supplementary Fig. 22: Frequencies of cell subsets in human muscle tissue
Supplementary Fig. 23: Heat maps human: Myofiber subclusters
Supplementary Fig. 24: Heat maps human: Fibroblast subclusters
Supplementary Fig. 25: Heat maps human: Endothelial cell subclusters
Supplementary Fig. 26: Heat maps human: Smooth muscle subclusters
Supplementary Fig. 27: Heat maps human: Macrophage subclusters

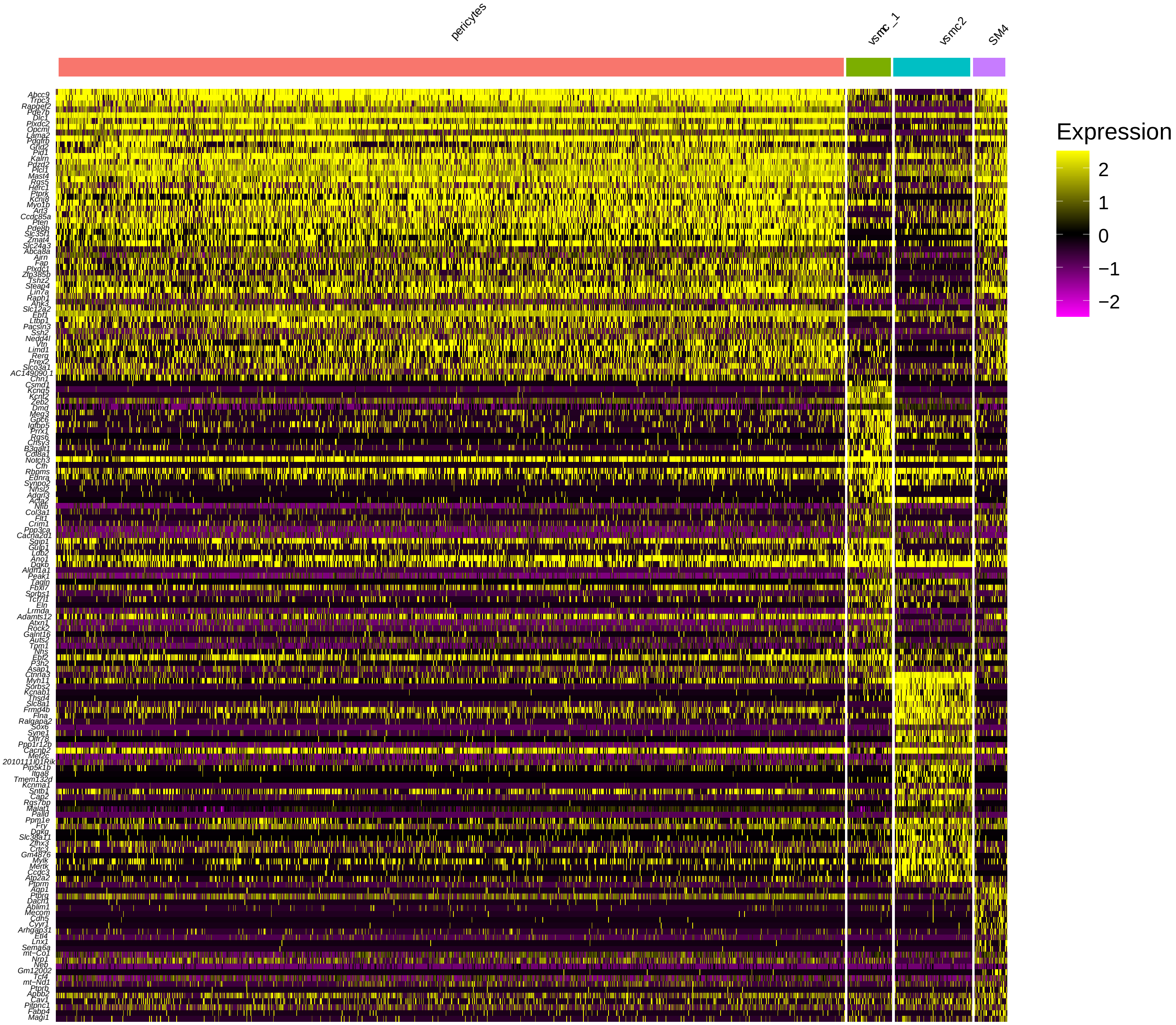

Supplementary Fig. 1: Heat maps mouse: Smooth muscle subclusters
Heatmap of significant marker genes among mouse Smooth muscle cell types. All significant marker genes (Supplementary Data 2) were used to generate these heatmaps, gene names listed on the beginning of each row. Each column represents an individual cell (grouped together by cell type identification). Color indicates normalized expression level as shown in legend).

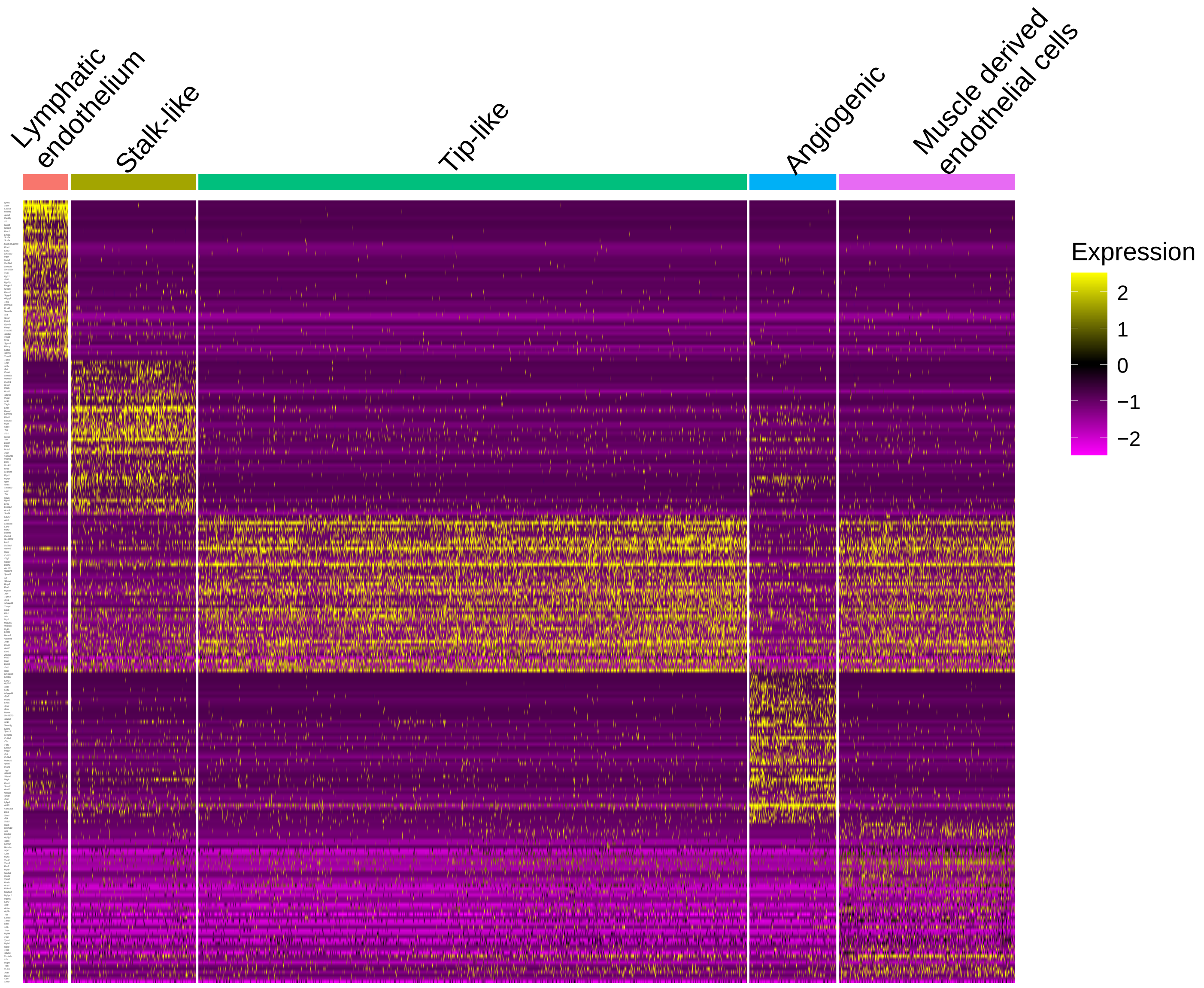

Supplementary Fig. 2: Heat maps mouse: Endothelial subclusters
Heatmap of significant marker genes among mouse Endothelial cell types. All significant marker genes (Supplementary Data 2) were used to generate these heatmaps, gene names listed on the beginning of each row. Each column represents an individual cell (grouped together by cell type identification). Color indicates normalized expression level as shown in legend).

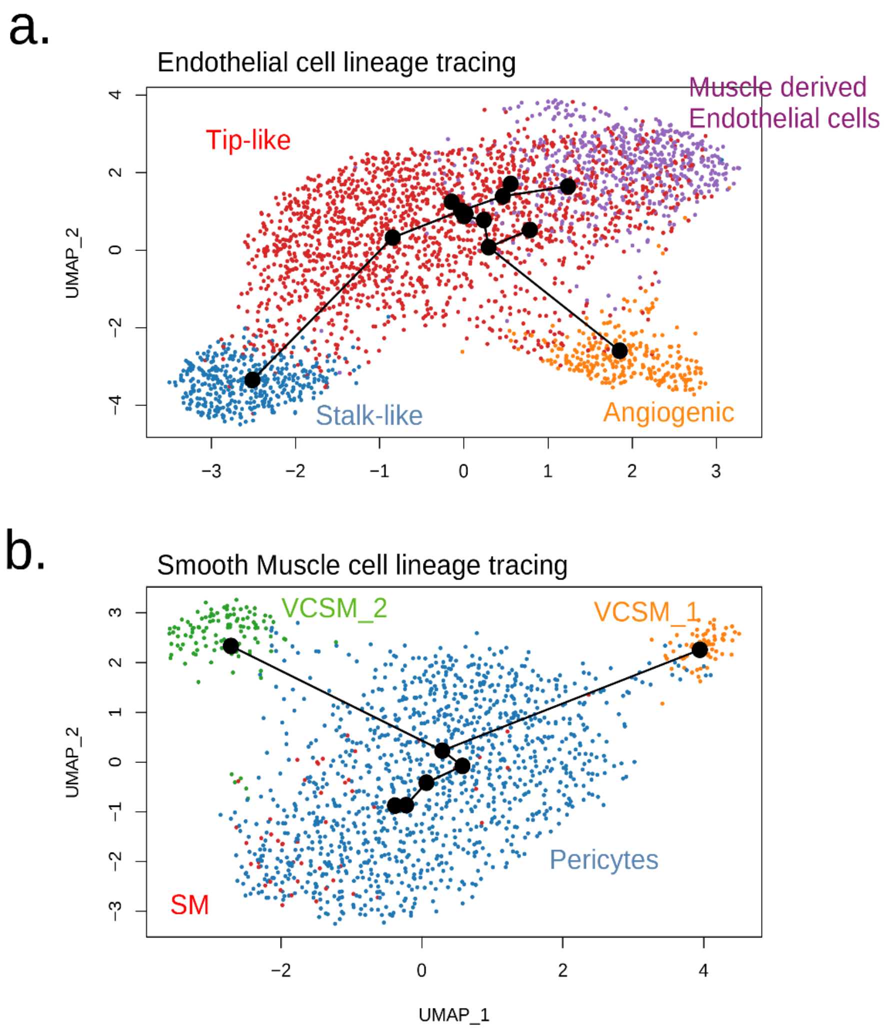

Supplementary Fig. 3. Lineage tracing analysis of subpopulations identifies functional relatedness of endothelial and smooth muscle sub populations.
Lineage tracing analysis of (**a**) Endothelial subpopulations, (**b**) Smooth muscle subpopulations. Subcluster populations are labeled with adjacent, color matched text.

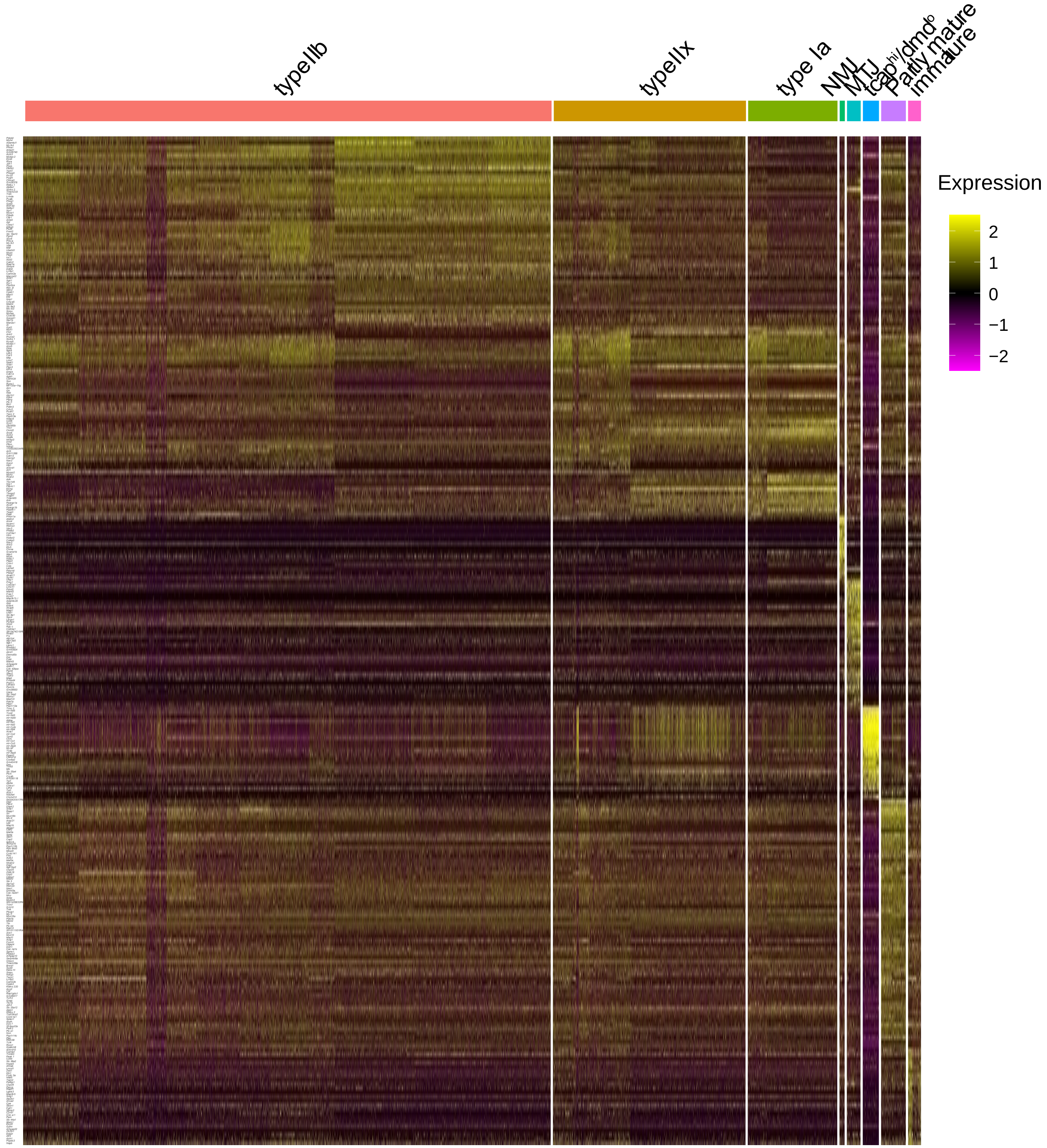

Supplementary Fig. 4: Heat maps mouse: Myofiber subclusters
Heatmap of significant marker genes among mouse myofiber cell types. All significant marker genes (Supplementary Data 2) were used to generate these heatmaps, gene names listed on the beginning of each row. Each column represents an individual cell (grouped together by cell type identification). Color indicates normalized expression level as shown in legend).

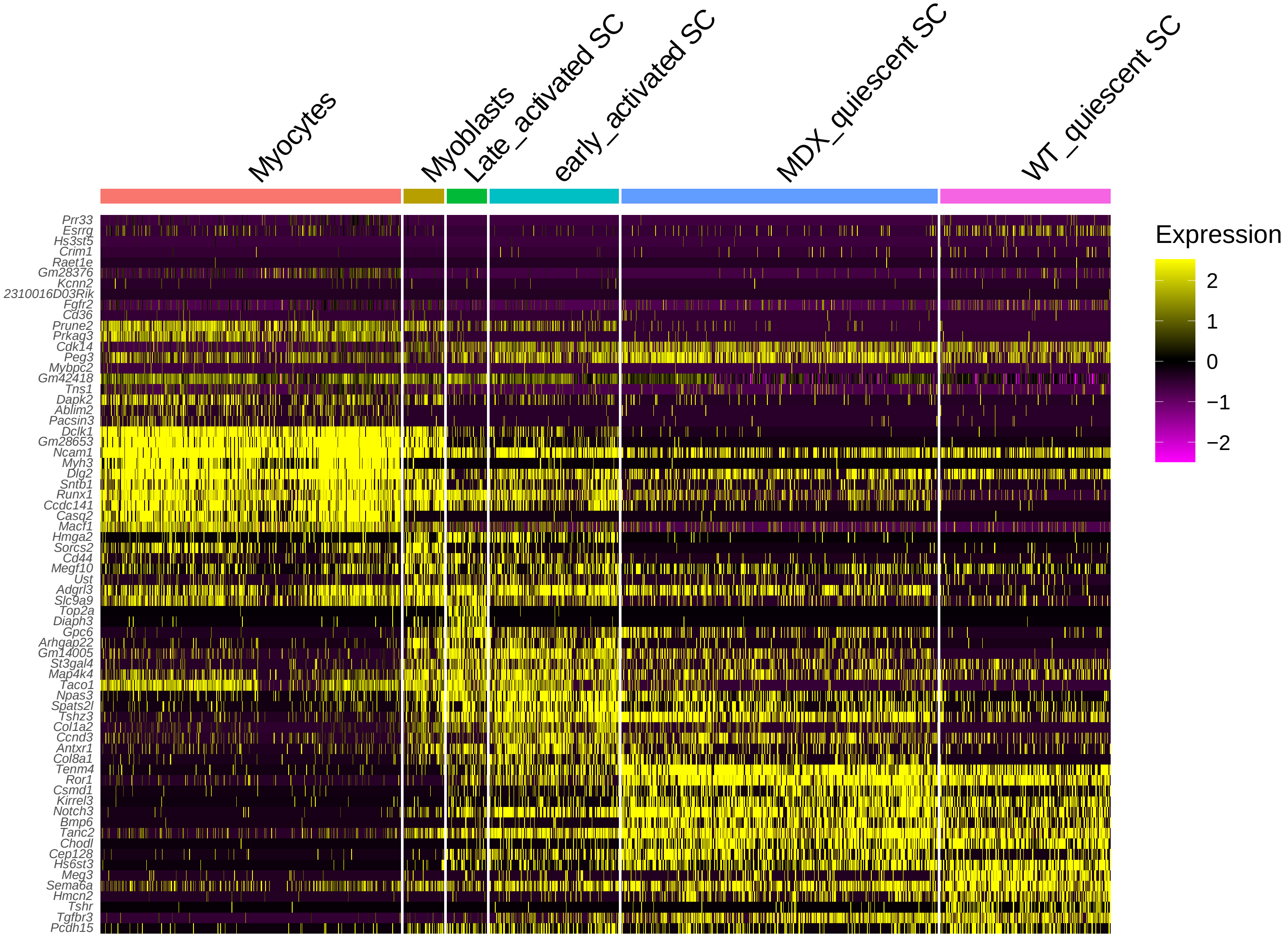

Supplementary Fig. 5: Heat maps mouse: Myoprogenitor subclusters
Heatmap of significant marker genes among mouse Myoprogenitor cell types. All significant marker genes (Supplementary Data 2) were used to generate these heatmaps, gene names listed on the beginning of each row. Each column represents an individual cell (grouped together by cell type identification). Color indicates normalized expression level as shown in legend).

a

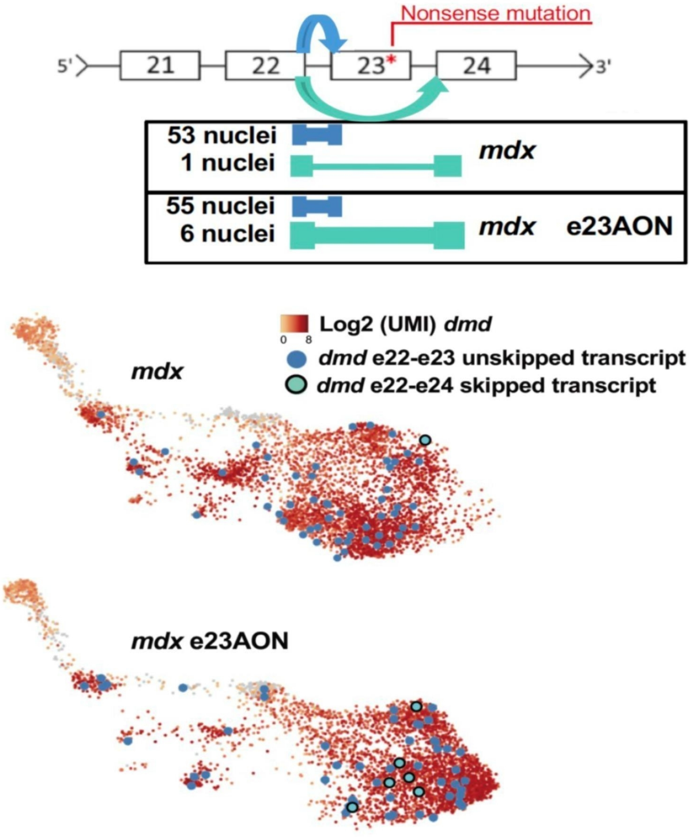

Supplementary Fig. 6 Exon skipping events are mapped to specific nuclei in e23AON-treated *mdx*

Diagram of *Dmd* expression and e22-e24 *Dmd* exon skipping. Number of nuclei containing *Dmd* e22-e24 (skipped light blue) and e22-e23 (unskipped dark blue) junction reads are shown to the left of the splicing events for each treatment condition. Nuclei where e22-e24 was observed are color coded on the UMAP of myolineage nuclei (from Fig. 3a) with overall *Dmd* abundance shown (Yellow-Red color scale).

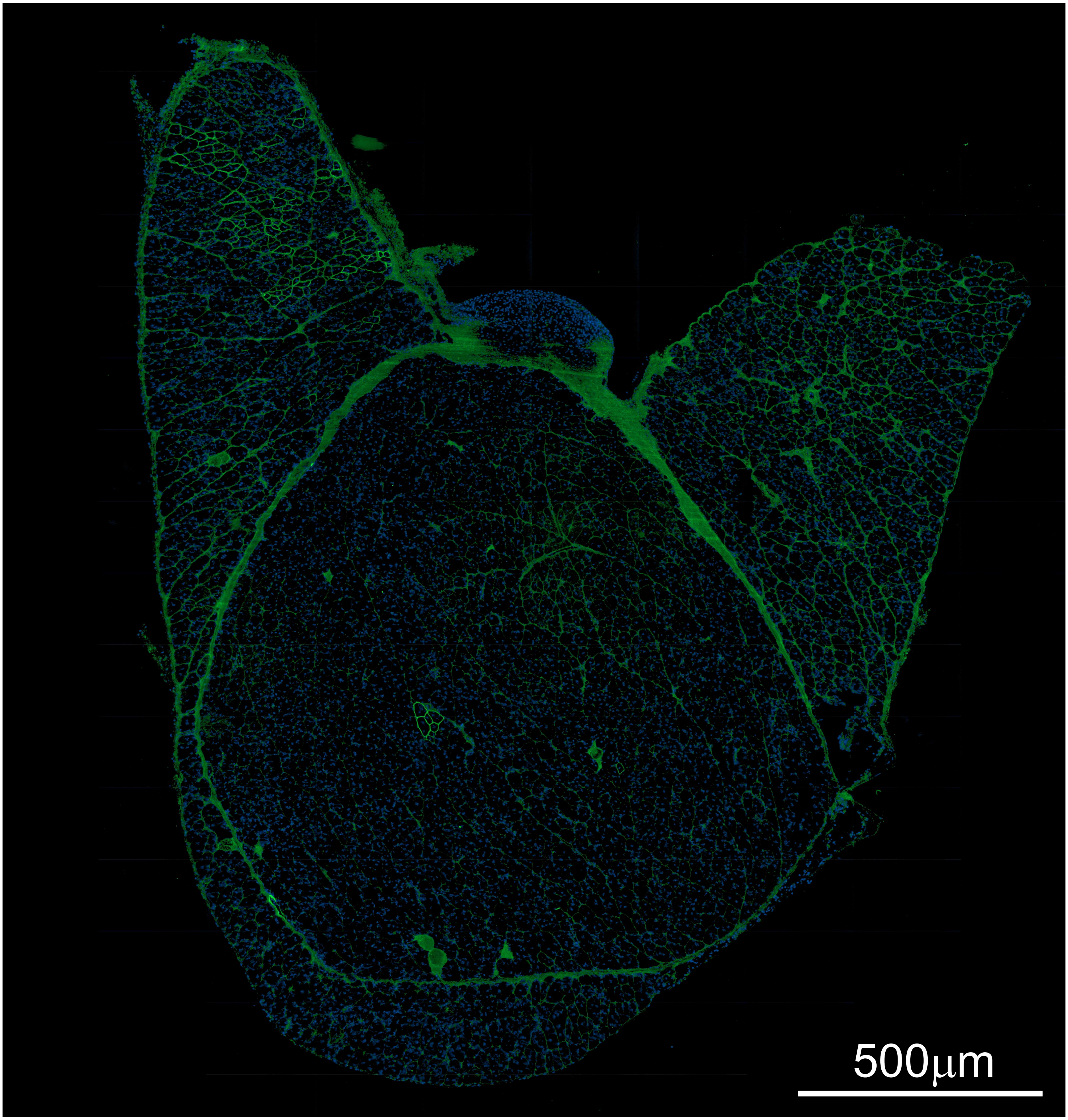

Supplementary Fig. 7. Dystrophin staining MOUSE 25 – *mdx*
Whole quadricep muscles from untreated *mdx* was frozen, cryosectioned and subjected to immunofluorescent staining for dystrophin protein (green). Scale bar = 500 μm

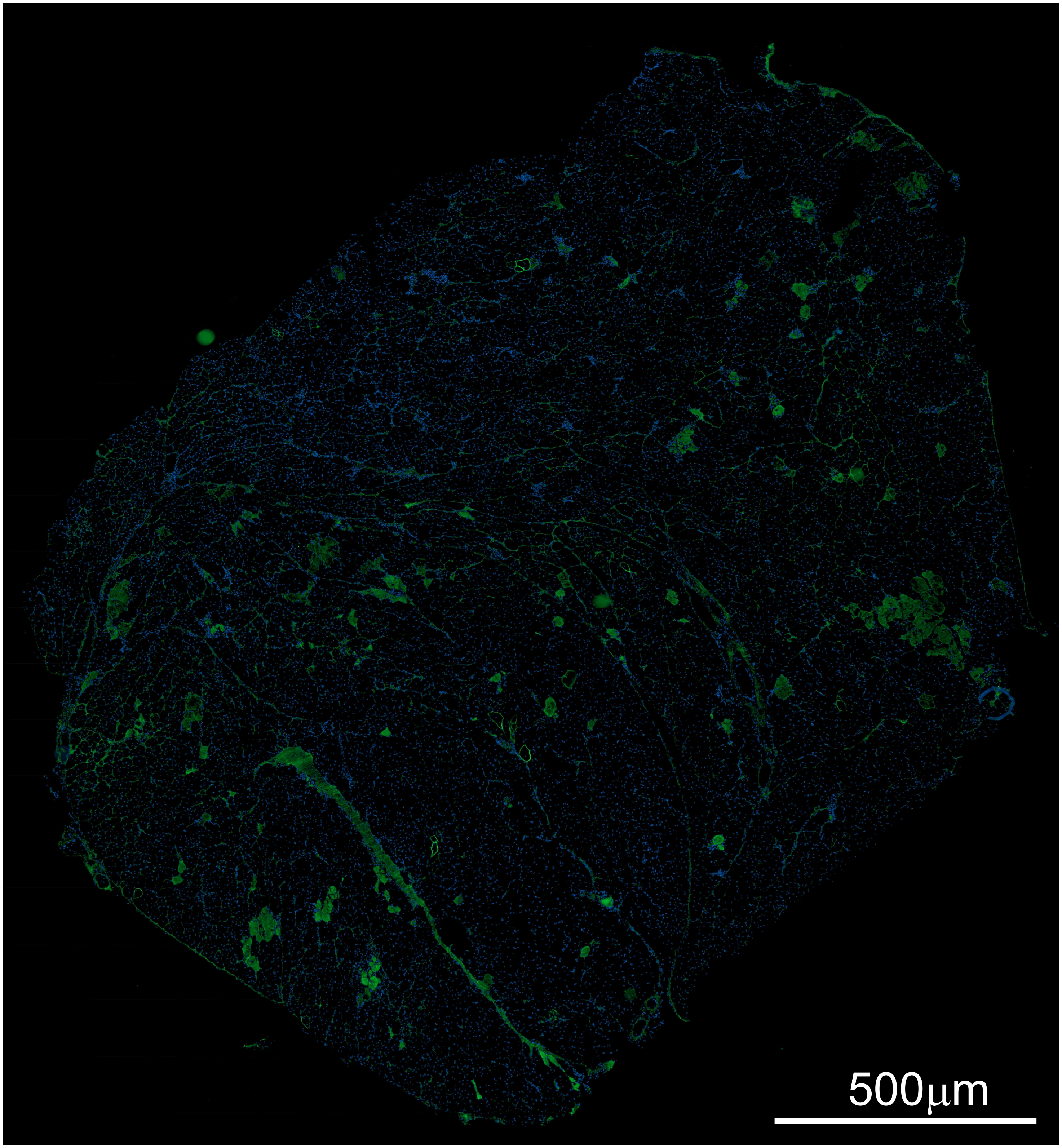

Supplementary Fig. 8. Dystrophin staining MOUSE 73 - *mdx*:
Whole quadricep muscles from untreated *mdx* was frozen, cryosectioned and subjected to immunofluorescent staining for dystrophin protein (green). Scale bar = 500μm

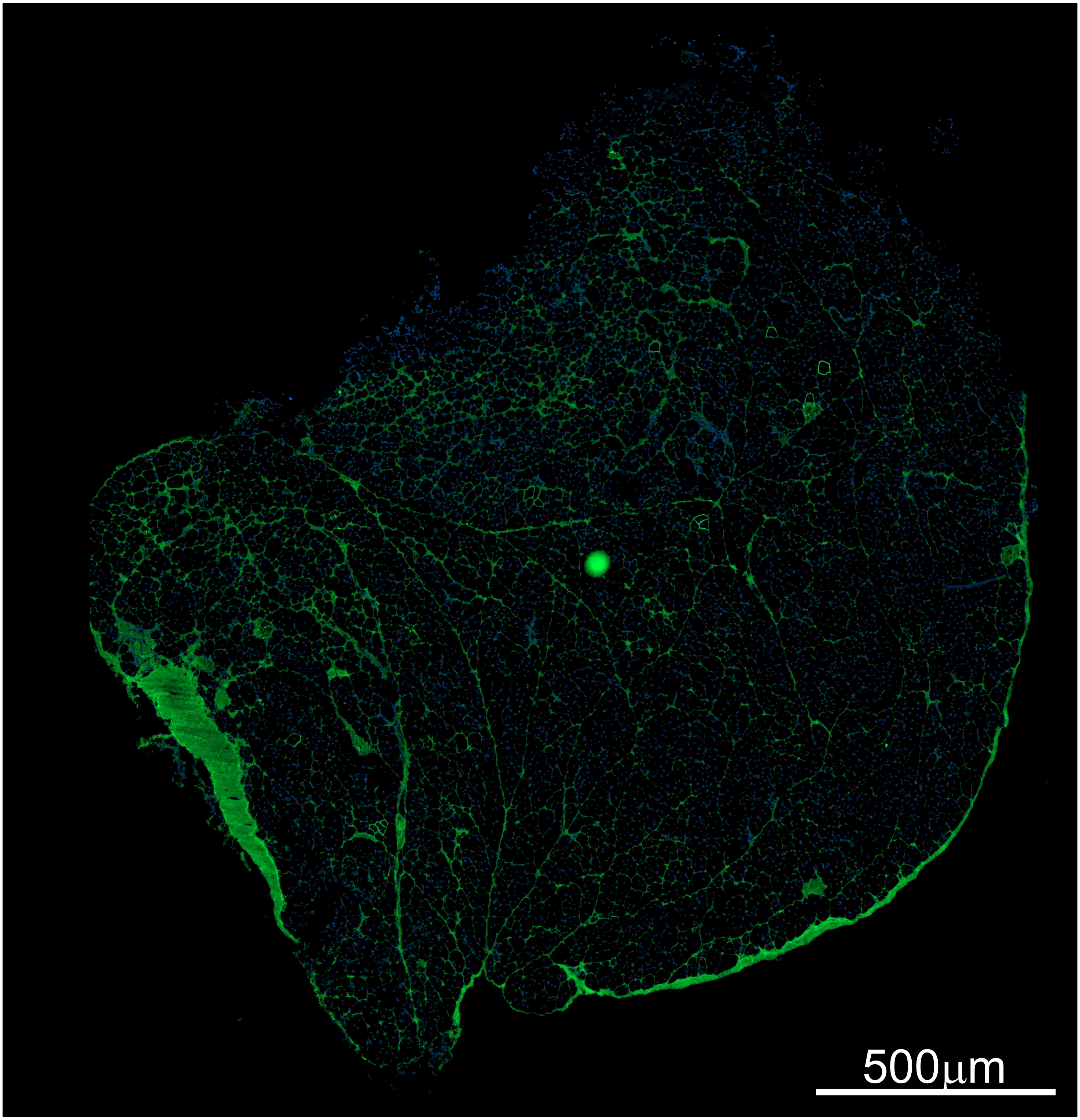

Supplementary Fig. 9. Dystrophin staining MOUSE 128 - *mdx*:
Whole quadricep muscles from untreated *mdx* was frozen, cryosectioned and subjected to immunofluorescent staining for dystrophin protein (green). Scale bar = 500 μm

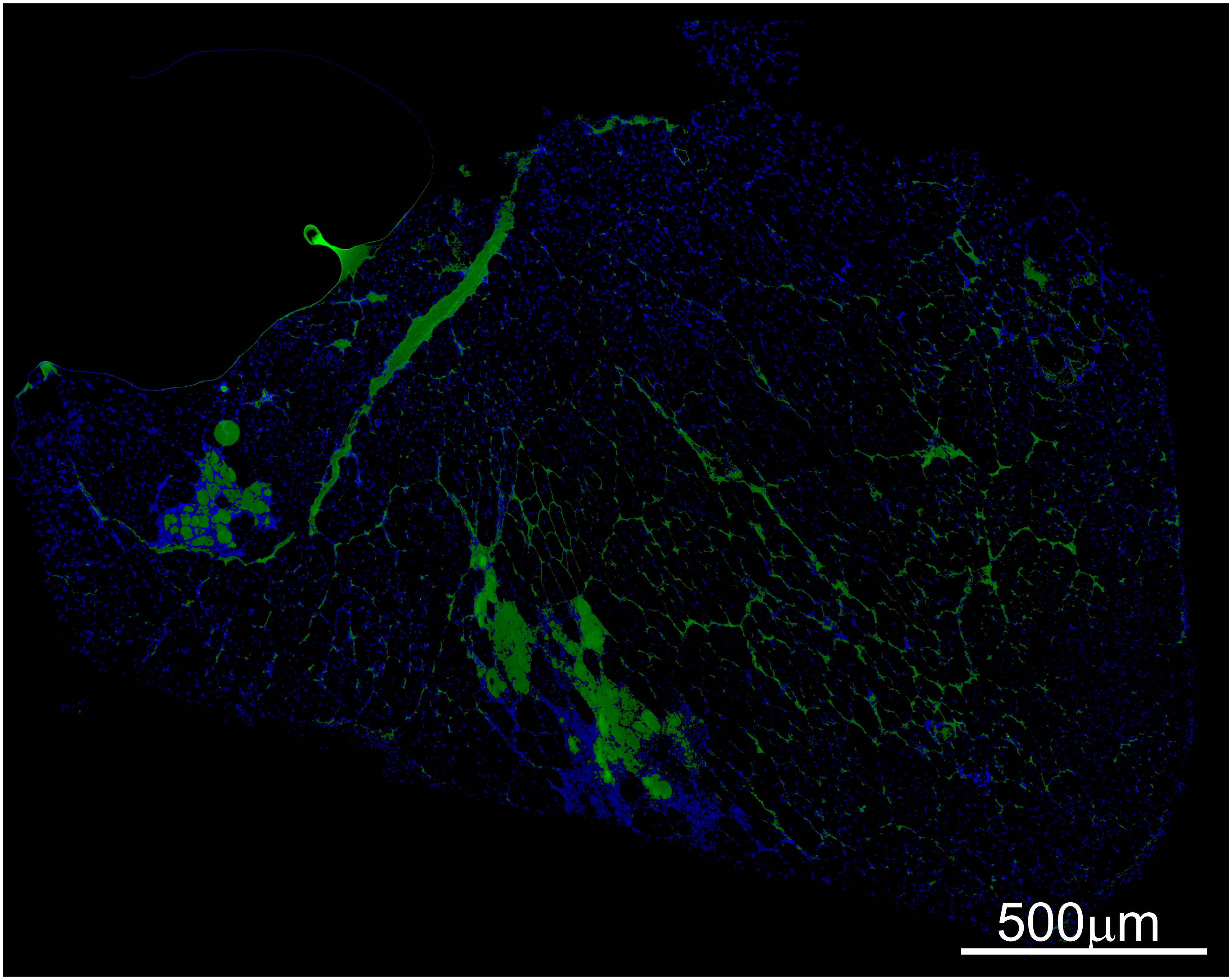

Supplementary Fig. 10. Dystrophin staining MOUSE 155 - *mdx*:
Whole quadricep muscles from untreated *mdx* was frozen, cryosectioned and subjected to immunofluorescent staining for dystrophin protein (green). Scale bar = 500µm

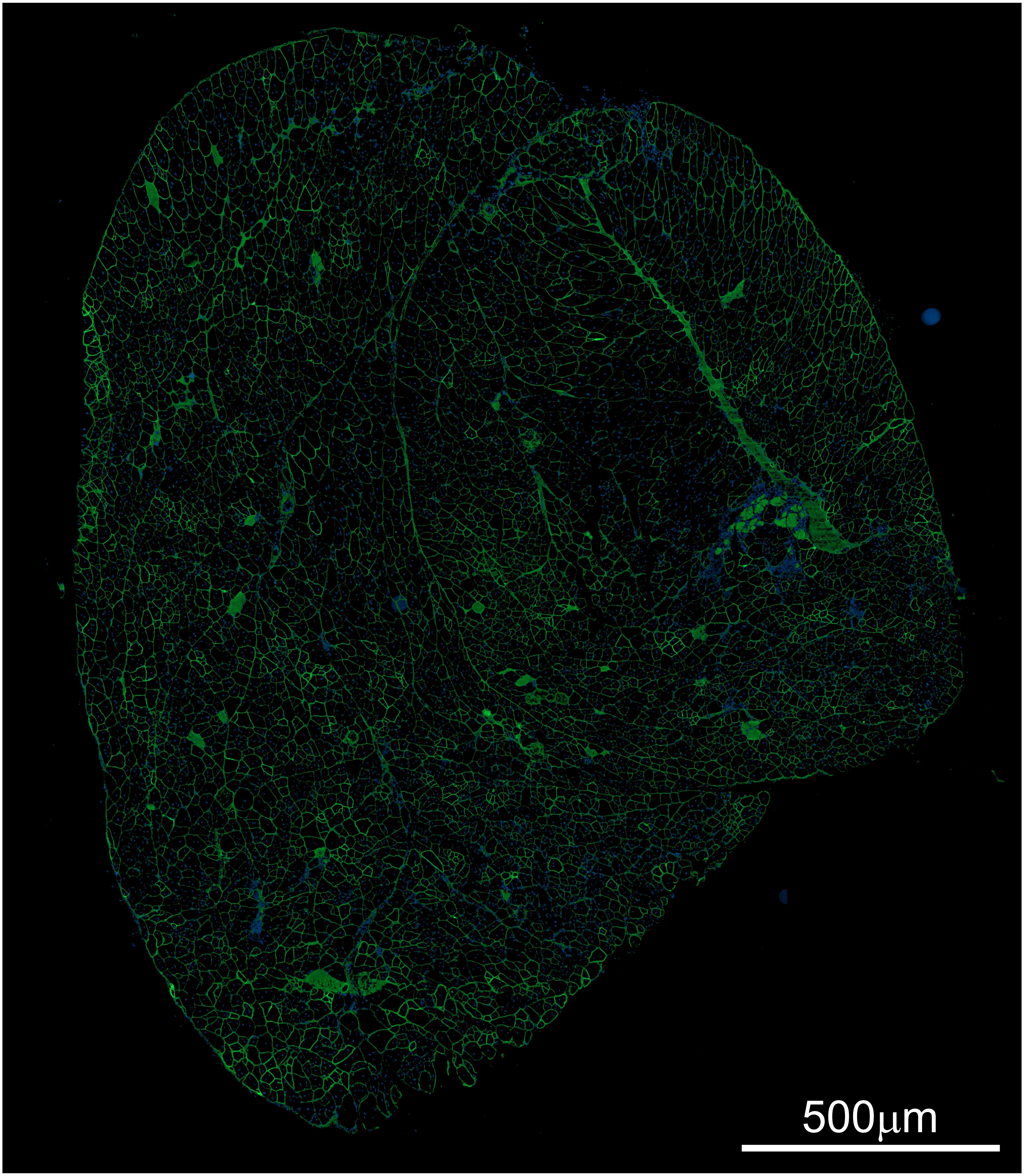

Supplementary Fig. 11. Dystrophin staining MOUSE 43 - *mdx* e23AON:
Whole quadricep muscles from e23AON treated *mdx* was frozen, cryosectioned and subjected to immunofluorescent staining for dystrophin protein (green). Scale bar = 500μm

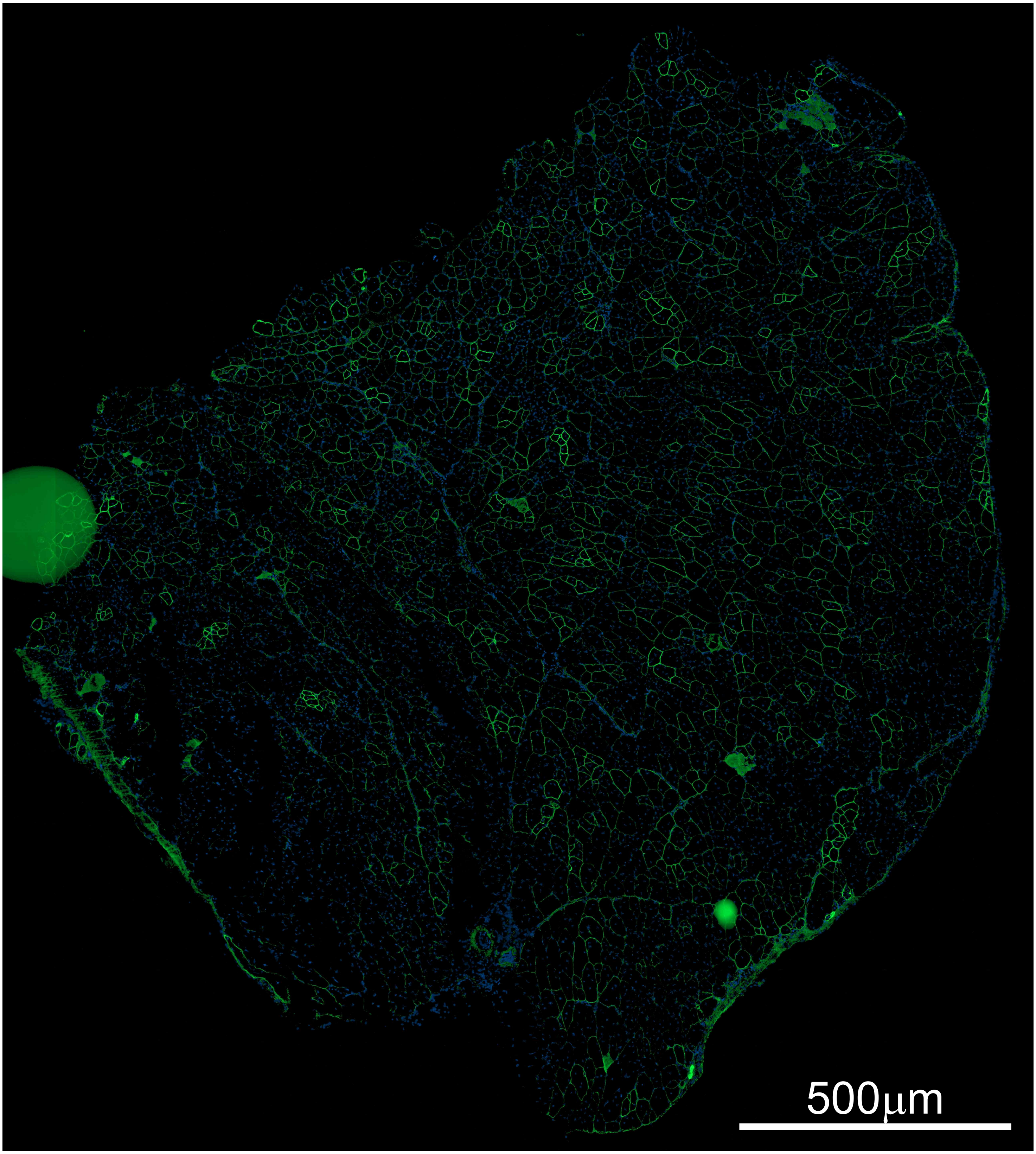

Supplementary Fig. 12. Dystrophin staining MOUSE 45 - *mdx* e23AON:
Whole quadricep muscles from e23AON treated *mdx* was frozen, cryosectioned and subjected to immunofluorescent staining for dystrophin protein (green). Scale bar = 500μm

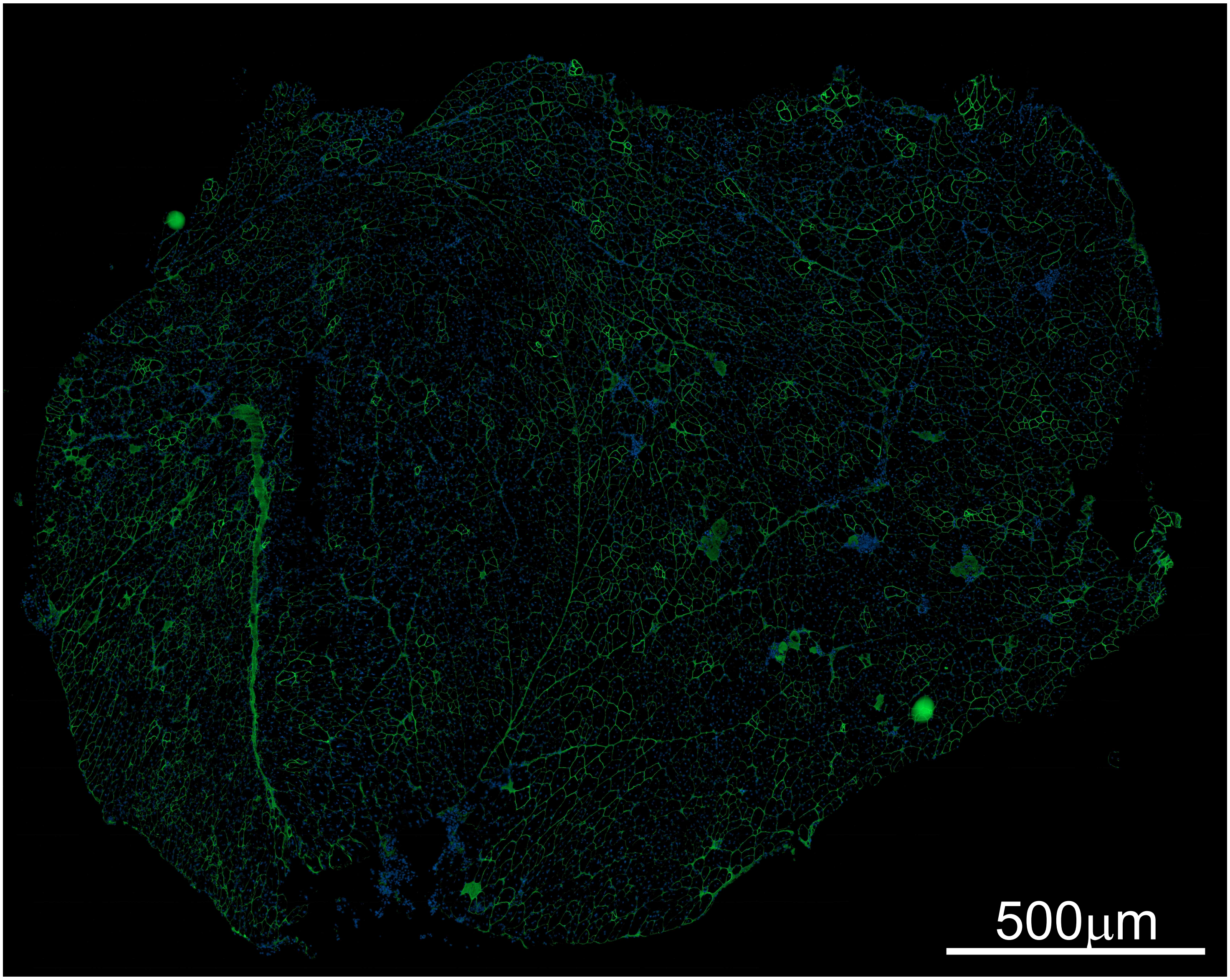

Supplementary Fig. 13. Dystrophin staining MOUSE 152 - *mdx* e23AON:
Whole quadricep muscles from e23AON treated *mdx* was frozen, cryosectioned and subjected to immunofluorescent staining for dystrophin protein (green). Scale bar = 500µm

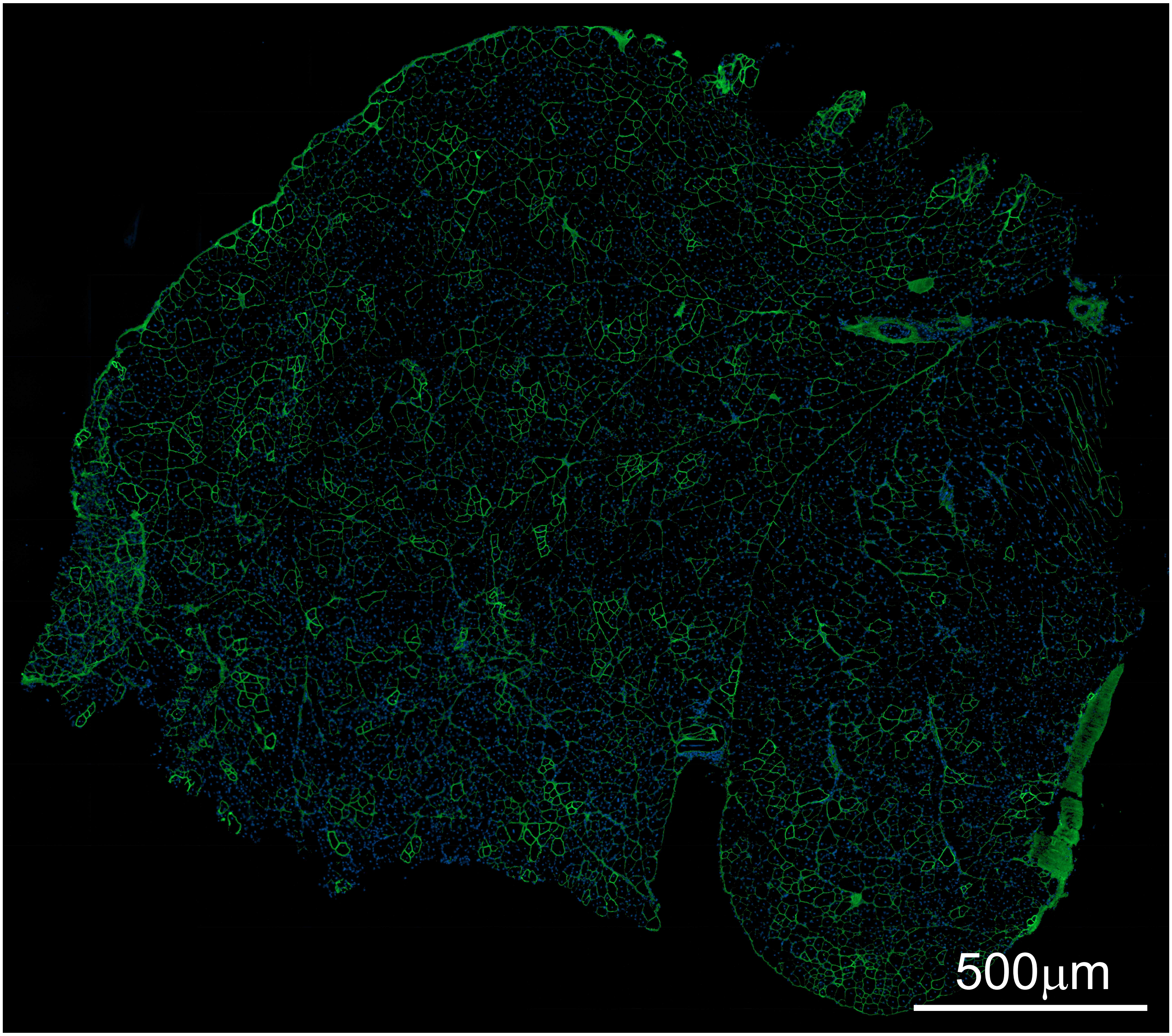

Supplementary Fig. 14. Dystrophin staining MOUSE 169 – *mdx* e23AON:
Whole quadricep muscles from e23AON treated *mdx* was frozen, cryosectioned and subjected to immunofluorescent staining for dystrophin protein (green). Scale bar = 500 μm

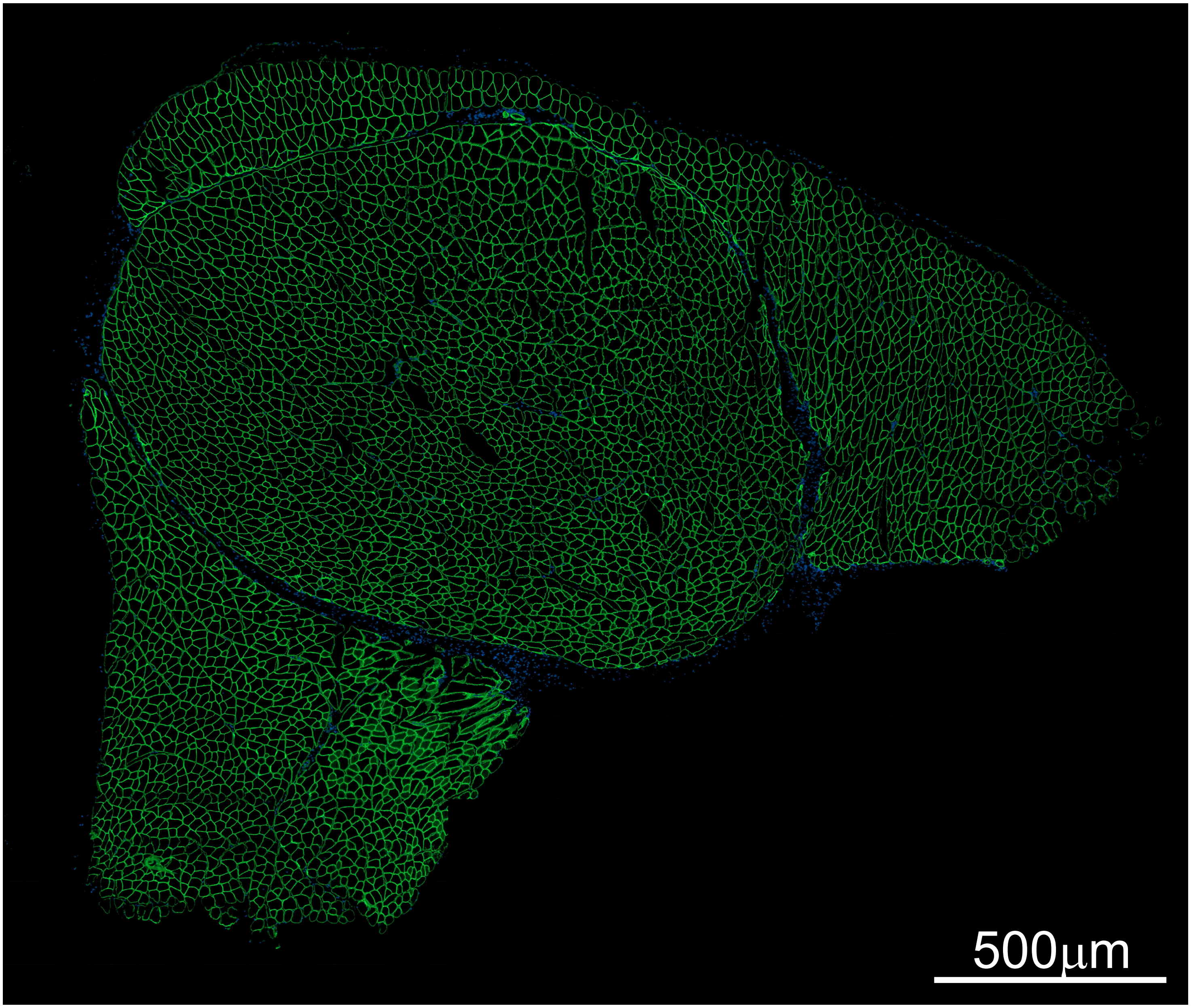

Supplementary Fig. 15. Dystrophin staining MOUSE c57 WT:
Whole quadricep muscles from c57 WT was frozen, cryosectioned and subjected to immunofluorescent staining for dystrophin protein (green). Scale bar = 500μm

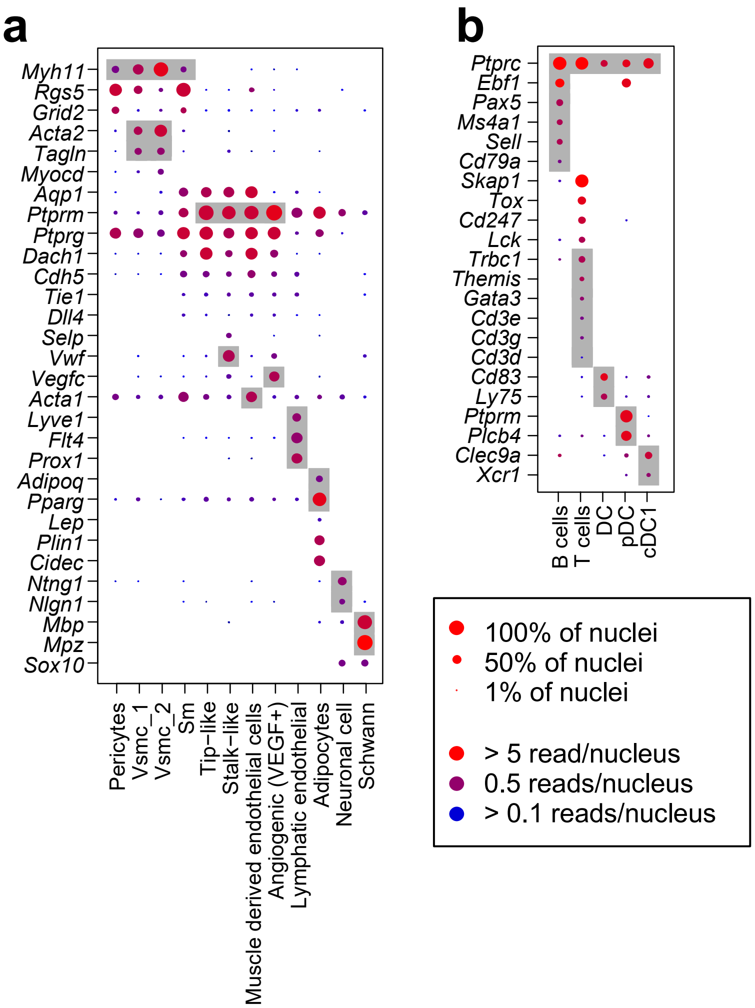

Supplementary Figure 16: Cell type identity based on expression of significantly expressed genes (additional cell types)

Gene expression differences between subtypes of major cell classes, not shown in other figures within the main text. (**a**) endothelial, smooth muscle, other. (**b**) non-macrophage immune cells, Subtype names indicated on x-axis, gene names indicated on y-axis. Log2 (Average gene expression) for each subtype shown by Blue-Red gradient. Percentage of cells expressing genes from 0% to 100% indicated by dot size. Gray background indicates known markers of the respective cell type on x-axis. Marker genes are listed in Supplementary Data 2, 3a.

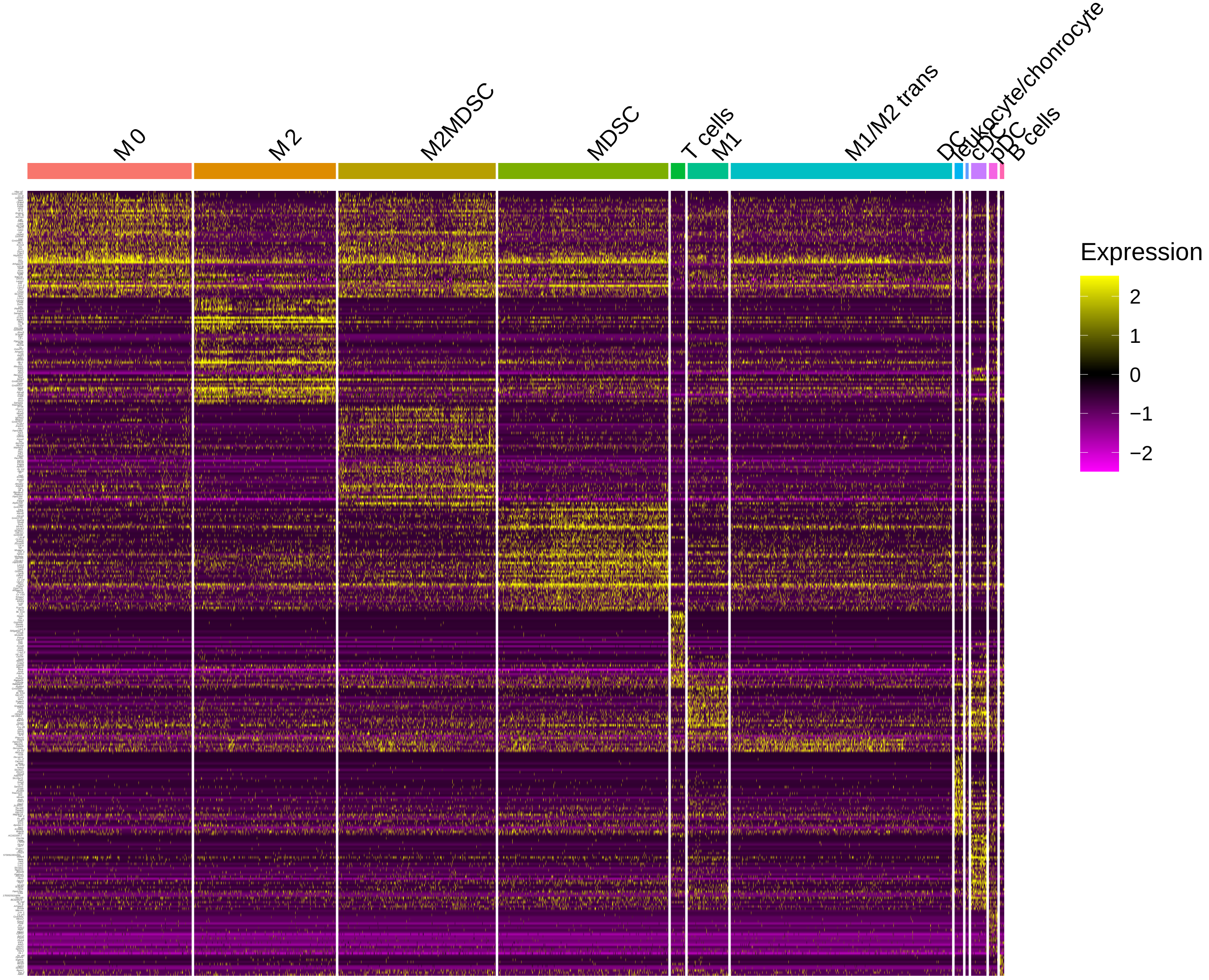

Supplementary Fig. 17: Heat maps mouse: Immune cell subclusters
Heatmap of significant marker genes among mouse Immune cell types. All significant marker genes (Supplementary Data 2) were used to generate these heatmaps, gene names listed on the beginning of each row. Each column represents an individual cell (grouped together by cell type identification). Color indicates normalized expression level as shown in legend).

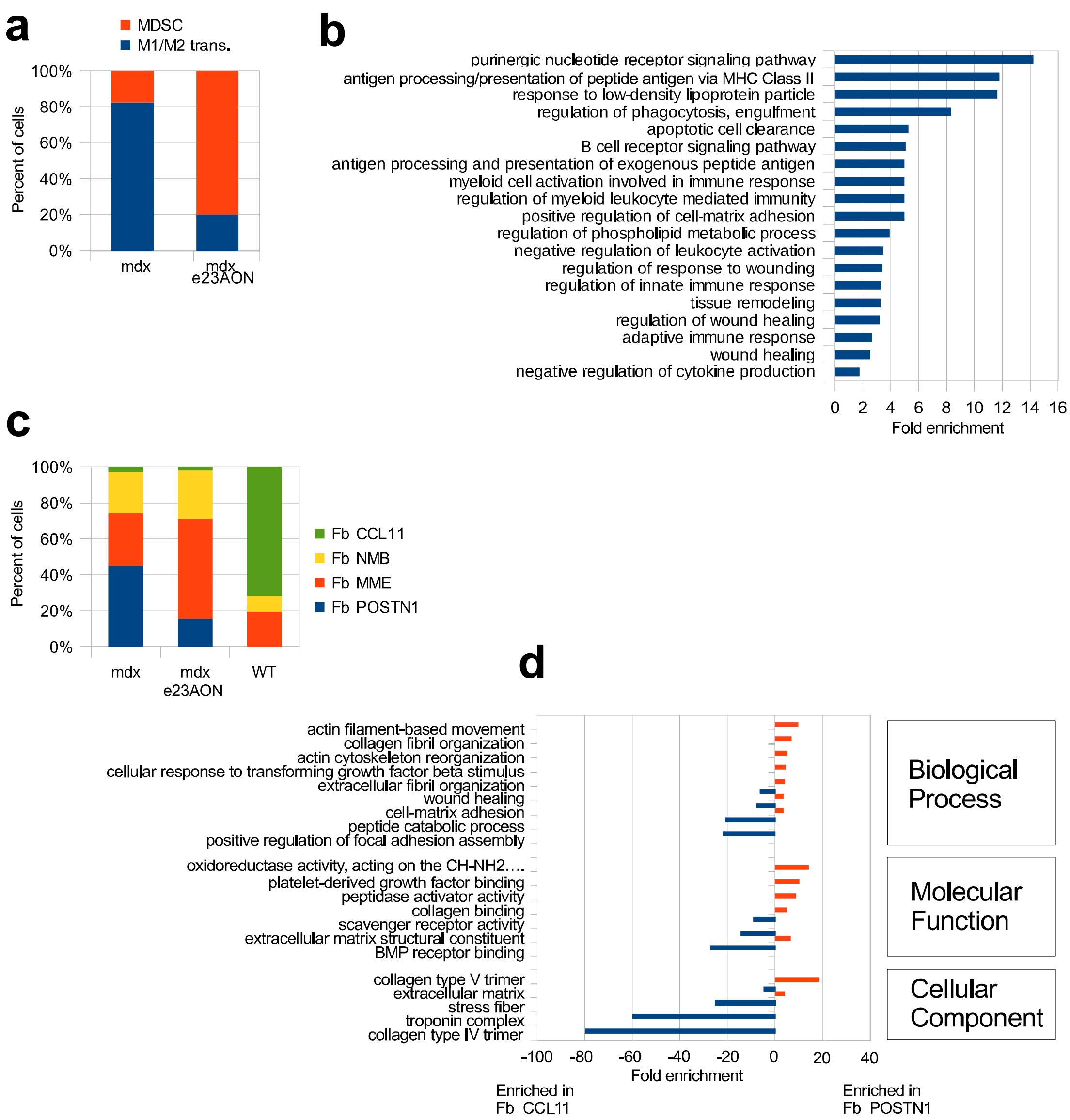

Supplementary Figure 18: Biological processes and proportional shift in immune and fibroblast cell types
(**a**) Depletion of M1/M2 trans and expansion of MDSC with e23AON treatment. Y-axis shows the relative percentage M1/M2 trans and MDSC populations in *mdx* and *mdx* e23AON. (**b**) Representative list of enriched GO terms (Y-axis) based on genes upregulated in MDSC relative to M1/M2 trans. Fold enrichment indicated on x-axis. List of DE Genes between MDSC and M1/M2 transitional cells listed in Supplementary Data 5. (**c**) Relative changes in cell proportions of Fb CCL11 and Fb POSTN1 populations and WT intermediates (Fb MME and Fb NMB) between *mdx*, *mdx* e23AON and WT conditions, Y-axis shows percentages of cell types (**d**) Representative list of enriched GO terms (Y-axis) based on genes differentially expressed between Fb CCL11 and Fb POSTN1. Fold enrichment indicated on x-axis, >0 shows GO terms enriched for genes upregulated in Fb POSTN1, <0 shows GO terms enriched for genes upregulated in Fb CCL11. List of DE Genes between Fb POSTN1 and Fb CCL11 listed in Supplementary Data 6.

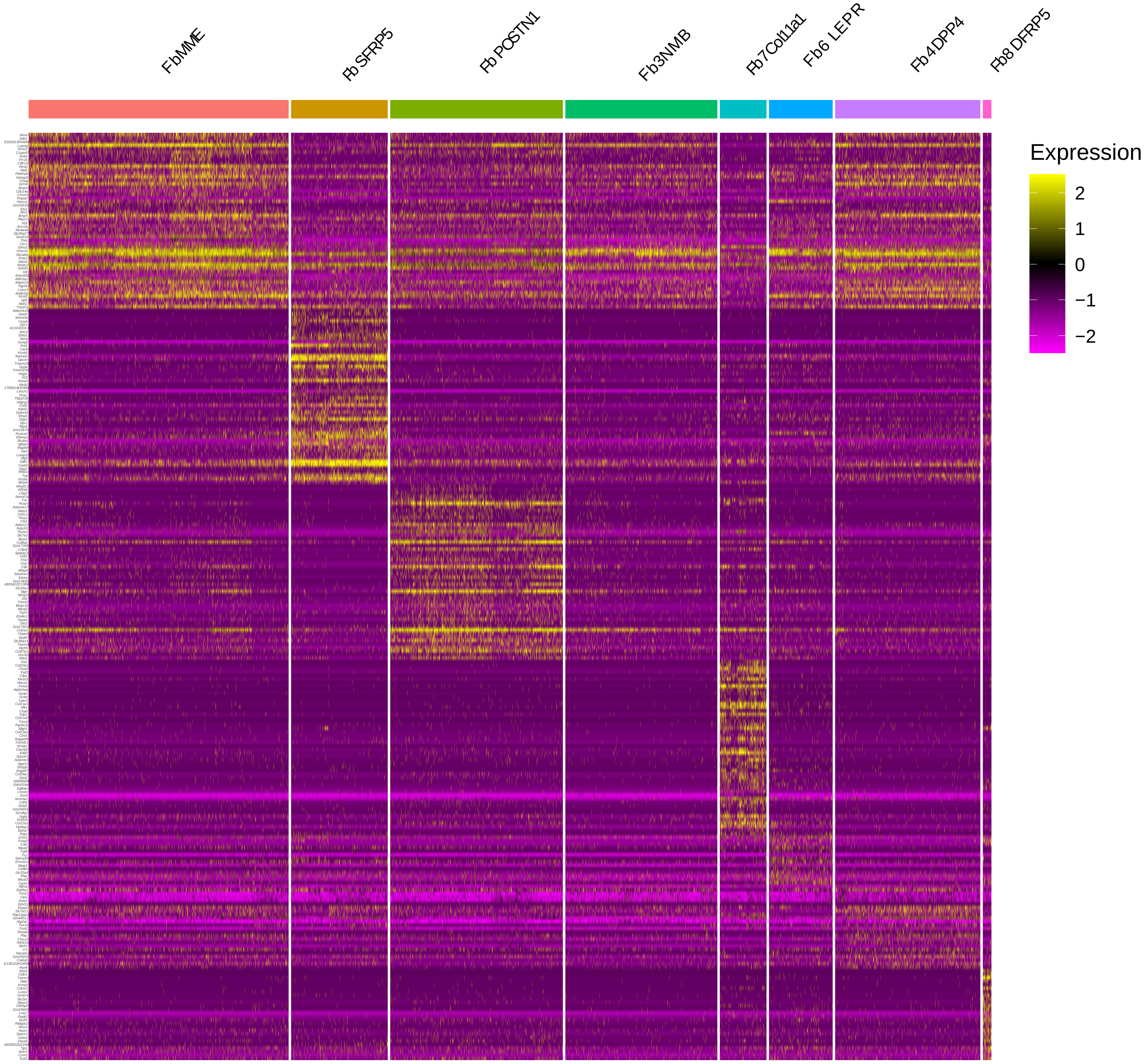

Supplementary Fig. 19: Heat maps mouse: Fibroblast subclusters
Heatmap of significant marker genes among mouse Fibroblast cell types. All significant marker genes (Supplementary Data 2) were used to generate these heatmaps, gene names listed on the beginning of each row. Each column represents an individual cell (grouped together by cell type identification). Color indicates normalized expression level as shown in legend).

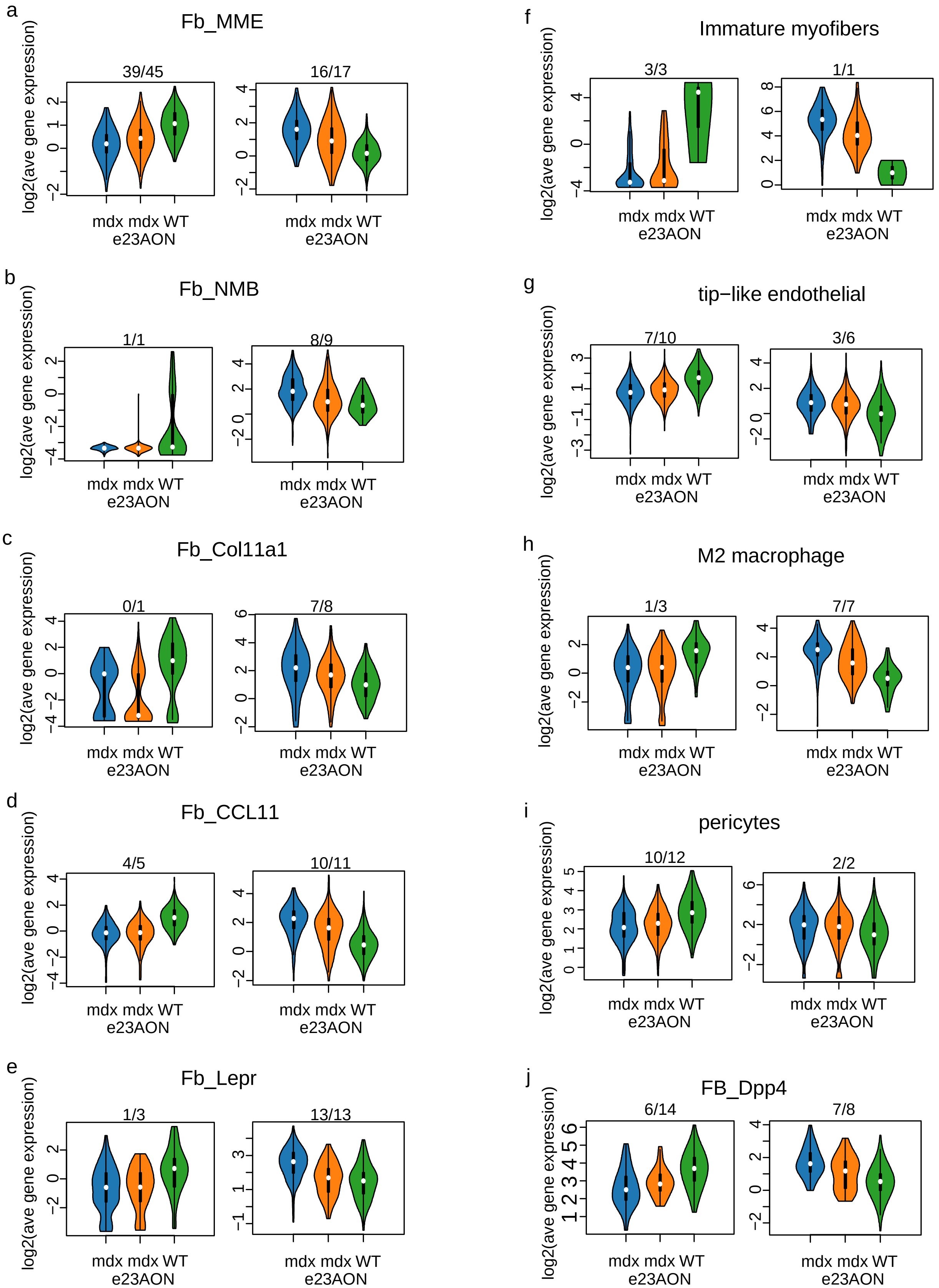

a
Fb_MME
39/45
16/17
b
Fb_NMB
1/1
8/9
c
Fb_Col11a1
0/1
7/8
d
Fb_CCL11
4/5
10/11
e
Fb_Lepr
1/3
13/13
f
Immature myofibers
3/3
1/1
g
tip−like endothelial
7/10
3/6
h
M2 macrophage
1/3
7/7
i
pericytes
10/12
2/2
j
FB_Dpp4
6/14
7/8
log2(ave gene expression)
mdx mdx WT
e23AON

Supplementary Fig. 20. e23AON treatment in *mdx* shifts multiple distinct cell types towards more WT behavior (additional cell types) part 1
Average expression of genes upregulated or downregulated in WT relative to untreated *mdx*. Cell type names are listed above plot. Y axes = log2average UMI for differentially expressed genes. White dot represents mean, box = q1 to q3, thin line represents min and max values. List of Genes between WT and *mdx* are listed in Supplementary Data 1.

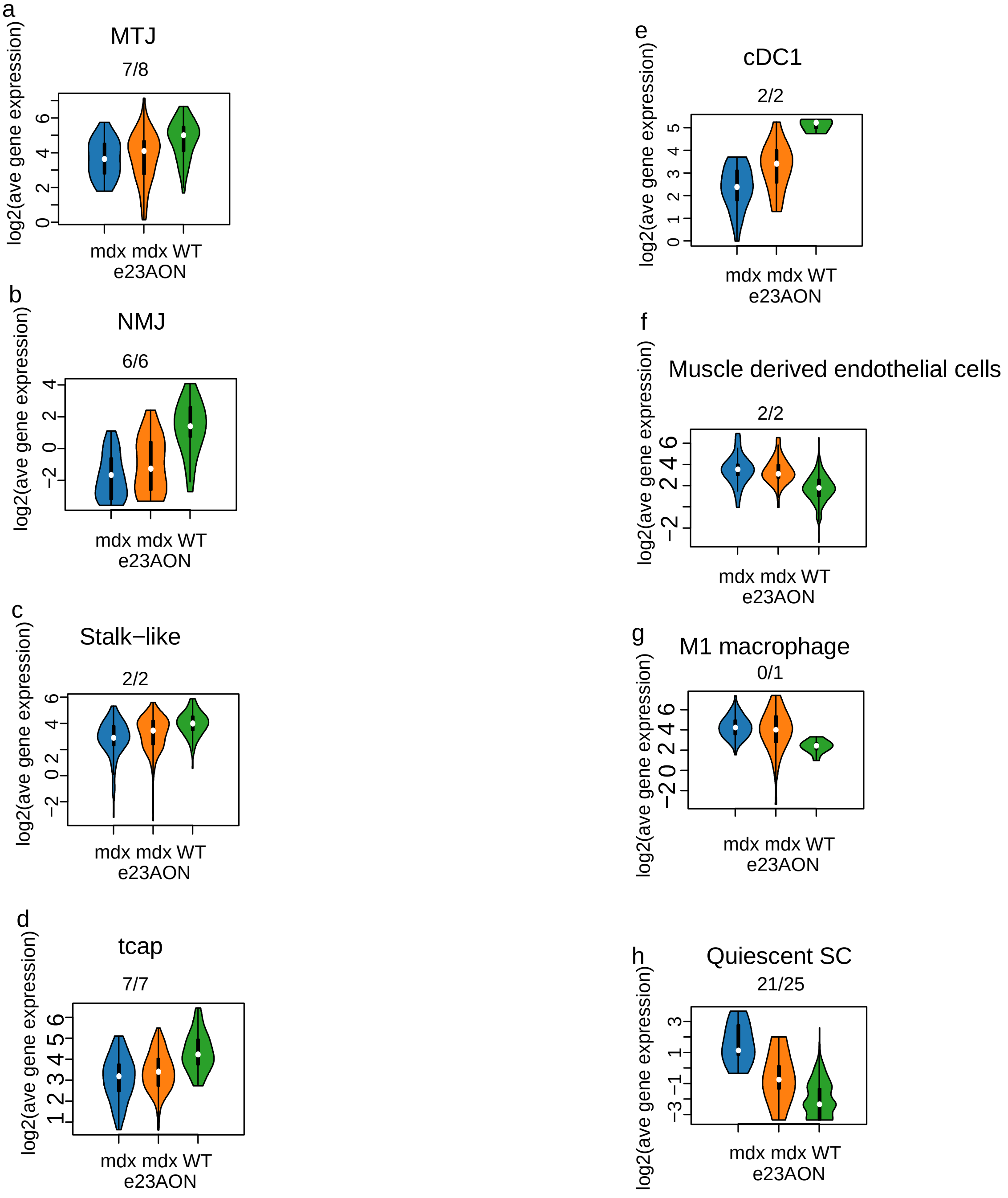

Supplementary Fig. 21. e23AON treatment in *mdx* shifts multiple distinct cell types towards more WT behavior (additional cell types) part 2
Average expression of genes upregulated or downregulated in WT relative to untreated *mdx*. Cell type names are listed above plot. Y axes = log2average UMI for differentially expressed genes. (**a** - **d**) no significant genes were detected to be down regulated in WT. (**e** – **h**) no significant genes were detected to be up regulated in WT. White dot represents mean, box = q1 to q3, thin line represents min and max values. List of Genes between WT and *mdx* are listed in Supplementary Data 1.

Major cell types	Healthy	*DMD*
Adipocytes	0.12	1.66
Myoblasts	0.44	4.55
Satellite cells	2.19	12.83
Immunne - Macrophages	0.96	5.35
Fibroblasts	6.26	17.35
Immune - Tcells and B cells	0.76	1.86
Endothelial cells	5.90	13.11
Myofibers	79.86	45.16
Smooth muscle cells	3.83	2.77
Immune - Mast cells	0.12	0.24

Myofibers	Healthy	*DMD*
Type I fibers	12.68	15.76
Type II-Tcap myonuclei	11.25	2.78
Type IIa fibers	26.71	11.74
Type IIx fibers	20.01	6.54
Type I-Tcap myonuclei	6.18	3.13

Smooth muscle cells	Healthy	*DMD*
Vsmc 1	0.12	0.70
Pericytes 1	1.04	1.29
Pericytes 2	2.03	0.26
Vsmc 2	0.60	0.52

Macrophages	Healthy	*DMD*
Mac 4	0.08	0.87
Mac 1	0.24	2.51
Mac 2	0.28	1.07
Mac 3	0.36	0.81

Fibroblasts/ FAPS	Healthy	*DMD*
Fb 1	0.00	5.90
Fb 2	0.36	5.31
Fb 4	0.44	2.58
Fb 5	1.72	0.90
Fb 3	3.75	2.67

Endothelial cells	Healthy	*DMD*
Stalk-like	1.24	5.22
Angiogenic	1.00	1.90
Tip-like	3.67	5.25

Supplementary Figure 22: Frequencies of cell subsets in human muscle tissue
Frequencies of cell populations identified in human healthy and DMD muscle. Percentages for each cell population named in the 1st column for healthy individuals and in the 2nd column for DMD affected individuals. Following the major cell types, cell types are further broken down to their respective subclusters. Yellow = increase and Blue = decrease in DMD percentage relative to healthy. Subsets within categories are listed in order of decreasing magnitude of difference between DMD and healthy.

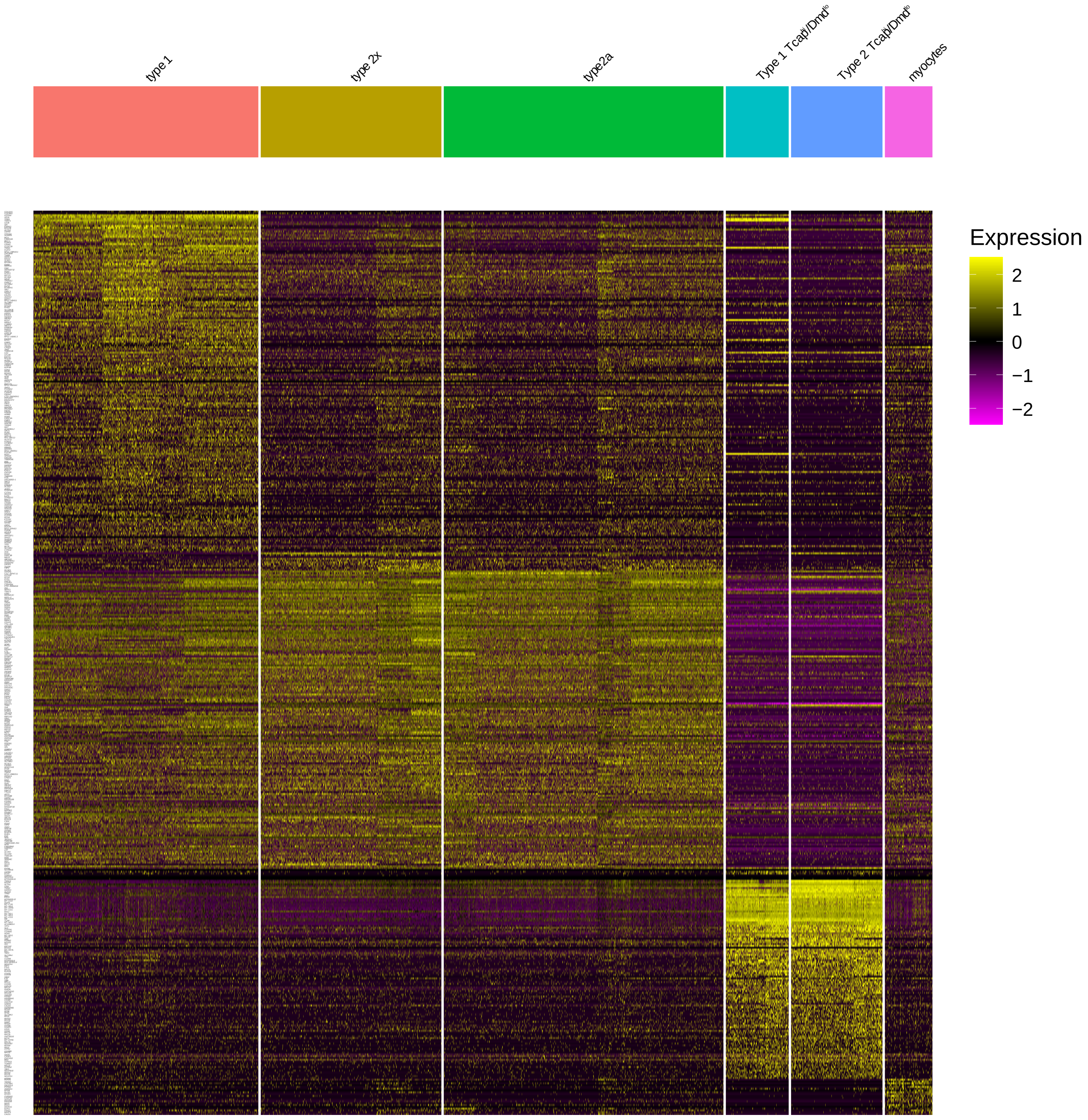

Supplementary Fig. 23: Heat maps human: Myofiber subclusters
Heatmap of significant marker genes among human myofiber cell types. All significant marker genes (Supplementary Data 9) were used to generate these heatmaps, gene names listed on the beginning of each row. Each column represents an individual cell (grouped together by cell type identification). Color indicates normalized expression level as shown in legend).

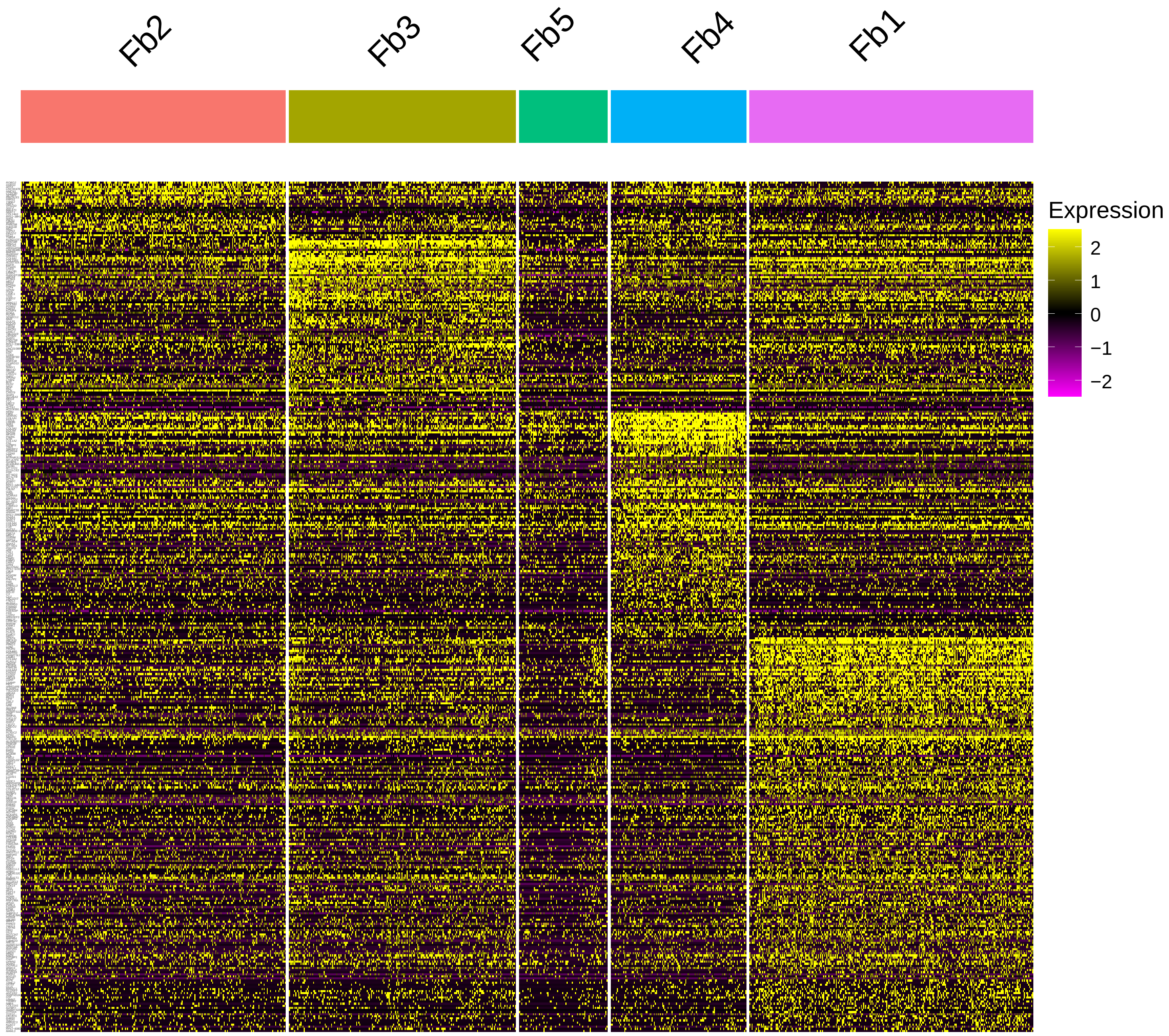

Supplementary Fig. 24: Heat maps human: Fibroblast subclusters
Heatmap of significant marker genes among human Fibroblast cell types. All significant marker genes (Supplementary Data 9) were used to generate these heatmaps, gene names listed on the beginning of each row. Each column represents an individual cell (grouped together by cell type identification). Color indicates normalized expression level as shown in legend).

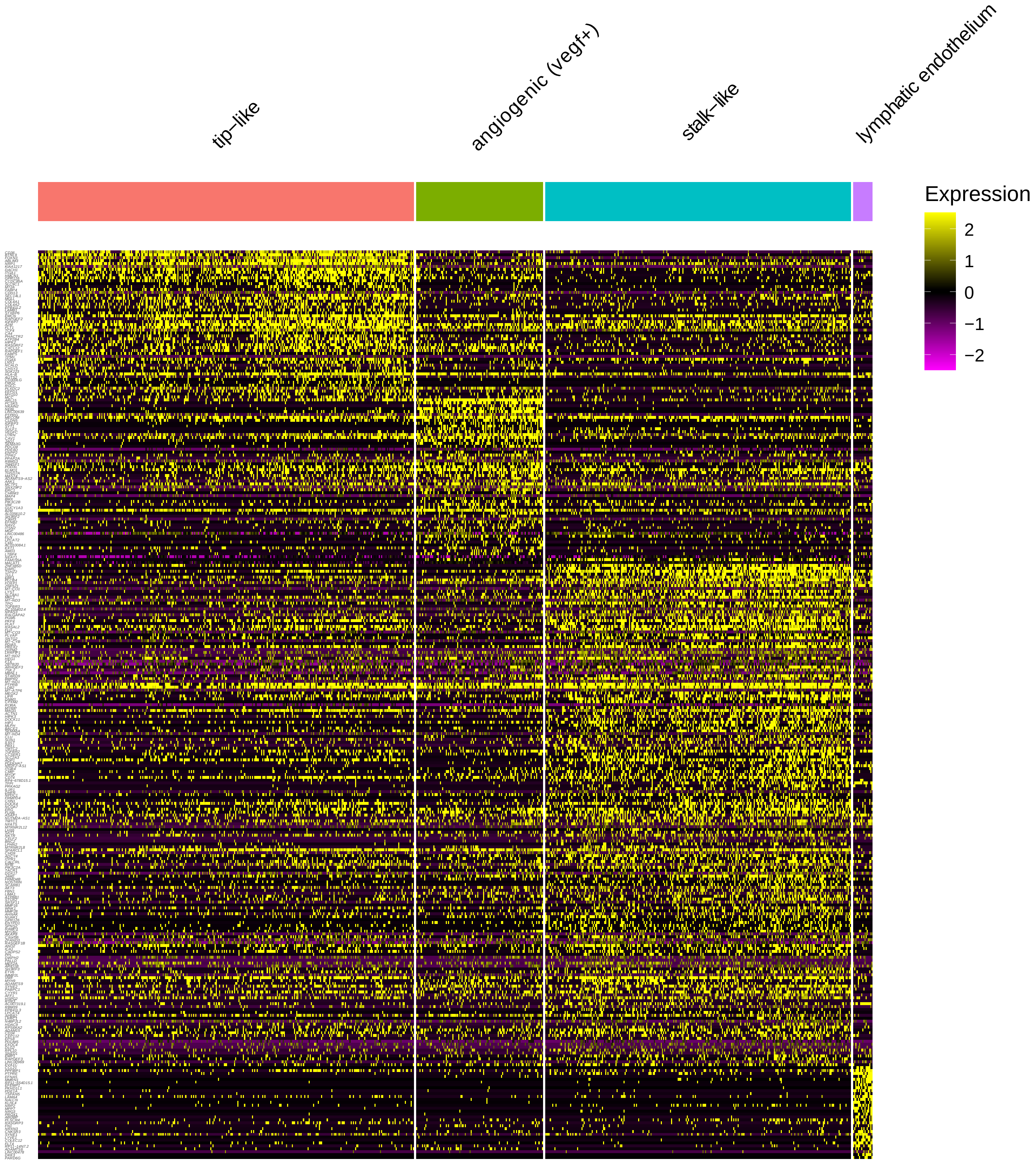

Supplementary Fig. 25: Heat maps human: Endothelial cell subclusters
Heatmap of significant marker genes among human Endothelial cell types. All significant marker genes (Supplementary Data 9) were used to generate these heatmaps, gene names listed on the beginning of each row. Each column represents an individual cell (grouped together by cell type identification). Color indicates normalized expression level as shown in legend).

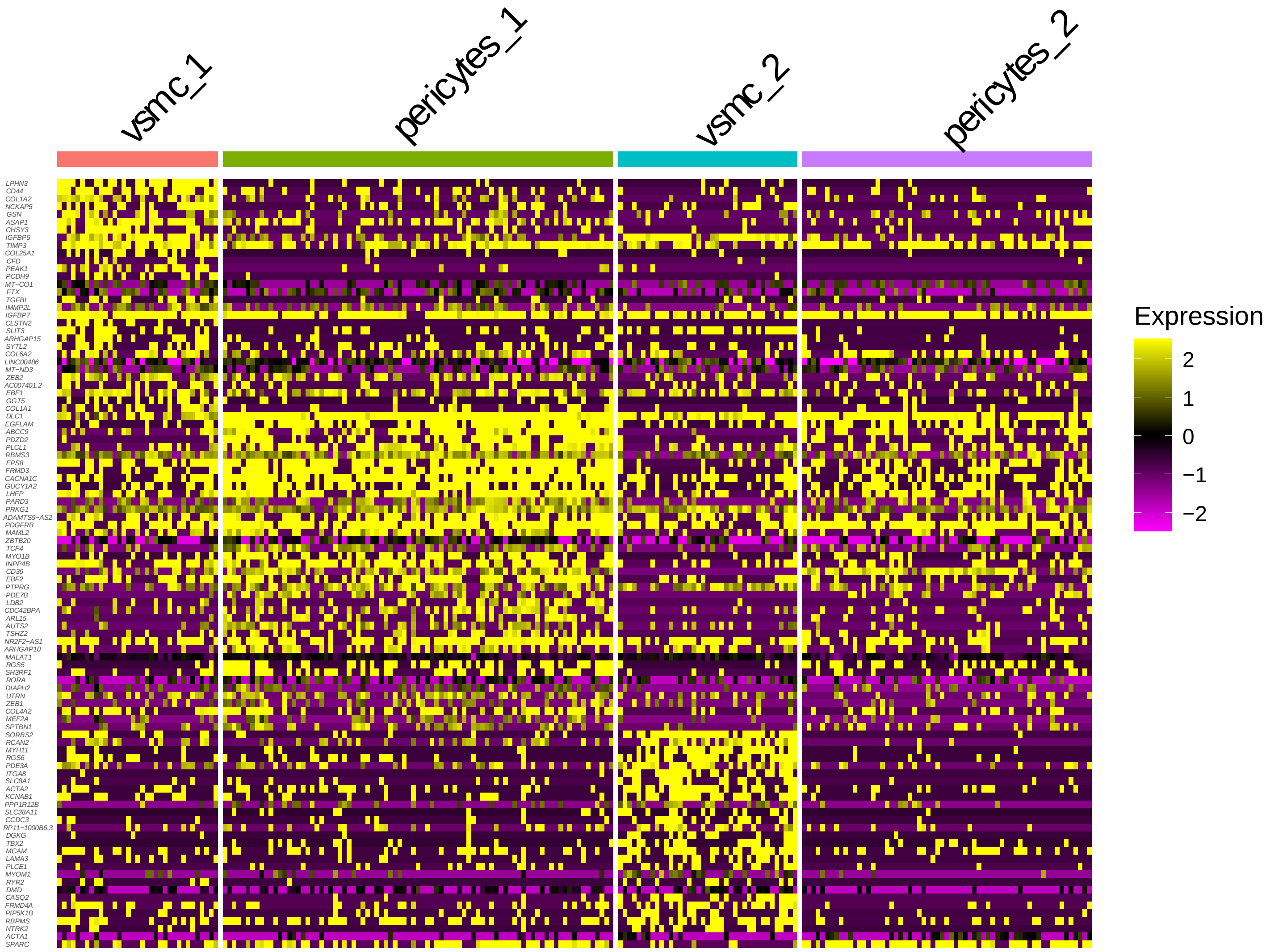

Supplementary Fig. 26: Heat maps human: Smooth muscle subclusters
Heatmap of significant marker genes among human Smooth muscle cell types. All significant marker genes (Supplementary Data 9) were used to generate these heatmaps, gene names listed on the beginning of each row. Each column represents an individual cell (grouped together by cell type identification). Color indicates normalized expression level as shown in legend).

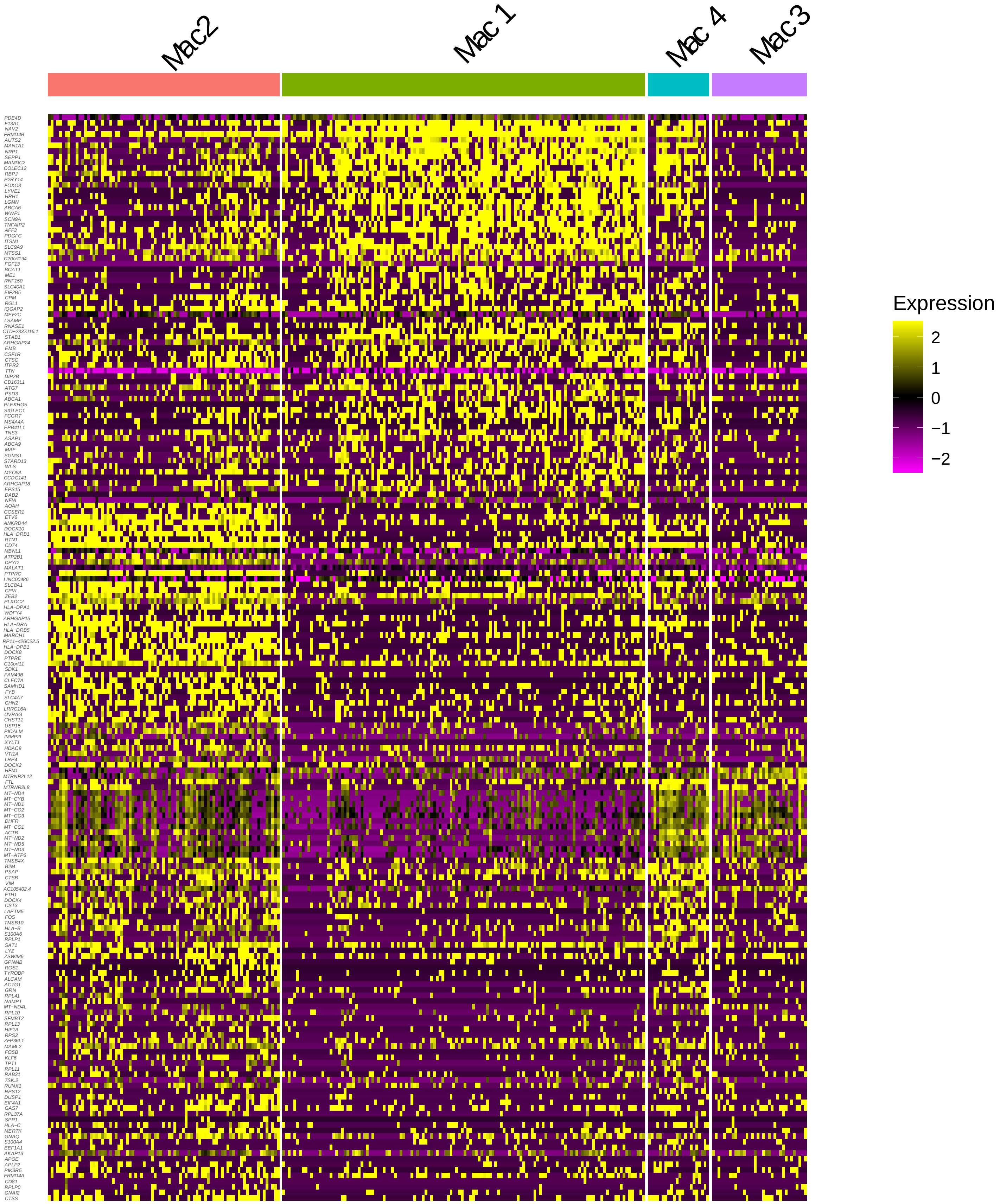

Supplementary Fig. 27: Heat maps human: Macrophage subclusters
Heatmap of significant marker genes among human myofiber cell types. All significant marker genes (Supplementary Data 9) were used to generate these heatmaps, gene names listed on the beginning of each row. Each column represents an individual cell (grouped together by cell type identification). Color indicates normalized expression level as shown in legend).

Supplementary References:

1. H. Yin, F. Price, M. A. Rudnicki, Satellite cells and the muscle stem cell niche. *Physiol Rev* **93**, 23-67 (2013).
2. N. A. Dumont, Y. X. Wang, M. A. Rudnicki, Intrinsic and extrinsic mechanisms regulating satellite cell function. *Development* **142**, 1572-1581 (2015).
3. S. Franco-Iborra, K. Tanji, Histochemical and immunohistochemical staining methods to visualize mitochondrial proteins and activity. *Methods Cell Biol* **155**, 247-270 (2020).
4. K. B. Umansky, Y. Gruenbaum-Cohen, M. Tsoory, E. Feldmesser, D. Goldenberg, O. Brenner, Y. Groner, Runx1 Transcription Factor Is Required for Myoblasts Proliferation during Muscle Regeneration. *PLoS Genet* **11**, e1005457 (2015).
5. M. Agarwal, A. Sharma, P. Kumar, A. Kumar, A. Bharadwaj, M. Saini, G. Kardon, S. J. Mathew, Myosin heavy chain-embryonic regulates skeletal muscle differentiation during mammalian development. *Development* **147**, (2020).
6. K. Ohlendieck, K. Matsumura, V. V. Ionasescu, J. A. Towbin, E. P. Bosch, S. L. Weinstein, S. W. Sernett, K. P. Campbell, Duchenne muscular dystrophy: deficiency of dystrophin-associated proteins in the sarcolemma. *Neurology* **43**, 795-800 (1993).
7. M. A. Lopez, P. S. Pardo, G. A. Cox, A. M. Boriek, Early mechanical dysfunction of the diaphragm in the muscular dystrophy with myositis (Ttnmdm) model. *Am J Physiol Cell Physiol* **295**, C1092-1102 (2008).
8. S. Swist, A. Unger, Y. Li, A. Voge, M. von Frieling-Salewsky, A. Skarlen, N. Cacciani, T. Braun, L. Larsson, W. A. Linke, Maintenance of sarcomeric integrity in adult muscle cells crucially depends on Z-disc anchored titin. *Nat Commun* **11**, 4479 (2020).
9. L. Giordani, G. J. He, E. Negroni, H. Sakai, J. Y. C. Law, M. M. Siu, R. Wan, A. Corneau, S. Tajbakhsh, T. H. Cheung, F. Le Grand, High-Dimensional Single-Cell Cartography Reveals Novel Skeletal Muscle-Resident Cell Populations. *Mol Cell* **74**, 609-621 e606 (2019).
10. L. Muhl, G. Genove, S. Leptidis, J. Liu, L. He, G. Mocci, Y. Sun, S. Gustafsson, B. Buyandelger, I. V. Chivukula, A. Segerstolpe, E. Raschperger, E. M. Hansson, J. L. M. Bjorkegren, X. R. Peng, M. Vanlandewijck, U. Lendahl, C. Betsholtz, Single-cell analysis uncovers fibroblast heterogeneity and criteria for fibroblast and mural cell identification and discrimination. *Nat Commun* **11**, 3953 (2020).
11. N. A. O'Leary, M. W. Wright, J. R. Brister, S. Ciufo, D. Haddad, R. McVeigh, B. Rajput, B. Robbertse, B. Smith-White, D. Ako-Adjei, A. Astashyn, A. Badretdin, Y. Bao, O. Blinkova, V. Brover, V. Chetvernin, J. Choi, E. Cox, O. Ermolaeva, C. M. Farrell, T. Goldfarb, T. Gupta, D. Haft, E. Hatcher, W. Hlavina, V. S. Joardar, V. K. Kodali, W. Li, D. Maglott, P. Masterson, K. M. McGarvey, M. R. Murphy, K. O'Neill, S. Pujar, S. H. Rangwala, D. Rausch, L. D. Riddick, C. Schoch, A. Shkeda, S. S. Storz, H. Sun, F. Thibaud-Nissen, I. Tolstoy, R. E. Tully, A. R. Vatsan, C. Wallin, D. Webb, W. Wu, M. J. Landrum, A. Kimchi, T. Tatusova, M. DiCuccio, P. Kitts, T. D. Murphy, K. D. Pruitt, Reference sequence (RefSeq) database at NCBI: current status, taxonomic expansion, and functional annotation. *Nucleic Acids Res* **44**, D733-745 (2016).
12. K. B. Halpern, R. Shenhav, H. Massalha, B. Toth, A. Egozi, E. E. Massasa, C. Medgalia, E. David, A. Giladi, A. E. Moor, Z. Porat, I. Amit, S. Itzkovitz, Paired-cell sequencing enables spatial gene expression mapping of liver endothelial cells. *Nat Biotechnol* **36**, 962-970 (2018).

13. J. Wilting, M. Papoutsi, B. Christ, K. H. Nicolaides, C. S. von Kaisenberg, J. Borges, G. B. Stark, K. Alitalo, S. I. Tomarev, C. Niemeyer, J. Rossler, The transcription factor Prox1 is a marker for lymphatic endothelial cells in normal and diseased human tissues. *FASEB J* **16**, 1271-1273 (2002).
14. E. Hu, P. Liang, B. M. Spiegelman, AdipoQ is a novel adipose-specific gene dysregulated in obesity. *J Biol Chem* **271**, 10697-10703 (1996).
15. P. Tontonoz, B. M. Spiegelman, Fat and beyond: the diverse biology of PPARgamma. *Annu Rev Biochem* **77**, 289-312 (2008).
16. C. D. Wrann, J. Eguchi, A. Bozec, Z. Xu, T. Mikkelsen, J. Gimble, H. Nave, E. F. Wagner, S. E. Ong, E. D. Rosen, FOSL2 promotes leptin gene expression in human and mouse adipocytes. *J Clin Invest* **122**, 1010-1021 (2012).
17. S. Zhang, G. Liu, C. Xu, L. Liu, Q. Zhang, Q. Xu, H. Jia, X. Li, X. Li, Perilipin 1 Mediates Lipid Metabolism Homeostasis and Inhibits Inflammatory Cytokine Synthesis in Bovine Adipocytes. *Front Immunol* **9**, 467 (2018).
18. M. Ito, M. Nagasawa, T. Hara, T. Ide, K. Murakami, Differential roles of CIDEA and CIDEC in insulin-induced anti-apoptosis and lipid droplet formation in human adipocytes. *J Lipid Res* **51**, 1676-1684 (2010).
19. K. Yaguchi, S. Nishimura-Akiyoshi, S. Kuroki, T. Onodera, S. Itohara, Identification of transcriptional regulatory elements for Ntng1 and Ntng2 genes in mice. *Mol Brain* **7**, 19 (2014).
20. A. Katzman, C. M. Alberini, NLGN1 and NLGN2 in the prefrontal cortex: their role in memory consolidation and strengthening. *Curr Opin Neurobiol* **48**, 122-130 (2018).
21. A. Boerboom, V. Dion, A. Chariot, R. Franzen, Molecular Mechanisms Involved in Schwann Cell Plasticity. *Front Mol Neurosci* **10**, 38 (2017).
22. M. Bremer, F. Frob, T. Kichko, P. Reeh, E. R. Tamm, U. Suter, M. Wegner, Sox10 is required for Schwann-cell homeostasis and myelin maintenance in the adult peripheral nerve. *Glia* **59**, 1022-1032 (2011).
23. G. Militello, M. R. Hosen, Y. Ponomareva, P. Gellert, T. Weirick, D. John, S. M. Hindi, K. Mamchaoui, V. Mouly, C. Doring, L. Zhang, M. Nakamura, A. Kumar, S. I. Fukada, S. Dimmeler, S. Uchida, A novel long non-coding RNA Myolinc regulates myogenesis through TDP-43 and Filip1. *J Mol Cell Biol* **10**, 102-117 (2018).
24. P. Sharma, T. D. Ruel, K. M. Kocha, S. Liao, P. Huang, Single cell dynamics of embryonic muscle progenitor cells in zebrafish. *Development* **146**, (2019).
25. Z. Li, J. A. Gilbert, Y. Zhang, M. Zhang, Q. Qiu, K. Ramanujan, T. Shavlakadze, J. K. Eash, A. Scaramozza, M. M. Goddeeris, D. G. Kirsch, K. P. Campbell, A. S. Brack, D. J. Glass, An HMGA2-IGF2BP2 axis regulates myoblast proliferation and myogenesis. *Dev Cell* **23**, 1176-1188 (2012).
26. S. Schiaffino, A. C. Rossi, V. Smerdu, L. A. Leinwand, C. Reggiani, Developmental myosins: expression patterns and functional significance. *Skelet Muscle* **5**, 22 (2015).
27. M. Olive, Z. Odgerel, A. Martinez, J. J. Poza, F. G. Bragado, R. J. Zabalza, I. Jerico, L. Gonzalez-Mera, A. Shatunov, H. S. Lee, J. Armstrong, E. Maravi, M. R. Arroyo, J. Pascual-Calvet, C. Navarro, C. Paradas, M. Huerta, F. Marquez, E. G. Rivas, A. Pou, I. Ferrer, L. G. Goldfarb, Clinical and myopathological evaluation of early- and late-onset subtypes of myofibrillar myopathy. *Neuromuscul Disord* **21**, 533-542 (2011).
28. B. Charvet, A. Guiraud, M. Malbouyres, D. Zwolanek, E. Guillon, S. Bretaud, C. Monnot, J. Schulze, H. L. Bader, B. Allard, M. Koch, F. Ruggiero, Knockdown of

col22a1 gene in zebrafish induces a muscular dystrophy by disruption of the myotendinous junction. *Development* **140**, 4602-4613 (2013).
29. M. Dos Santos, S. Backer, B. Saintpierre, B. Izac, M. Andrieu, F. Letourneur, F. Relaix, A. Sotiropoulos, P. Maire, Single-nucleus RNA-seq and FISH identify coordinated transcriptional activity in mammalian myofibers. *Nat Commun* **11**, 5102 (2020).
30. A. E. Deconinck, A. C. Potter, J. M. Tinsley, S. J. Wood, R. Vater, C. Young, L. Metzinger, A. Vincent, C. R. Slater, K. E. Davies, Postsynaptic abnormalities at the neuromuscular junctions of utrophin-deficient mice. *J Cell Biol* **136**, 883-894 (1997).
31. X. Lv, F. Gao, T. Dai, D. Zhao, W. Jiang, H. Geng, F. Liu, P. Lin, C. Yan, Distal myopathy due to TCAP variants in four unrelated Chinese patients. *Neurogenetics* **22**, 1-10 (2021).
32. C. D. Markert, M. P. Meaney, K. A. Voelker, R. W. Grange, H. W. Dalley, J. K. Cann, M. Ahmed, B. Bishwokarma, S. J. Walker, S. X. Yu, M. Brown, M. W. Lawlor, A. H. Beggs, M. K. Childers, Functional muscle analysis of the Tcap knockout mouse. *Hum Mol Genet* **19**, 2268-2283 (2010).
33. S. Zhang, P. Londhe, M. Zhang, J. K. Davie, Transcriptional analysis of the titin cap gene. *Mol Genet Genomics* **285**, 261-272 (2011).
34. K. Lukin, S. Fields, J. Hartley, J. Hagman, Early B cell factor: Regulator of B lineage specification and commitment. *Semin Immunol* **20**, 221-227 (2008).
35. G. Pavlasova, M. Mraz, The regulation and function of CD20: an "enigma" of B-cell biology and targeted therapy. *Haematologica* **105**, 1494-1506 (2020).
36. A. Alcover, B. Alarcon, V. Di Bartolo, Cell Biology of T Cell Receptor Expression and Regulation. *Annu Rev Immunol* **36**, 103-125 (2018).
37. N. Dadwal, C. Mix, A. Reinhold, A. Witte, C. Freund, B. Schraven, S. Kliche, The Multiple Roles of the Cytosolic Adapter Proteins ADAP, SKAP1 and SKAP2 for TCR/CD3 -Mediated Signaling Events. *Front Immunol* **12**, 703534 (2021).
38. E. Batlle, J. Massague, Transforming Growth Factor-beta Signaling in Immunity and Cancer. *Immunity* **50**, 924-940 (2019).
39. C. Del Fresno, F. J. Cueto, D. Sancho, Sensing Tissue Damage by Myeloid C-Type Lectin Receptors. *Curr Top Microbiol Immunol* **429**, 117-145 (2020).
40. M. Collin, V. Bigley, Human dendritic cell subsets: an update. *Immunology* **154**, 3-20 (2018).
41. J. Sander, S. V. Schmidt, B. Cirovic, N. McGovern, O. Papantonopoulou, A. L. Hardt, A. C. Aschenbrenner, C. Kreer, T. Quast, A. M. Xu, L. M. Schmidleithner, H. Theis, L. D. Thi Huong, H. R. B. Sumatoh, M. A. R. Lauterbach, J. Schulte-Schrepping, P. Gunther, J. Xue, K. Bassler, T. Ulas, K. Klee, N. Katzmarski, S. Herresthal, W. Krebs, B. Martin, E. Latz, K. Handler, M. Kraut, W. Kolanus, M. Beyer, C. S. Falk, B. Wiegmann, S. Burgdorf, N. A. Melosh, E. W. Newell, F. Ginhoux, A. Schlitzer, J. L. Schultze, Cellular Differentiation of Human Monocytes Is Regulated by Time-Dependent Interleukin-4 Signaling and the Transcriptional Regulator NCOR2. *Immunity* **47**, 1051-1066 e1012 (2017).
42. D. Sichien, C. L. Scott, L. Martens, M. Vanderkerken, S. Van Gassen, M. Plantinga, T. Joeris, S. De Prijck, L. Vanhoutte, M. Vanheerswynghels, G. Van Isterdael, W. Toussaint, F. B. Madeira, K. Vergote, W. W. Agace, B. E. Clausen, H. Hammad, M. Dalod, Y. Saeys, B. N. Lambrecht, M. Guilliams, IRF8 Transcription Factor Controls

Survival and Function of Terminally Differentiated Conventional and Plasmacytoid Dendritic Cells, Respectively. *Immunity* **45**, 626-640 (2016).

43. B. Schroder, The multifaceted roles of the invariant chain CD74--More than just a chaperone. *Biochim Biophys Acta* **1863**, 1269-1281 (2016).

44. M. Swart, L. Troeberg, Effect of Polarization and Chronic Inflammation on Macrophage Expression of Heparan Sulfate Proteoglycans and Biosynthesis Enzymes. *J Histochem Cytochem* **67**, 9-27 (2019).

45. A. Castrillo, D. J. Pennington, F. Otto, P. J. Parker, M. J. Owen, L. Bosca, Protein kinase Cepsilon is required for macrophage activation and defense against bacterial infection. *J Exp Med* **194**, 1231-1242 (2001).

46. M. Orecchioni, Y. Ghosheh, A. B. Pramod, K. Ley, Macrophage Polarization: Different Gene Signatures in M1(LPS+) vs. Classically and M2(LPS-) vs. Alternatively Activated Macrophages. *Front Immunol* **10**, 1084 (2019).

47. F. O. Martinez, S. Gordon, M. Locati, A. Mantovani, Transcriptional profiling of the human monocyte-to-macrophage differentiation and polarization: new molecules and patterns of gene expression. *J Immunol* **177**, 7303-7311 (2006).

48. M. Z. Cader, K. Boroviak, Q. Zhang, G. Assadi, S. L. Kempster, G. W. Sewell, S. Saveljeva, J. W. Ashcroft, S. Clare, S. Mukhopadhyay, K. P. Brown, M. Tschurtschenthaler, T. Raine, B. Doe, E. R. Chilvers, J. L. Griffin, N. C. Kaneider, R. A. Floto, M. D'Amato, A. Bradley, M. J. Wakelam, G. Dougan, A. Kaser, C13orf31 (FAMIN) is a central regulator of immunometabolic function. *Nat Immunol* **17**, 1046-1056 (2016).

49. M. Beyer, M. R. Mallmann, J. Xue, A. Staratschek-Jox, D. Vorholt, W. Krebs, D. Sommer, J. Sander, C. Mertens, A. Nino-Castro, S. V. Schmidt, J. L. Schultze, High-resolution transcriptome of human macrophages. *PLoS One* **7**, e45466 (2012).

50. S. A. Vetrone, E. Montecino-Rodriguez, E. Kudryashova, I. Kramerova, E. P. Hoffman, S. D. Liu, M. C. Miceli, M. J. Spencer, Osteopontin promotes fibrosis in dystrophic mouse muscle by modulating immune cell subsets and intramuscular TGF-beta. *J Clin Invest* **119**, 1583-1594 (2009).

51. C. F. Nielsen, T. Zhang, M. Barisic, P. Kalitsis, D. F. Hudson, Topoisomerase IIalpha is essential for maintenance of mitotic chromosome structure. *Proc Natl Acad Sci U S A* **117**, 12131-12142 (2020).

52. M. Saade, G. Araujo de Souza, C. Scavone, P. F. Kinoshita, The Role of GPNMB in Inflammation. *Front Immunol* **12**, 674739 (2021).

53. H. Lewinsky, A. F. Barak, V. Huber, M. P. Kramer, L. Radomir, L. Sever, I. Orr, V. Mirkin, N. Dezorella, M. Shapiro, Y. Cohen, L. Shvidel, M. Seiffert, Y. Herishanu, S. Becker-Herman, I. Shachar, CD84 regulates PD-1/PD-L1 expression and function in chronic lymphocytic leukemia. *J Clin Invest* **128**, 5465-5478 (2018).

54. C. Tesson, M. Nawara, M. A. Salih, R. Rossignol, M. S. Zaki, M. Al Balwi, R. Schule, C. Mignot, E. Obre, A. Bouhouche, F. M. Santorelli, C. M. Durand, A. C. Oteyza, K. H. El-Hachimi, A. Al Drees, N. Bouslam, F. Lamari, S. A. Elmalik, M. M. Kabiraj, M. Z. Seidahmed, T. Esteves, M. Gaussen, M. L. Monin, G. Gyapay, D. Lechner, M. Gonzalez, C. Depienne, F. Mochel, J. Lavie, L. Schols, D. Lacombe, M. Yahyaoui, I. Al Abdulkareem, S. Zuchner, A. Yamashita, A. Benomar, C. Goizet, A. Durr, J. G. Gleeson, F. Darios, A. Brice, G. Stevanin, Alteration of fatty-acid-metabolizing enzymes affects

mitochondrial form and function in hereditary spastic paraplegia. *Am J Hum Genet* **91**, 1051-1064 (2012).

55. J. Miska, A. Rashidi, C. Lee-Chang, P. Gao, A. Lopez-Rosas, P. Zhang, R. Burga, B. Castro, T. Xiao, Y. Han, D. Hou, S. Sampat, A. Cordero, J. S. Stoolman, C. M. Horbinski, M. Burns, Y. K. Reshetnyak, N. S. Chandel, M. S. Lesniak, Polyamines drive myeloid cell survival by buffering intracellular pH to promote immunosuppression in glioblastoma. *Sci Adv* **7**, (2021).
56. D. Vats, L. Mukundan, J. I. Odegaard, L. Zhang, K. L. Smith, C. R. Morel, R. A. Wagner, D. R. Greaves, P. J. Murray, A. Chawla, Oxidative metabolism and PGC-1beta attenuate macrophage-mediated inflammation. *Cell Metab* **4**, 13-24 (2006).
57. F. Sallusto, C. R. Mackay, A. Lanzavecchia, Selective expression of the eotaxin receptor CCR3 by human T helper 2 cells. *Science* **277**, 2005-2007 (1997).
58. M. Z. Adzemovic, J. Ockinger, M. Zeitelhofer, S. Hochmeister, A. D. Beyeen, A. Paulson, A. Gillett, M. Thessen Hedreul, R. Covacu, H. Lassmann, T. Olsson, M. Jagodic, Expression of Ccl11 associates with immune response modulation and protection against neuroinflammation in rats. *PLoS One* **7**, e39794 (2012).
59. K. B. Hansen, C. Staehr, P. D. Rohde, C. Homilius, S. Kim, M. Nyegaard, V. V. Matchkov, E. Boedtkjer, PTPRG is an ischemia risk locus essential for HCO3(-)-dependent regulation of endothelial function and tissue perfusion. *Elife* **9**, (2020).
60. A. Costa, Y. Kieffer, A. Scholer-Dahirel, F. Pelon, B. Bourachot, M. Cardon, P. Sirven, I. Magagna, L. Fuhrmann, C. Bernard, C. Bonneau, M. Kondratova, I. Kuperstein, A. Zinovyev, A. M. Givel, M. C. Parrini, V. Soumelis, A. Vincent-Salomon, F. Mechta-Grigoriou, Fibroblast Heterogeneity and Immunosuppressive Environment in Human Breast Cancer. *Cancer Cell* **33**, 463-479 e410 (2018).
61. J. Liu, T. Miwa, B. Hilliard, Y. Chen, J. D. Lambris, A. D. Wells, W. C. Song, The complement inhibitory protein DAF (CD55) suppresses T cell immunity in vivo. *J Exp Med* **201**, 567-577 (2005).
62. A. Lorts, J. A. Schwanekamp, T. A. Baudino, E. M. McNally, J. D. Molkentin, Deletion of periostin reduces muscular dystrophy and fibrosis in mice by modulating the transforming growth factor-beta pathway. *Proc Natl Acad Sci U S A* **109**, 10978-10983 (2012).
63. A. E. Papathanassiu, J. H. Ko, M. Imprialou, M. Bagnati, P. K. Srivastava, H. A. Vu, D. Cucchi, S. P. McAdoo, E. A. Ananieva, C. Mauro, J. Behmoaras, BCAT1 controls metabolic reprogramming in activated human macrophages and is associated with inflammatory diseases. *Nat Commun* **8**, 16040 (2017).
64. Y. J. Kwon, S. W. Lee, Y. B. Park, S. K. Lee, M. C. Park, Secreted frizzled-related protein 5 suppresses inflammatory response in rheumatoid arthritis fibroblast-like synoviocytes through down-regulation of c-Jun N-terminal kinase. *Rheumatology (Oxford)* **53**, 1704-1711 (2014).
65. C. C. Deng, Y. F. Hu, D. H. Zhu, Q. Cheng, J. J. Gu, Q. L. Feng, L. X. Zhang, Y. P. Xu, D. Wang, Z. Rong, B. Yang, Single-cell RNA-seq reveals fibroblast heterogeneity and increased mesenchymal fibroblasts in human fibrotic skin diseases. *Nat Commun* **12**, 3709 (2021).
66. M. Koch, J. Schulze, U. Hansen, T. Ashwodt, D. R. Keene, W. J. Brunken, R. E. Burgeson, P. Bruckner, L. Bruckner-Tuderman, A novel marker of tissue junctions, collagen XXII. *J Biol Chem* **279**, 22514-22521 (2004).

67. M. Decker, L. Martinez-Morentin, G. Wang, Y. Lee, Q. Liu, J. Leslie, L. Ding, Leptin-receptor-expressing bone marrow stromal cells are myofibroblasts in primary myelofibrosis. *Nat Cell Biol* **19**, 677-688 (2017).
68. L. Xie, M. Takahara, T. Nakahara, J. Oba, H. Uchi, S. Takeuchi, Y. Moroi, M. Furue, CD10-bearing fibroblasts may inhibit skin inflammation by down-modulating substance P. *Arch Dermatol Res* **303**, 49-55 (2011).
69. X. Pan, B. Liu, S. Chen, H. Ding, X. Yao, Y. Cheng, D. Xu, Y. Yin, X. Dai, J. Sun, G. Xu, J. Pan, L. Xiao, L. Xie, Nr4a1 as a myogenic factor is upregulated in satellite cells/myoblast under proliferation and differentiation state. *Biochem Biophys Res Commun* **513**, 573-581 (2019).
70. C. Bondjers, M. Kalen, M. Hellstrom, S. J. Scheidl, A. Abramsson, O. Renner, P. Lindahl, H. Cho, J. Kehrl, C. Betsholtz, Transcription profiling of platelet-derived growth factor-B-deficient mouse embryos identifies RGS5 as a novel marker for pericytes and vascular smooth muscle cells. *Am J Pathol* **162**, 721-729 (2003).
71. Y. Zhang, Y. Wang, L. Zhang, L. Xia, M. Zheng, Z. Zeng, Y. Liu, T. Yarovinsky, A. C. Ostriker, X. Fan, K. Weng, M. Su, P. Huang, K. A. Martin, J. Hwa, W. H. Tang, Reduced Platelet miR-223 Induction in Kawasaki Disease Leads to Severe Coronary Artery Pathology Through a miR-223/PDGFRbeta Vascular Smooth Muscle Cell Axis. *Circ Res* **127**, 855-873 (2020).
72. Z. Wang, D. Z. Wang, G. C. Pipes, E. N. Olson, Myocardin is a master regulator of smooth muscle gene expression. *Proc Natl Acad Sci U S A* **100**, 7129-7134 (2003).
73. A. S. Verkman, Aquaporin water channels and endothelial cell function. *J Anat* **200**, 617-627 (2002).
74. W. Chen, P. Xia, H. Wang, J. Tu, X. Liang, X. Zhang, L. Li, The endothelial tip-stalk cell selection and shuffling during angiogenesis. *J Cell Commun Signal* **13**, 291-301 (2019).
75. E. A. Koop, S. M. Lopes, E. Feiken, H. A. Bluyssen, M. van der Valk, E. E. Voest, C. L. Mummery, W. H. Moolenaar, M. F. Gebbink, Receptor protein tyrosine phosphatase mu expression as a marker for endothelial cell heterogeneity; analysis of RPTPmu gene expression using LacZ knock-in mice. *Int J Dev Biol* **47**, 345-354 (2003).
76. A. H. Chang, B. C. Raftrey, G. D'Amato, V. N. Surya, A. Poduri, H. I. Chen, A. B. Goldstone, J. Woo, G. G. Fuller, A. R. Dunn, K. Red-Horse, DACH1 stimulates shear stress-guided endothelial cell migration and coronary artery growth through the CXCL12-CXCR4 signaling axis. *Genes Dev* **31**, 1308-1324 (2017).
77. Z. J. Liu, T. Shirakawa, Y. Li, A. Soma, M. Oka, G. P. Dotto, R. M. Fairman, O. C. Velazquez, M. Herlyn, Regulation of Notch1 and Dll4 by vascular endothelial growth factor in arterial endothelial cells: implications for modulating arteriogenesis and angiogenesis. *Mol Cell Biol* **23**, 14-25 (2003).
78. J. Lindqvist, Y. Levy, A. Pati-Alam, E. C. Hardeman, P. Gregorevic, J. Ochala, Modulating myosin restores muscle function in a mouse model of nemaline myopathy. *Ann Neurol* **79**, 717-725 (2016).
79. M. W. Lawlor, E. T. Dechene, E. Roumm, A. S. Geggel, B. Moghadaszadeh, A. H. Beggs, Mutations of tropomyosin 3 (TPM3) are common and associated with type 1 myofiber hypotrophy in congenital fiber type disproportion. *Hum Mutat* **31**, 176-183 (2010).

80. H. Z. Feng, J. P. Jin, Carbonic Anhydrase III Is Expressed in Mouse Skeletal Muscles Independent of Fiber Type-Specific Myofilament Protein Isoforms and Plays a Role in Fatigue Resistance. *Front Physiol* **7**, 597 (2016).
81. L. Li, X. Cheng, L. Chen, J. Li, W. Luo, C. Li, Long Noncoding Ribonucleic Acid MSTRG.59589 Promotes Porcine Skeletal Muscle Satellite Cells Differentiation by Enhancing the Function of PALLD. *Front Genet* **10**, 1220 (2019).
82. Y. K. Kim, R. S. Yadava, M. Mandal, K. Mahadevan, Q. Yu, M. Leitges, M. S. Mahadevan, Disease Phenotypes in a Mouse Model of RNA Toxicity Are Independent of Protein Kinase Calpha and Protein Kinase Cbeta. *PLoS One* **11**, e0163325 (2016).
83. M. Pescatori, A. Broccolini, C. Minetti, E. Bertini, C. Bruno, A. D'Amico, C. Bernardini, M. Mirabella, G. Silvestri, V. Giglio, A. Modoni, M. Pedemonte, G. Tasca, G. Galluzzi, E. Mercuri, P. A. Tonali, E. Ricci, Gene expression profiling in the early phases of DMD: a constant molecular signature characterizes DMD muscle from early postnatal life throughout disease progression. *FASEB J* **21**, 1210-1226 (2007).
84. Y. Zhang, H. Yan, P. Zhou, Z. Zhang, J. Liu, H. Zhang, MicroRNA-152 Promotes Slow-Twitch Myofiber Formation via Targeting Uncoupling Protein-3 Gene. *Animals (Basel)* **9**, (2019).
85. E. J. Anderson, H. Yamazaki, P. D. Neufer, Induction of endogenous uncoupling protein 3 suppresses mitochondrial oxidant emission during fatty acid-supported respiration. *J Biol Chem* **282**, 31257-31266 (2007).
86. C. M. Adams, M. Suneja, S. Dudley-Javoroski, R. K. Shields, Altered mRNA expression after long-term soleus electrical stimulation training in humans with paralysis. *Muscle Nerve* **43**, 65-75 (2011).
87. J. P. Orengo, A. J. Ward, T. A. Cooper, Alternative splicing dysregulation secondary to skeletal muscle regeneration. *Ann Neurol* **69**, 681-690 (2011).
88. J. Etienne, P. Joanne, C. Catelain, S. Riveron, A. C. Bayer, J. Lafable, I. Punzon, S. Blot, O. Agbulut, J. T. Vilquin, Aldehyde dehydrogenases contribute to skeletal muscle homeostasis in healthy, aging, and Duchenne muscular dystrophy patients. *J Cachexia Sarcopenia Muscle* **11**, 1047-1069 (2020).
89. V. De Arcangelis, G. Strimpakos, F. Gabanella, N. Corbi, S. Luvisetto, A. Magrelli, A. Onori, C. Passananti, C. Pisani, S. Rome, C. Severini, F. Naro, E. Mattei, M. G. Di Certo, L. Monaco, Pathways Implicated in Tadalafil Amelioration of Duchenne Muscular Dystrophy. *J Cell Physiol* **231**, 224-232 (2016).
90. M. A. Ackermann, C. W. Ward, C. Gurnett, A. Kontrogianni-Konstantopoulos, Myosin Binding Protein-C Slow Phosphorylation is Altered in Duchenne Dystrophy and Arthrogryposis Myopathy in Fast-Twitch Skeletal Muscles. *Sci Rep* **5**, 13235 (2015).
91. Y. Nio, M. Tanaka, Y. Hirozane, Y. Muraki, M. Okawara, M. Hazama, T. Matsuo, Phosphodiesterase 4 inhibitor and phosphodiesterase 5 inhibitor combination therapy has antifibrotic and anti-inflammatory effects in mdx mice with Duchenne muscular dystrophy. *FASEB J* **31**, 5307-5320 (2017).
92. W. Qin, J. Pan, Y. Wu, W. A. Bauman, C. Cardozo, Anabolic steroids activate calcineurin-NFAT signaling and thereby increase myotube size and reduce denervation atrophy. *Mol Cell Endocrinol* **399**, 336-345 (2015).
93. E. Murani, M. Muraniova, S. Ponsuksili, K. Schellander, K. Wimmers, Identification of genes differentially expressed during prenatal development of skeletal muscle in two pig breeds differing in muscularity. *BMC Dev Biol* **7**, 109 (2007).

94. N. Hagiwara, M. Yeh, A. Liu, Sox6 is required for normal fiber type differentiation of fetal skeletal muscle in mice. *Dev Dyn* **236**, 2062-2076 (2007).
95. J. Stockli, C. C. Meoli, N. J. Hoffman, D. J. Fazakerley, H. Pant, M. E. Cleasby, X. Ma, M. Kleinert, A. E. Brandon, J. A. Lopez, G. J. Cooney, D. E. James, The RabGAP TBC1D1 plays a central role in exercise-regulated glucose metabolism in skeletal muscle. *Diabetes* **64**, 1914-1922 (2015).
96. K. J. Sonnemann, D. P. Fitzsimons, J. R. Patel, Y. Liu, M. F. Schneider, R. L. Moss, J. M. Ervasti, Cytoplasmic gamma-actin is not required for skeletal muscle development but its absence leads to a progressive myopathy. *Dev Cell* **11**, 387-397 (2006).
97. K. W. Prins, D. A. Lowe, J. M. Ervasti, Skeletal muscle-specific ablation of gamma(cyto)-actin does not exacerbate the mdx phenotype. *PLoS One* **3**, e2419 (2008).
98. Y. C. Long, S. Glund, P. M. Garcia-Roves, J. R. Zierath, Calcineurin regulates skeletal muscle metabolism via coordinated changes in gene expression. *J Biol Chem* **282**, 1607-1614 (2007).
99. I. Holt, V. Jacquemin, M. Fardaei, C. A. Sewry, G. S. Butler-Browne, D. Furling, J. D. Brook, G. E. Morris, Muscleblind-like proteins: similarities and differences in normal and myotonic dystrophy muscle. *Am J Pathol* **174**, 216-227 (2009).
100. G. Chen, A. Masuda, H. Konishi, B. Ohkawara, M. Ito, M. Kinoshita, H. Kiyama, T. Matsuura, K. Ohno, Phenylbutazone induces expression of MBNL1 and suppresses formation of MBNL1-CUG RNA foci in a mouse model of myotonic dystrophy. *Sci Rep* **6**, 25317 (2016).
101. J. Kasch, I. Kanzleiter, S. Saussenthaler, A. Schurmann, J. Keijer, E. van Schothorst, S. Klaus, S. Schumann, Insulin sensitivity linked skeletal muscle Nr4a1 DNA methylation is programmed by the maternal diet and modulated by voluntary exercise in mice. *J Nutr Biochem* **57**, 86-92 (2018).
102. Y. Yoshimoto, M. Ikemoto-Uezumi, K. Hitachi, S. I. Fukada, A. Uezumi, Methods for Accurate Assessment of Myofiber Maturity During Skeletal Muscle Regeneration. *Front Cell Dev Biol* **8**, 267 (2020).
103. L. L. Smith, V. A. Gupta, A. H. Beggs, Bridging integrator 1 (Bin1) deficiency in zebrafish results in centronuclear myopathy. *Hum Mol Genet* **23**, 3566-3578 (2014).
104. I. Prokic, B. S. Cowling, C. Kutchukian, C. Kretz, H. Tasfaout, V. Gache, J. Hergueux, O. Wendling, A. Ferry, A. Toussaint, C. Gavriilidis, V. Nattarayan, C. Koch, J. Laine, R. Combe, L. Tiret, V. Jacquemond, F. Pilot-Storck, J. Laporte, Differential physiological roles for BIN1 isoforms in skeletal muscle development, function and regeneration. *Dis Model Mech* **13**, (2020).
105. L. J. Kepser, F. Damar, T. De Cicco, C. Chaponnier, T. J. Proszynski, A. Pagenstecher, M. B. Rust, CAP2 deficiency delays myofibril actin cytoskeleton differentiation and disturbs skeletal muscle architecture and function. *Proc Natl Acad Sci U S A* **116**, 8397-8402 (2019).
106. M. Zhang, J. Liu, A. Cheng, S. M. Deyoung, A. R. Saltiel, Identification of CAP as a costameric protein that interacts with filamin C. *Mol Biol Cell* **18**, 4731-4740 (2007).
107. C. Song, J. Wang, Y. Ma, Z. Yang, D. Dong, H. Li, J. Yang, Y. Huang, M. Plath, Y. Ma, H. Chen, Linc-smad7 promotes myoblast differentiation and muscle regeneration via sponging miR-125b. *Epigenetics* **13**, 591-604 (2018).
108. P. Kaliman, E. Llagostera, Myotonic dystrophy protein kinase (DMPK) and its role in the pathogenesis of myotonic dystrophy 1. *Cell Signal* **20**, 1935-1941 (2008).

109. L. Jensen, S. J. Petersson, N. O. Illum, H. C. Laugaard-Jacobsen, T. Thelle, L. H. Jorgensen, H. D. Schroder, Muscular response to the first three months of deflazacort treatment in boys with Duchenne muscular dystrophy. *J Musculoskelet Neuronal Interact* **17**, 8-18 (2017).
110. I. Dalkilic, J. Schienda, T. G. Thompson, L. M. Kunkel, Loss of FilaminC (FLNc) results in severe defects in myogenesis and myotube structure. *Mol Cell Biol* **26**, 6522-6534 (2006).
111. Z. Chen, N. Bu, X. Qiao, Z. Zuo, Y. Shu, Z. Liu, Z. Qian, J. Chen, Y. Hou, Forkhead Box M1 Transcriptionally Regulates the Expression of Long Noncoding RNAs Snhg8 and Gm26917 to Promote Proliferation and Survival of Muscle Satellite Cells. *Stem Cells* **36**, 1097-1108 (2018).
112. K. Mukund, S. Subramaniam, Dysregulated mechanisms underlying Duchenne muscular dystrophy from co-expression network preservation analysis. *BMC Res Notes* **8**, 182 (2015).
113. X. Xu, B. Mishra, N. Qin, X. Sun, S. Zhang, J. Yang, R. Xu, Differential Transcriptome Analysis of Early Postnatal Developing Longissimus Dorsi Muscle from Two Pig Breeds Characterized in Divergent Myofiber Traits and Fatness. *Anim Biotechnol* **30**, 63-74 (2019).
114. L. Formicola, G. Marazzi, D. A. Sassoon, The extraocular muscle stem cell niche is resistant to ageing and disease. *Front Aging Neurosci* **6**, 328 (2014).
115. S. M. Ebert, A. M. Monteys, D. K. Fox, K. S. Bongers, B. E. Shields, S. E. Malmberg, B. L. Davidson, M. Suneja, C. M. Adams, The transcription factor ATF4 promotes skeletal myofiber atrophy during fasting. *Mol Endocrinol* **24**, 790-799 (2010).
116. M. Zhang, Y. Han, J. Liu, L. Liu, L. Zheng, Y. Chen, R. Xia, D. Yao, X. Cai, X. Xu, Rbm24 modulates adult skeletal muscle regeneration via regulation of alternative splicing. *Theranostics* **10**, 11159-11177 (2020).
117. C. F. Spurney, S. Knoblach, E. E. Pistilli, K. Nagaraju, G. R. Martin, E. P. Hoffman, Dystrophin-deficient cardiomyopathy in mouse: expression of Nox4 and Lox are associated with fibrosis and altered functional parameters in the heart. *Neuromuscul Disord* **18**, 371-381 (2008).
118. R. Klinck, A. Fourrier, P. Thibault, J. Toutant, M. Durand, E. Lapointe, M. L. Caillet-Boudin, N. Sergeant, G. Gourdon, G. Meola, D. Furling, J. Puymirat, B. Chabot, RBFOX1 cooperates with MBNL1 to control splicing in muscle, including events altered in myotonic dystrophy type 1. *PLoS One* **9**, e107324 (2014).
119. P. Meinke, A. R. W. Kerr, R. Czapiewski, J. I. de Las Heras, C. R. Dixon, E. Harris, H. Kolbel, F. Muntoni, U. Schara, V. Straub, B. Schoser, M. Wehnert, E. C. Schirmer, A multistage sequencing strategy pinpoints novel candidate alleles for Emery-Dreifuss muscular dystrophy and supports gene misregulation as its pathomechanism. *EBioMedicine* **51**, 102587 (2020).
120. H. Xiong, Novel candidate alleles associated with gene regulation for Emery-Dreifuss muscular dystrophy. *EBioMedicine* **52**, 102620 (2020).
121. S. Hintze, L. Knaier, S. Limmer, B. Schoser, P. Meinke, Nuclear Envelope Transmembrane Proteins in Myotonic Dystrophy Type 1. *Front Physiol* **9**, 1532 (2018).
122. M. I. Robson, J. I. de Las Heras, R. Czapiewski, P. Le Thanh, D. G. Booth, D. A. Kelly, S. Webb, A. R. W. Kerr, E. C. Schirmer, Tissue-Specific Gene Repositioning by Muscle

Nuclear Membrane Proteins Enhances Repression of Critical Developmental Genes during Myogenesis. *Mol Cell* **62**, 834-847 (2016).
123. R. Watts, V. L. Johnsen, J. Shearer, D. S. Hittel, Myostatin-induced inhibition of the long noncoding RNA Malat1 is associated with decreased myogenesis. *Am J Physiol Cell Physiol* **304**, C995-1001 (2013).
124. G. Arun, S. Diermeier, M. Akerman, K. C. Chang, J. E. Wilkinson, S. Hearn, Y. Kim, A. R. MacLeod, A. R. Krainer, L. Norton, E. Brogi, M. Egeblad, D. L. Spector, Differentiation of mammary tumors and reduction in metastasis upon Malat1 lncRNA loss. *Genes Dev* **30**, 34-51 (2016).
125. M. R. Lambert, J. M. Spinazzola, J. J. Widrick, A. Pakula, J. R. Conner, J. E. Chin, J. M. Owens, L. M. Kunkel, PDE10A Inhibition Reduces the Manifestation of Pathology in DMD Zebrafish and Represses the Genetic Modifier PITPNA. *Mol Ther* **29**, 1086-1101 (2021).
126. R. S. Ghadiali, S. E. Guimond, J. E. Turnbull, A. Pisconti, Dynamic changes in heparan sulfate during muscle differentiation and ageing regulate myoblast cell fate and FGF2 signalling. *Matrix Biol* **59**, 54-68 (2017).
127. X. F. Li, Z. Q. Wang, L. Y. Li, G. Q. Zhao, S. N. Yu, Downregulation of the long noncoding RNA MBNL1-AS1 protects sevoflurane-pretreated mice against ischemia-reperfusion injury by targeting KCNMA1. *Exp Mol Med* **50**, 1-16 (2018).
128. H. Karvonen, K. Summala, W. Niininen, H. R. Barker, D. Ungureanu, Interaction between ROR1 and MuSK activation complex in myogenic cells. *FEBS Lett* **592**, 434-445 (2018).
129. N. Mokbel, N. J. Hoffman, C. M. Girgis, L. Small, N. Turner, R. J. Daly, G. J. Cooney, L. J. Holt, Grb10 deletion enhances muscle cell proliferation, differentiation and GLUT4 plasma membrane translocation. *J Cell Physiol* **229**, 1753-1764 (2014).
130. H. Crossland, P. J. Atherton, A. Stromberg, T. Gustafsson, J. A. Timmons, A reverse genetics cell-based evaluation of genes linked to healthy human tissue age. *FASEB J* **31**, 96-108 (2017).
131. C. T. J. van Velthoven, A. de Morree, I. M. Egner, J. O. Brett, T. A. Rando, Transcriptional Profiling of Quiescent Muscle Stem Cells In Vivo. *Cell Rep* **21**, 1994-2004 (2017).
132. O. Seda, L. Sedova, J. Vcelak, M. Vankova, F. Liska, B. Bendlova, ZBTB16 and metabolic syndrome: a network perspective. *Physiol Res* **66**, S357-S365 (2017).
133. B. Ortiz, J. R. White, W. H. Wu, T. A. Chan, Deletion of Ptprd and Cdkn2a cooperate to accelerate tumorigenesis. *Oncotarget* **5**, 6976-6982 (2014).
134. S. F. Jeng, C. S. Rau, P. C. Liliang, C. J. Wu, T. H. Lu, Y. C. Chen, C. J. Lin, C. H. Hsieh, Profiling muscle-specific microRNA expression after peripheral denervation and reinnervation in a rat model. *J Neurotrauma* **26**, 2345-2353 (2009).
135. I. Castiglioni, R. Caccia, J. M. Garcia-Manteiga, G. Ferri, G. Caretti, I. Molineris, K. Nishioka, D. Gabellini, The Trithorax protein Ash1L promotes myoblast fusion by activating Cdon expression. *Nat Commun* **9**, 5026 (2018).
136. Z. Budai, L. Balogh, Z. Sarang, Altered Gene Expression of Muscle Satellite Cells Contributes to Agerelated Sarcopenia in Mice. *Curr Aging Sci* **11**, 165-172 (2018).
137. X. Li, Z. Wang, H. Tong, Y. Yan, S. Li, Effects of COL8A1 on the proliferation of muscle-derived satellite cells. *Cell Biol Int* **42**, 1132-1140 (2018).

138. M. J. Kim, D. Febbraro, S. Farkona, T. Gillmore, J. E. Son, R. Regeenes, H. H. Chang, E. Pollock-Tahiri, J. Yang, Y. J. Park, T. Sivasubramaniyam, S. J. Oh, P. Saraon, I. Stagljar, J. V. Rocheleau, C. C. Hui, I. Caniggia, Z. Hao, T. W. Mak, A. Konvalinka, M. Woo, Distinct roles of UVRAG and EGFR signaling in skeletal muscle homeostasis. *Mol Metab* **47**, 101185 (2021).
139. M. Rodriguez-Moyano, I. Diaz, N. Dionisio, X. Zhang, J. Avila-Medina, E. Calderon-Sanchez, M. Trebak, J. A. Rosado, A. Ordonez, T. Smani, Urotensin-II promotes vascular smooth muscle cell proliferation through store-operated calcium entry and EGFR transactivation. *Cardiovasc Res* **100**, 297-306 (2013).
140. Y. X. Wang, P. Feige, C. E. Brun, B. Hekmatnejad, N. A. Dumont, J. M. Renaud, S. Faulkes, D. E. Guindon, M. A. Rudnicki, EGFR-Aurka Signaling Rescues Polarity and Regeneration Defects in Dystrophin-Deficient Muscle Stem Cells by Increasing Asymmetric Divisions. *Cell Stem Cell* **24**, 419-432 e416 (2019).
141. J. E. Morgan, P. S. Zammit, Direct effects of the pathogenic mutation on satellite cell function in muscular dystrophy. *Exp Cell Res* **316**, 3100-3108 (2010).
142. P. C. G. Onofre-Oliveira, M. Vainzof, Isolation and Characterization of Muscle-Derived Stem Cells from Dystrophic Mouse Models. *Methods Mol Biol* **2063**, 171-180 (2020).
143. J. Gagan, B. K. Dey, R. Layer, Z. Yan, A. Dutta, Notch3 and Mef2c proteins are mutually antagonistic via Mkp1 protein and miR-1/206 microRNAs in differentiating myoblasts. *J Biol Chem* **287**, 40360-40370 (2012).
144. T. Kitamoto, K. Hanaoka, Notch3 null mutation in mice causes muscle hyperplasia by repetitive muscle regeneration. *Stem Cells* **28**, 2205-2216 (2010).
145. K. Kamizaki, M. Endo, Y. Minami, Y. Kobayashi, Role of noncanonical Wnt ligands and Ror-family receptor tyrosine kinases in the development, regeneration, and diseases of the musculoskeletal system. *Dev Dyn* **250**, 27-38 (2021).
146. A. Sarkozy, A. R. Foley, A. A. Zambon, C. G. Bonnemann, F. Muntoni, LAMA2-Related Dystrophies: Clinical Phenotypes, Disease Biomarkers, and Clinical Trial Readiness. *Front Mol Neurosci* **13**, 123 (2020).
147. N. Tubau-Juni, J. Bassaganya-Riera, A. Leber, V. Zoccoli-Rodriguez, B. Kronsteiner, M. Viladomiu, V. Abedi, C. W. Philipson, R. Hontecillas, Identification of new regulatory genes through expression pattern analysis of a global RNA-seq dataset from a Helicobacter pylori co-culture system. *Sci Rep* **10**, 11506 (2020).
148. M. W. Kuo, C. H. Wang, H. C. Wu, S. J. Chang, Y. J. Chuang, Soluble THSD7A is an N-glycoprotein that promotes endothelial cell migration and tube formation in angiogenesis. *PLoS One* **6**, e29000 (2011).
149. J. Trombetta-Esilva, A. D. Bradshaw, The Function of SPARC as a Mediator of Fibrosis. *Open Rheumatol J* **6**, 146-155 (2012).
150. M. A. Karsdal, S. H. Nielsen, D. J. Leeming, L. L. Langholm, M. J. Nielsen, T. Manon-Jensen, A. Siebuhr, N. S. Gudmann, S. Ronnow, J. M. Sand, S. J. Daniels, J. H. Mortensen, D. Schuppan, The good and the bad collagens of fibrosis - Their role in signaling and organ function. *Adv Drug Deliv Rev* **121**, 43-56 (2017).
151. X. Hua, Y. Y. Wang, P. Jia, Q. Xiong, Y. Hu, Y. Chang, S. Lai, Y. Xu, Z. Zhao, J. Song, Multi-level transcriptome sequencing identifies COL1A1 as a candidate marker in human heart failure progression. *BMC Med* **18**, 2 (2020).

152. M. Fragiadaki, A. S. Witherden, T. Kaneko, S. Sonnylal, C. D. Pusey, G. Bou-Gharios, R. M. Mason, Interstitial fibrosis is associated with increased COL1A2 transcription in AA-injured renal tubular epithelial cells in vivo. *Matrix Biol* **30**, 396-403 (2011).
153. C. Di Malta, L. Cinque, C. Settembre, Transcriptional Regulation of Autophagy: Mechanisms and Diseases. *Front Cell Dev Biol* **7**, 114 (2019).
154. M. C. Larsen, J. Lee, J. S. Jorgensen, C. R. Jefcoate, STARD1 Functions in Mitochondrial Cholesterol Metabolism and Nascent HDL Formation. Gene Expression and Molecular mRNA Imaging Show Novel Splicing and a 1:1 Mitochondrial Association. *Frontiers in Endocrinology* **11**, (2020).
155. U. N. Das, Arachidonic acid and other unsaturated fatty acids and some of their metabolites function as endogenous antimicrobial molecules: A review. *J Adv Res* **11**, 57-66 (2018).
156. E. Schlecker, A. Stojanovic, C. Eisen, C. Quack, C. S. Falk, V. Umansky, A. Cerwenka, Tumor-infiltrating monocytic myeloid-derived suppressor cells mediate CCR5-dependent recruitment of regulatory T cells favoring tumor growth. *J Immunol* **189**, 5602-5611 (2012).
157. T. Liu, A. Xiang, T. Peng, A. C. Doran, K. J. Tracey, B. J. Barnes, I. Tabas, M. Son, B. Diamond, HMGB1-C1q complexes regulate macrophage function by switching between leukotriene and specialized proresolving mediator biosynthesis. *Proc Natl Acad Sci U S A* **116**, 23254-23263 (2019).
158. S. S. Bohlson, S. D. O'Conner, H. J. Hulsebus, M. M. Ho, D. A. Fraser, Complement, c1q, and c1q-related molecules regulate macrophage polarization. *Front Immunol* **5**, 402 (2014).
159. N. Kaushal, A. K. Kudva, A. D. Patterson, C. Chiaro, M. J. Kennett, D. Desai, S. Amin, B. A. Carlson, M. T. Cantorna, K. S. Prabhu, Crucial role of macrophage selenoproteins in experimental colitis. *J Immunol* **193**, 3683-3692 (2014).
160. R. Mashima, K. Saeki, D. Aki, Y. Minoda, H. Takaki, T. Sanada, T. Kobayashi, H. Aburatani, Y. Yamanashi, A. Yoshimura, FLN29, a novel interferon- and LPS-inducible gene acting as a negative regulator of toll-like receptor signaling. *J Biol Chem* **280**, 41289-41297 (2005).
161. T. Sanada, G. Takaesu, R. Mashima, R. Yoshida, T. Kobayashi, A. Yoshimura, FLN29 deficiency reveals its negative regulatory role in the Toll-like receptor (TLR) and retinoic acid-inducible gene I (RIG-I)-like helicase signaling pathway. *J Biol Chem* **283**, 33858-33864 (2008).
162. C. Cui, M. Su, Y. Lin, L. Lai, A CD300c-Fc Fusion Protein Inhibits T Cell Immunity. *Front Immunol* **9**, 2657 (2018).
163. M. S. Schappe, K. Szteyn, M. E. Stremska, S. K. Mendu, T. K. Downs, P. V. Seegren, M. A. Mahoney, S. Dixit, J. K. Krupa, E. J. Stipes, J. S. Rogers, S. E. Adamson, N. Leitinger, B. N. Desai, Chanzyme TRPM7 Mediates the Ca(2+) Influx Essential for Lipopolysaccharide-Induced Toll-Like Receptor 4 Endocytosis and Macrophage Activation. *Immunity* **48**, 59-74 e55 (2018).
164. F. Granucci, The Family of LPS Signal Transducers Increases: the Arrival of Chanzymes. *Immunity* **48**, 4-6 (2018).
165. K. Neumann, M. Castineiras-Vilarino, U. Hockendorf, N. Hannesschlager, S. Lemeer, D. Kupka, S. Meyermann, M. Lech, H. J. Anders, B. Kuster, D. H. Busch, A. Gewies, R.

Naumann, O. Gross, J. Ruland, Clec12a is an inhibitory receptor for uric acid crystals that regulates inflammation in response to cell death. *Immunity* **40**, 389-399 (2014).

166. V. Kumar, P. Cheng, T. Condamine, S. Mony, L. R. Languino, J. C. McCaffrey, N. Hockstein, M. Guarino, G. Masters, E. Penman, F. Denstman, X. Xu, D. C. Altieri, H. Du, C. Yan, D. I. Gabrilovich, CD45 Phosphatase Inhibits STAT3 Transcription Factor Activity in Myeloid Cells and Promotes Tumor-Associated Macrophage Differentiation. *Immunity* **44**, 303-315 (2016).

167. L. Zhong, X. F. Chen, Z. L. Zhang, Z. Wang, X. Z. Shi, K. Xu, Y. W. Zhang, H. Xu, G. Bu, DAP12 Stabilizes the C-terminal Fragment of the Triggering Receptor Expressed on Myeloid Cells-2 (TREM2) and Protects against LPS-induced Pro-inflammatory Response. *J Biol Chem* **290**, 15866-15877 (2015).

168. L. Wang, R. A. Gordon, L. Huynh, X. Su, K. H. Park Min, J. Han, J. S. Arthur, G. D. Kalliolias, L. B. Ivashkiv, Indirect inhibition of Toll-like receptor and type I interferon responses by ITAM-coupled receptors and integrins. *Immunity* **32**, 518-530 (2010).

169. A. L. Gavin, D. Huang, C. Huber, A. Martensson, V. Tardif, P. D. Skog, T. R. Blane, T. C. Thinnes, K. Osborn, H. S. Chong, F. Kargaran, P. Kimm, A. Zeitjian, R. L. Sielski, M. Briggs, S. R. Schulz, A. Zarpellon, B. Cravatt, E. S. Pang, J. Teijaro, J. C. de la Torre, M. O'Keeffe, H. Hochrein, M. Damme, L. Teyton, B. R. Lawson, D. Nemazee, PLD3 and PLD4 are single-stranded acid exonucleases that regulate endosomal nucleic-acid sensing. *Nat Immunol* **19**, 942-953 (2018).

170. S. Bournazos, T. T. Wang, J. V. Ravetch, The Role and Function of Fcgamma Receptors on Myeloid Cells. *Microbiol Spectr* **4**, (2016).

171. F. Borrego, The CD300 molecules: an emerging family of regulators of the immune system. *Blood* **121**, 1951-1960 (2013).

172. E. C. Suter, E. M. Schmid, A. R. Harris, E. Voets, B. Francica, D. A. Fletcher, Antibody:CD47 ratio regulates macrophage phagocytosis through competitive receptor phosphorylation. *Cell Rep* **36**, 109587 (2021).

173. M. Tomihari, J. S. Chung, H. Akiyoshi, P. D. Cruz, Jr., K. Ariizumi, DC-HIL/glycoprotein Nmb promotes growth of melanoma in mice by inhibiting the activation of tumor-reactive T cells. *Cancer Res* **70**, 5778-5787 (2010).

174. M. J. Rauh, L. M. Sly, J. Kalesnikoff, M. R. Hughes, L. P. Cao, V. Lam, G. Krystal, The role of SHIP1 in macrophage programming and activation. *Biochem Soc Trans* **32**, 785-788 (2004).

175. P. Dobranowski, L. M. Sly, SHIP negatively regulates type II immune responses in mast cells and macrophages. *J Leukoc Biol*, (2018).

176. J. Jung, H. Zeng, T. Horng, Metabolism as a guiding force for immunity. *Nat Cell Biol* **21**, 85-93 (2019).

177. A. M. Lesokhin, T. M. Hohl, S. Kitano, C. Cortez, D. Hirschhorn-Cymerman, F. Avogadri, G. A. Rizzuto, J. J. Lazarus, E. G. Pamer, A. N. Houghton, T. Merghoub, J. D. Wolchok, Monocytic CCR2(+) myeloid-derived suppressor cells promote immune escape by limiting activated CD8 T-cell infiltration into the tumor microenvironment. *Cancer Res* **72**, 876-886 (2012).

178. V. Hosur, M. L. Farley, L. M. Burzenski, L. D. Shultz, M. V. Wiles, ADAM17 is essential for ectodomain shedding of the EGF-receptor ligand amphiregulin. *FEBS Open Bio* **8**, 702-710 (2018).

179. J. Scheller, A. Chalaris, C. Garbers, S. Rose-John, ADAM17: a molecular switch to control inflammation and tissue regeneration. *Trends Immunol* **32**, 380-387 (2011).
180. E. M. Hanson, V. K. Clements, P. Sinha, D. Ilkovitch, S. Ostrand-Rosenberg, Myeloid-derived suppressor cells down-regulate L-selectin expression on CD4+ and CD8+ T cells. *J Immunol* **183**, 937-944 (2009).
181. S. Xu, Z. Sun, Y. Sun, J. Zhu, X. Li, X. Zhang, G. Shan, Z. Wang, H. Liu, X. Wu, IL-15 and dendritic cells induce proliferation of CD4+CD25+ regulatory T cells from peripheral blood. *Immunol Lett* **140**, 59-67 (2011).
182. M. Salem, M. A. El Azreq, J. Pelletier, B. Robaye, F. Aoudjit, J. Sevigny, Exacerbated intestinal inflammation in P2Y6 deficient mice is associated with Th17 activation. *Biochim Biophys Acta Mol Basis Dis* **1865**, 2595-2605 (2019).
183. M. Cortes, L. Sanchez-Moral, O. de Barrios, M. J. Fernandez-Acenero, M. C. Martinez-Campanario, A. Esteve-Codina, D. S. Darling, B. Gyorffy, T. Lawrence, D. C. Dean, A. Postigo, Tumor-associated macrophages (TAMs) depend on ZEB1 for their cancer-promoting roles. *EMBO J* **36**, 3336-3355 (2017).
184. H. Imamichi, I. Sereti, H. C. Lane, IL-15 acts as a potent inducer of CD4(+)CD25(hi) cells expressing FOXP3. *Eur J Immunol* **38**, 1621-1630 (2008).
185. K. Balabanian, A. Levoye, L. Klemm, B. Lagane, O. Hermine, J. Harriague, F. Baleux, F. Arenzana-Seisdedos, F. Bachelerie, Leukocyte analysis from WHIM syndrome patients reveals a pivotal role for GRK3 in CXCR4 signaling. *J Clin Invest* **118**, 1074-1084 (2008).
186. D. L. Rebolledo, K. E. Lipson, E. Brandan, Driving fibrosis in neuromuscular diseases: Role and regulation of Connective tissue growth factor (CCN2/CTGF). *Matrix Biol Plus* **11**, 100059 (2021).
187. J. Wan, G. Zhang, X. Li, X. Qiu, J. Ouyang, J. Dai, S. Min, Matrix Metalloproteinase 3: A Promoting and Destabilizing Factor in the Pathogenesis of Disease and Cell Differentiation. *Front Physiol* **12**, 663978 (2021).
188. T. Frohlich, E. Kemter, F. Flenkenthaler, N. Klymiuk, K. A. Otte, A. Blutke, S. Krause, M. C. Walter, R. Wanke, E. Wolf, G. J. Arnold, Progressive muscle proteome changes in a clinically relevant pig model of Duchenne muscular dystrophy. *Sci Rep* **6**, 33362 (2016).
189. T. Xie, Y. Wang, N. Deng, G. Huang, F. Taghavifar, Y. Geng, N. Liu, V. Kulur, C. Yao, P. Chen, Z. Liu, B. Stripp, J. Tang, J. Liang, P. W. Noble, D. Jiang, Single-Cell Deconvolution of Fibroblast Heterogeneity in Mouse Pulmonary Fibrosis. *Cell Rep* **22**, 3625-3640 (2018).
190. Y. Enomoto, S. Matsushima, K. Shibata, Y. Aoshima, H. Yagi, S. Meguro, H. Kawasaki, I. Kosugi, T. Fujisawa, N. Enomoto, N. Inui, Y. Nakamura, T. Suda, T. Iwashita, LTBP2 is secreted from lung myofibroblasts and is a potential biomarker for idiopathic pulmonary fibrosis. *Clin Sci (Lond)* **132**, 1565-1580 (2018).
191. S. Klee, M. Lehmann, D. E. Wagner, H. A. Baarsma, M. Konigshoff, WISP1 mediates IL-6-dependent proliferation in primary human lung fibroblasts. *Sci Rep* **6**, 20547 (2016).
192. A. Bleve, B. Durante, A. Sica, F. M. Consonni, Lipid Metabolism and Cancer Immunotherapy: Immunosuppressive Myeloid Cells at the Crossroad. *Int J Mol Sci* **21**, (2020).
193. M. Ouzounova, E. Lee, R. Piranlioglu, A. El Andaloussi, R. Kolhe, M. F. Demirci, D. Marasco, I. Asm, A. Chadli, K. A. Hassan, M. Thangaraju, G. Zhou, A. S. Arbab, J. K.

Cowell, H. Korkaya, Monocytic and granulocytic myeloid derived suppressor cells differentially regulate spatiotemporal tumour plasticity during metastatic cascade. *Nat Commun* **8**, 14979 (2017).
194. S. Gong, C. Xu, L. Wang, Y. Liu, D. Owusu, B. A. Bailey, Y. Li, K. Wang, Genetic association analysis of polymorphisms in PSD3 gene with obesity, type 2 diabetes, and HDL cholesterol. *Diabetes Res Clin Pract* **126**, 105-114 (2017).
195. R. Csepanyi-Komi, G. Sirokmany, M. Geiszt, E. Ligeti, ARHGAP25, a novel Rac GTPase-activating protein, regulates phagocytosis in human neutrophilic granulocytes. *Blood* **119**, 573-582 (2012).
196. G. Solinas, S. Schiarea, M. Liguori, M. Fabbri, S. Pesce, L. Zammataro, F. Pasqualini, M. Nebuloni, C. Chiabrando, A. Mantovani, P. Allavena, Tumor-conditioned macrophages secrete migration-stimulating factor: a new marker for M2-polarization, influencing tumor cell motility. *J Immunol* **185**, 642-652 (2010).
197. S. Ugel, F. De Sanctis, S. Mandruzzato, V. Bronte, Tumor-induced myeloid deviation: when myeloid-derived suppressor cells meet tumor-associated macrophages. *J Clin Invest* **125**, 3365-3376 (2015).
198. C. Zhu, J. M. Kros, M. van der Weiden, P. Zheng, C. Cheng, D. A. Mustafa, Expression site of P2RY12 in residential microglial cells in astrocytomas correlates with M1 and M2 marker expression and tumor grade. *Acta Neuropathol Commun* **5**, 4 (2017).
199. R. Pasternack, C. Buchold, R. Jahnig, C. Pelzer, M. Sommer, A. Heil, P. Florian, G. Nowak, U. Gerlach, M. Hils, Novel inhibitor ZED3197 as potential drug candidate in anticoagulation targeting coagulation FXIIIa (F13a). *J Thromb Haemost* **18**, 191-200 (2020).
200. D. Baitsch, H. H. Bock, T. Engel, R. Telgmann, C. Muller-Tidow, G. Varga, M. Bot, J. Herz, H. Robenek, A. von Eckardstein, J. R. Nofer, Apolipoprotein E induces antiinflammatory phenotype in macrophages. *Arterioscler Thromb Vasc Biol* **31**, 1160-1168 (2011).
201. K. Sumida, D. Wakita, Y. Narita, K. Masuko, S. Terada, K. Watanabe, T. Satoh, H. Kitamura, T. Nishimura, Anti-IL-6 receptor mAb eliminates myeloid-derived suppressor cells and inhibits tumor growth by enhancing T-cell responses. *Eur J Immunol* **42**, 2060-2072 (2012).
202. C. Bayly-Jones, S. S. Pang, B. A. Spicer, J. C. Whisstock, M. A. Dunstone, Ancient but Not Forgotten: New Insights Into MPEG1, a Macrophage Perforin-Like Immune Effector. *Front Immunol* **11**, 581906 (2020).
203. M. E. Benoit, E. V. Clarke, P. Morgado, D. A. Fraser, A. J. Tenner, Complement protein C1q directs macrophage polarization and limits inflammasome activity during the uptake of apoptotic cells. *J Immunol* **188**, 5682-5693 (2012).
204. S. Morioka, K. Nigorikawa, E. Okada, Y. Tanaka, Y. Kasuu, M. Yamada, S. Kofuji, S. Takasuga, H. Nakanishi, T. Sasaki, K. Hazeki, TMEM55a localizes to macrophage phagosomes to downregulate phagocytosis. *J Cell Sci* **131**, (2018).
