# Supplementary material for: Single nuclei transcriptomics of muscle reveals intra-muscular cell dynamics linked to dystrophin loss and rescue
Source: Commun Biol. 2022 Sep 19;5:989. doi: 10.1038/s42003-022-03938-0 (PMC9485160; doi:10.1038/s42003-022-03938-0)
Supplement: Supplementary file 2 — Description of Additional Supplementary Files [file 42003_2022_3938_MOESM2_ESM.pdf]

Description of Additional Supplementary Files

File name: Supplementary Data 1
Description: Differentially Expressed genes mdx vs mdx e23AON vs WT mouse data.

File name: Supplementary Data 2
Description: Seurat findallmarkers files for mouse data.

File name: Supplementary Data 3
Description: Genes and References.

File name: Supplementary Data 4
Description: M2MDSC_vs_MDSC differentially expressed genes .

File name: Supplementary Data 5
Description: MDSC vs. M1.M2 transitional differentially expressed genes.

File name: Supplementary Data 6
Description: FB POSTN1 vs. FB CCL11 differentially expressed genes.

File name: Supplementary Data 7
Description: numerical data Fig. 6a.

File name: Supplementary Data 8
Description: numerical data Fig. 7c.

File name: Supplementary Data 9
Description: Seurat findallmarkers files for human data.

File name: Supplementary Data 10
Description: Differentially expressed genes DMD vs control human data.
