# Supplementary material for: Single nuclei transcriptomics of muscle reveals intra-muscular cell dynamics linked to dystrophin loss and rescue
Source: Commun Biol. 2022 Sep 19;5:989. doi: 10.1038/s42003-022-03938-0 (PMC9485160; doi:10.1038/s42003-022-03938-0)
Supplement: Supplementary file 13 — nr-reporting-summary [file 42003_2022_3938_MOESM13_ESM.pdf]

nature research

Corresponding author(s): M. Carrie Miceli and Stan Nelson

Last updated by author(s): Aug 23, 2022

Reporting Summary

Nature Research wishes to improve the reproducibility of the work that we publish. This form provides structure for consistency and transparency in reporting. For further information on Nature Research policies, see our Editorial Policies and the Editorial Policy Checklist.

Statistics

For all statistical analyses, confirm that the following items are present in the figure legend, table legend, main text, or Methods section.

n/a	Confirmed	
☐	☒	The exact sample size (*n*) for each experimental group/condition, given as a discrete number and unit of measurement
☐	☒	A statement on whether measurements were taken from distinct samples or whether the same sample was measured repeatedly
☐	☒	The statistical test(s) used AND whether they are one- or two-sided<br>*Only common tests should be described solely by name; describe more complex techniques in the Methods section.*
☐	☒	A description of all covariates tested
☐	☒	A description of any assumptions or corrections, such as tests of normality and adjustment for multiple comparisons
☐	☒	A full description of the statistical parameters including central tendency (e.g. means) or other basic estimates (e.g. regression coefficient) AND variation (e.g. standard deviation) or associated estimates of uncertainty (e.g. confidence intervals)
☐	☒	For null hypothesis testing, the test statistic (e.g. *F*, *t*, *r*) with confidence intervals, effect sizes, degrees of freedom and *P* value noted<br>*Give P values as exact values whenever suitable.*
☐	☒	For Bayesian analysis, information on the choice of priors and Markov chain Monte Carlo settings
☐	☒	For hierarchical and complex designs, identification of the appropriate level for tests and full reporting of outcomes
☐	☒	Estimates of effect sizes (e.g. Cohen's *d*, Pearson's *r*), indicating how they were calculated

Our web collection on statistics for biologists contains articles on many of the points above.

Software and code

Policy information about availability of computer code

Data collection	Cellranger 3.0.2: compiles raw sequences data from Ilumina sequencing.
Data analysis	Cellranger 3.0.2: initial processing of raw fastq files after sequencing, creation of gene by cell matrix files, initial clustering<br>DoubletFinder_V3: Computational approach to identifying doublets in single cell data<br>Seurat 3.2.3: Comprehensive single cell data analysis software suite<br>R 3.3.2: Statistical software<br>Slingshot 1.7.2: Pseudotime lineage tracing program, for determining developmental relatedness of celltypes<br>Harmony 0.1.0: Batch correction for single cell RNAseq analysis<br>Custom code: Extracts reads from fastq files that show exon-exon junction over regions of interest

For manuscripts utilizing custom algorithms or software that are central to the research but not yet described in published literature, software must be made available to editors and reviewers. We strongly encourage code deposition in a community repository (e.g. GitHub). See the Nature Research guidelines for submitting code & software for further information.

Data

Policy information about availability of data

All manuscripts must include a data availability statement. This statement should provide the following information, where applicable:

- Accession codes, unique identifiers, or web links for publicly available datasets
- A list of figures that have associated raw data
- A description of any restrictions on data availability

The datasets generated and analyzed in this study are available in the NCBI Sequence Read Archive

Field-specific reporting

Please select the one below that is the best fit for your research. If you are not sure, read the appropriate sections before making your selection.

☒ Life sciences ☐ Behavioural & social sciences ☐ Ecological, evolutionary & environmental sciences

For a reference copy of the document with all sections, see nature.com/documents/nr-reporting-summary-flat.pdf

Life sciences study design

All studies must disclose on these points even when the disclosure is negative.

Sample size	Mouse samples for single nuclei sequencing, were selected from prior published data of mdx(n=4), mdx treated with exon23 AON(n=4), and genetically matched WT mice (n=3). 2 healthy humans and 3 Duchenne muscular dystrophy samples were selected to demonstrate the applicability of the method for single nuclei purification to human muscle disease.
Data exclusions	All snRNASEQ datasets generated were included in analyses, individual nuclei with <200 unique reads in fastq files were excluded from all analyses as failures of library generation within the method, along with predicted doublets identified by DoubletFinder and mitochondrial and ribosomal genes.
Replication	Each nuclei type is observed by hundreds of individual nuclei in each experimental condition. These similarities between species provide a great deal of confidence in our findings. Additionally, findings reported here are not specific to outlier individuals but commonly share across most, if not all, individuals in a group (i.e. untreated mdx, treated mdx, and wild type). Statistical test underwent multiple testing corrections to reduce likelihood of false positives. One sample was run two times independently. This sample did not deviate significantly in it's clustering characteristics, nor were differences which would alter results detected before and after running harmony for batch correction, thus we believe our data to be replicable.
Randomization	Prior live animal work was designed to ensure covariates were consisted across all animals besides drug treatment (i.e. same age, diet, living conditions, and genetic background). Wild type mice were matched to the same genetic background on which the mdx mutation originated.
Blinding	Investigators were not blinded to group identities.

Reporting for specific materials, systems and methods

We require information from authors about some types of materials, experimental systems and methods used in many studies. Here, indicate whether each material, system or method listed is relevant to your study. If you are not sure if a list item applies to your research, read the appropriate section before selecting a response.

Materials & experimental systems

n/a	Involved in the study	
☐	☒	Antibodies
☒	☐	Eukaryotic cell lines
☒	☐	Palaeontology and archaeology
☐	☒	Animals and other organisms
☐	☒	Human research participants
☒	☐	Clinical data
☒	☐	Dual use research of concern

Methods

n/a	Involved in the study	
☒	☐	ChIP-seq
☒	☐	Flow cytometry
☒	☐	MRI-based neuroimaging

Antibodies

Antibodies used	Antibodies used were as follows: anti-CD45; rat, 30-F11 (ebiosicences), CD206; goat poly clonal (R&D), POSTN1; Rabbit poly clonal, (Thermo Fisher) , MYH2; 8F72C8 (Sigma-Aldrich), Rabbit anti-goat Alexa546 polyclonal (Invitrogen), donkey anti-rat Alexa 488 (Invitrogen), and Donkey anti-rabbit dyelight 550 (Thermo Fisher).
Validation	All antibodies validated by the manufacturer for the species indicated. All well known clones. Muscle related antibodies were identified from the following publication:<br><br>Sawano, S., Komiya, Y., Ichitsubo, R., Ohkawa, Y., Nakamura, M., Tatsumi, R., Ikeuchi, Y., Mizunoya, W., 2016. A One-Step Immunostaining Method to Visualize Rodent Muscle Fiber Type within a Single Specimen. PLOS ONE 11, e0166080.. doi:10.1371/journal.pone.0166080

Animals and other organisms

Policy information about studies involving animals; ARRIVE guidelines recommended for reporting animal research

Laboratory animals	Tibialis Anterior Muscle collected from prior mouse experiments involving 11 mice: Wild type = C57BL/10ScSnJ, mdx = C57BL/10ScSn-Dmd(mdx)/J, 6 months , males
Wild animals	None
Field-collected samples	None
Ethics oversight	UCLA protocol ARC # 2011-021-21

Note that full information on the approval of the study protocol must also be provided in the manuscript.

Human research participants

Policy information about studies involving human research participants

Population characteristics	Duchenne muscular dystrophy patients, males, ages 7 - 22<br>Healthy controls are over 18 years of age.
Recruitment	Healthy Subjects were recruited by flyer for participation in a core needle muscle biopsy over age 18 years. Individuals with Duchenne are recruited from UCLA clinic patients for participation in clinical biospecimen research.
Ethics oversight	UCLA IRB approvals 18-001366 or 19-00090

Note that full information on the approval of the study protocol must also be provided in the manuscript.
